# Engineered bacteria to accelerate wound healing: an adaptive, randomised, double-blind, placebo-controlled, first-in-human phase 1 trial

**DOI:** 10.1016/j.eclinm.2023.102014

**Published:** 2023-05-25

**Authors:** Emelie Öhnstedt, Evelina Vågesjö, Andreas Fasth, Hava Lofton Tomenius, Pia Dahg, Sofia Jönsson, Nisha Tyagi, Mikael Åström, Zhanar Myktybekova, Lovisa Ringstad, Margareth Jorvid, Peter Frank, Per Hedén, Stefan Roos, Mia Phillipson

**Affiliations:** a Ilya Pharma AB, Dag Hammarskjölds Väg 30, 752 37 Uppsala, Sweden; b Uppsala University, Department of Medical Cell Biology, 751 23 Uppsala, Sweden; c Sofiahemmet, Valhallavägen 91, 114 86 Stockholm, Sweden; d Swedish University of Agricultural Sciences, Department of Molecular Sciences, Uppsala BioCenter, 750 07 Uppsala, Sweden; e Science for Life Laboratory, Uppsala University, Sweden

**Keywords:** CXCL12, ILP100, Limosilactobacillus, Phase 1 clinical trial, Immunotherapy, SITUSAFE

## Abstract

**Background:**

Impaired wound healing is a growing medical problem and very few approved drugs with documented clinical efficacy are available. CXCL12-expressing lactic acid bacteria, *Limosilactobacillus reuteri* (ILP100-Topical), has been demonstrated to accelerate wound healing in controlled preclinical models. In this first-in-human study, the primary objective was to determine safety and tolerability of the drug candidate ILP100-Topical, while secondary objectives included assessments of clinical and biologic effects on wound healing by traditionally accepted methods and explorative and traceable assessments.

**Methods:**

SITU-SAFE is an adaptive, randomised, double-blind, placebo-controlled, first-in-human phase 1 trial (EudraCT 2019-000680-24) consisting of a single (SAD) and a multiple ascending dose (MAD) part of three dose cohorts each. The study was performed at the Phase 1 Unit, Uppsala University Hospital, Uppsala, Sweden. Data in this article were collected between Sep 20th, 2019 and Oct 20th 2021. In total 240 wounds were induced on the upper arms in 36 healthy volunteers. SAD: 12 participants, 4 wounds (2/arm), MAD: 24 participants, 8 wounds (4/arm). Wounds in each participant were randomised to treatment with placebo/saline or ILP100-Topical.

**Findings:**

In all individuals and doses, ILP100-Topical was safe and well-tolerated with no systemic exposure. A combined cohort analysis showed a significantly larger proportion of healed wounds (p = 0.020) on Day 32 by multi-dosing of ILP100-Topical when compared to saline/placebo (76% (73/96) and 59% (57/96) healed wounds, respectively). In addition, time to first registered healing was shortened by 6 days on average, and by 10 days at highest dose. ILP100-Topical increased the density of CXCL12^+^ cells in the wounds and local wound blood perfusion.

**Interpretation:**

The favourable safety profile and observed effects on wound healing support continued clinical development of ILP100-Topical for the treatment of complicated wounds in patients.

**Funding:**

Ilya Pharma AB (Sponsor), H2020 SME Instrument Phase II (#804438), Knut and Alice Wallenberg foundation.

**Research in context**

**Evidence before this study**

We searched PubMed for original articles, meta-analyses, and systematic reviews published until April 25th, 2023, describing the role of CXCL12-α in regeneration search terms included but were not limited to: SDF-1, CXCL12, regeneration, wound. At the start of the study in 2019, therapeutic functions to promote wound healing had been successfully addressed in preclinical models using genetically modified cells or bacteria that delivered CXCL12 locally. There are to our knowledge no reports of CXCL12 being tested in human wounds prior to this study.

**Added value of this study**

To our knowledge, this study is the first to provide support for safety and effects on wound healing by the novel first-in-class drug candidate with therapeutic CXCL12-α (ILP100-Topical) in a blinded, randomised, and placebo-controlled clinical trial setting. In addition, we demonstrate that this newly designed biotechnological platform enables delivery of proteins with short half-life, e.g. chemokines such as CXCL12-α, in a clinical use, and offers a novel approach for immunotherapies with local effects.

**Implications of all the available evidence**

No safety or tolerability issues were identified following treatment with ILP100-Topical to induced wounds. Clinical effect of accelerated wound healing was observed for the highest dose and when pooling data from all three multidose cohorts. The favourable safety profile and observed effect together support continued clinical development of ILP100-Topical for the treatment of difficult skin wounds in patients.

## Introduction

Complicated or non-healing wounds, encompassing wounds that do not heal for 4 or more weeks with standard of care, are a growing medical problem associated with metabolic diseases and aging.^1^, ^2^, ^3^, ^4^ These problematic wounds negatively impact life quality and reduce life expectancy, and they often become infected and increase the risk for sepsis. There is a high unmet need for effective therapies, as there are very few available therapeutics with proven efficacy of accelerated wound healing. Instead, antibiotics are being overused in these patients, and up to 75% receive systemic antibiotics despite often lacking documented clinical infection.^5^^,^^6^

Wound healing is driven by cells of the immune system regulated by signals from the wound microenvironment.^7^, ^8^, ^9^ Immunomodulatory drugs are currently transforming oncology and autoimmune diseases, while therapeutic targeting of immune cells within wounds has not yet been successful. This is at least in part due to that topical administration is hampered by the proteolytic wound environment, which limits the bioavailability of candidate therapeutic molecules.^10^ Therefore, genetically modified bacteria producing, delivering, and stabilising immunomodulatory proteins within the wounds could be disruptive in the field of immunotherapy, as they enable the use of proteins with short half-lives as scalable therapeutics.

A first-in-class drug candidate, ILP100-Topical (emilimogene sigulactibac), was designed by engineering *Limosilactobacillus reuteri* R2LC (*L. reuteri* R2LC), a strain of non-human origin, to produce and release the human chemokine CXCL12-α on-site to the wound bed.^11^ Accelerated healing after topical delivery has been well-documented in multiple non-clinical studies, depends on increased numbers of wound macrophages of a restorative phenotype expressing TGF-β, and a favourable safety profile was demonstrated.^5^^,^^12^ Here, we present results from the randomised, blinded, and placebo-controlled first-in-human study designed to primarily assess safety and tolerability of ILP100-Topical, whereas the secondary and exploratory objectives aimed to evaluate clinical and biologic effects on wound healing. To complement and validate the conventional assessments performed by Investigators during visits, blinded and high-resolution wound imaging techniques were used, which provided objective analyses of healing in fully traceable and reproducible data sets. This pioneering study demonstrates a favourable safety profile together with proven clinical and biologic effects on accelerated wound healing, which supports the continued clinical development of ILP100-Topical, a new modality and local immunotherapeutic.

## Methods

### Study design

This single-centre adaptive, randomised, double-blind, placebo-controlled, first-in-human phase 1 trial (SITU-SAFE) was conducted at the Phase 1 Unit, Uppsala University Hospital, Sweden, in 240 induced skin wounds in 36 healthy volunteers. The study included a treatment phase followed by an assessment phase running up to 6 weeks after last dose and an ongoing 5-year long-term follow up. The results presented in this paper were collected between September 20th, 2019 and October 20th, 2021 include results up to 13 months, i.e. 12 months follow up after last dose in the MAD part. The primary objective was to determine the safety and tolerability profile, whereas other objectives included assessments of clinical and biologic effects on wound healing, as well as presence and biodistribution of ILP100-Topical. ILP100-Topical consists of *L. reuteri* R2LC genetically modified with the pSIP_CXCL12-α plasmid to express CXCL12-α, hereunder referred to as CXCL12, following induction by the activation peptide SppIP.^11^^,^^13^^,^^14^ The ready-to-use drug product consists of *L. reuteri* R2LC carrying the pSIP_CXCL12 plasmid reconstituted with SppIP-containing buffer. As a control within each participant, placebo (SppIP-containing buffer), or saline (NaCl 0.9%) was used. The study was designed to comprise a single ascending dose (SAD) part of three cohorts, and a multiple ascending dose (MAD) part of another three cohorts, where safety confirmation of the SAD part preceded MAD initiation (Supplementary Fig. S1).

The studies were undertaken in accordance with Good Clinical Practice and the Declaration of Helsinki, and with approval of the Swedish Ethical Review Authority (Approval no. 2019-02802) and the Medical Product Agency in Uppsala, Sweden. Informed consent was obtained from the study individuals. The trial is registered in EudraCT (2019-000680-24).

### Participants

Healthy male and female individuals aged 25–45 years who were willing to comply with the study procedures (experimental incision of 4 or 8 wounds in the SAD and MAD, respectively, equally distributed at the upper inner arms) and who have given written informed consent were considered eligible to participate in the study. Prior to consent, all individuals were given extensive information about the procedures and the potential risks with the study, such as punch biopsy procedure and risk of scarring. All individuals included had to understand and be willing to comply with study procedures. Individuals with a history of any bleeding disorder, including prolonged or habitual bleeding, individuals on blood-thinning medication or individuals with e.g. a tattoo or apparent skin abnormality on the upper inner arms were not included in the study, neither were pregnant or lactating women.

### Randomisation and masking

The Investigational medicinal products (IMPs) were prepared by unblinded pharmacists, masked in order to maintain the blind, and administered topically in volumes of 50 μl per wound to blindfolded individuals. A computer-generated randomisation list (SAS Proc Plan, SAS Version 9.4, Institute, Inc., Cary, NC, USA) was kept by the randomiser in a sealed envelope until database lock.

### Procedures

Enrolled individuals were admitted to the clinic on Day 1 for pre-dose safety assessments and full thickness wound punching (biopsy punch, 6 mm in diameter) on the ventral aspect of the upper arms (SAD: 2 wounds/arm; MAD: 4 wounds/arm) following treatment of local anaesthesia (injected Xylocaine 10 mg/mL) and cleaning of the area (70% ethanol) (Supplementary Fig. S1). The wounds were photographed in a standardised setting before treatment on Day 1, and at all subsequent visits. For assessment of wound healing, the non-epithelialised wound area was measured by the IEs using ImageJ Software (U. S. National Institutes of Health, USA). To address exploratory objectives, wounds of the MAD part were scanned using a 3D spectroscopic scanner to evaluate scar area, scar volume and scar redness (Cherry Imaging, Yokneam, Israel, Supplementary methods) and blood perfusion of the wound bed and adjacent skin was measured (Laser Speckle Contract Analysis, LASCA; Perimed AB, Järfälla, Sweden, Supplementary Fig. S2 and Supplementary methods).^15^^,^^16^ Wound biopsies were taken 48 h post-dosing in the SAD part for assessment of local mechanisms of action (Supplementary methods).

The SAD part of the study comprised of 3 sequential cohorts, each including 4 individuals with 2 experimentally induced wounds on each arm, in total 12 individuals and 48 wounds. For each individual, a single dose of ILP100-Topical (5 × 10^4^, 5 × 10^7^ or 1 × 10^9^ CFU/cm^2^ wound area in cohort 1, cohort 2 and cohort 3, respectively) and placebo were randomised to 2 wounds on the left arm and 2 wounds on the right arm, in a 1:1 ratio.

The MAD part comprised of 3 sequential cohorts, each including 8 individuals with 4 experimentally induced wounds on each arm, in total 24 individuals and 192 wounds. The IMP was randomised in a 4:2:2 ratio, with ILP100-Topical (cohort 1: 5 × 10^5^ CFU/cm^2^, cohort 2: 5 × 10^7^ CFU/cm^2^ and cohort 3: 1 × 10^9^ CFU/cm^2^) to 4 wounds on left or right arm, and placebo or saline to 2 wounds each on the arm on which wounds did not receive ILP100-Topical. Saline was used as a control to assess the potential effect on wound healing by the SppIP-containing buffer in placebo. Each wound was administered with repeated doses of IMP on Day 1, 2 and 3, followed by 3 times a week over the course of 3 weeks (10 doses in total).

## Outcomes

Clinical safety assessments were performed at visits and included adverse events (AEs), clinical laboratory parameters, vital signs, ECG, physical examination, local tolerability reactions, formation of anti-CXCL12 antibodies (ADA, supplementary methods), systemic exposure of CXCL12 in plasma (Supplementary methods), as well as presence of *L. reuteri* R2LC containing the pSIP_CXCL12 on the skin surrounding the wound, blood, and faeces (Supplementary methods).

Tolerability, clinical and biologic effects were assessed at each visit (SAD: Day 1, 2, 3, 7, 14, and at 6 weeks, 3 months and 12 months from start of treatment at Day 1; MAD: Day 1, 2, 3, 5, 8, 10, 12, 15, 17, 19, 21, 32, and at 6 weeks, 3 months and 12 months after last dose at Day 19). All assessments were blinded and occurred by on-site visual inspections of the wounds by the Principal Investigator (or co-investigator), as well as off-site by traceable evaluation and detailed wound area measurements from 2D photographs of all wounds by 3 Independent Evaluators (IEs) with expertise in wound healing. Tolerability was graded 0–3 according to predefined criteria based on wound appearance (wound and wound edge inflammation, surrounding skin inflammation, haemorrhage, presence of exudate, slough or necrotic tissue, granulation tissue, or hypergranulation). For the clinical effect on wound healing, a wound was defined as healed when the wound area was completely re-epithelialised and there were no dressing requirements, and if the assessments by one or more IEs deviated more than two steps on the 4-grade scale, the three IEs assembled to adjudicate the definitive grade. In addition, 3D scans were used to assess changes in scar size, while analyses of the mechanism of action included blood flow measurements (MAD part only) and molecular changes by histology and local CXCL12 levels by ELISA in the wound biopsies (SAD part only).

### Statistical analysis

The sample size was considered sufficient to provide adequate information for the primary and related safety and tolerability objectives. For detailed description about statistical analysis, please see supplementary methods. In the post-hoc analyses of the biologic and clinical effects on wound healing, Fisher's exact test and the Mann–Whitney test were used for comparing the different treatment groups for proportion healed wounds and average time to first registered healing. All descriptive summaries and pre-defined statistical analyses were performed using SAS Version 9.4 (SAS Institute Inc., Cary, NC, USA). Post-hoc analyses were performed using StatXact Version 11.1.0 (Cytel Inc., Waltham, MA, USA), SAS Version 9.4, and GraphPad Prism 9.1.1.225 (GraphPad Software, San Diego, CA, USA).

### Role of the funding source

Ilya Pharma AB is the Sponsor of the study fulfilling all sponsor responsibilities. The trial was in part supported by a grant from the European Commission, H2020 SME Instrument Phase II (#804438) and by Knut and Alice Wallenberg foundation.

Study Sponsor was responsible for the study design, analysis of data from 3D scanning and LASCA measurements, and decision to publish the data. Study report and data interpretation (except for 3D scanning and LASCA measurements) was performed by CRO and reviewed by the Sponsor.

EÖ, EV, AF, HLT, PD, SJ, NT, MÅ, ZM, LR, MJ, PF, PH, SR, and MP all had access to the dataset and accept responsibility for the decision to submit for publication.

## Results

**Fig. 1:**
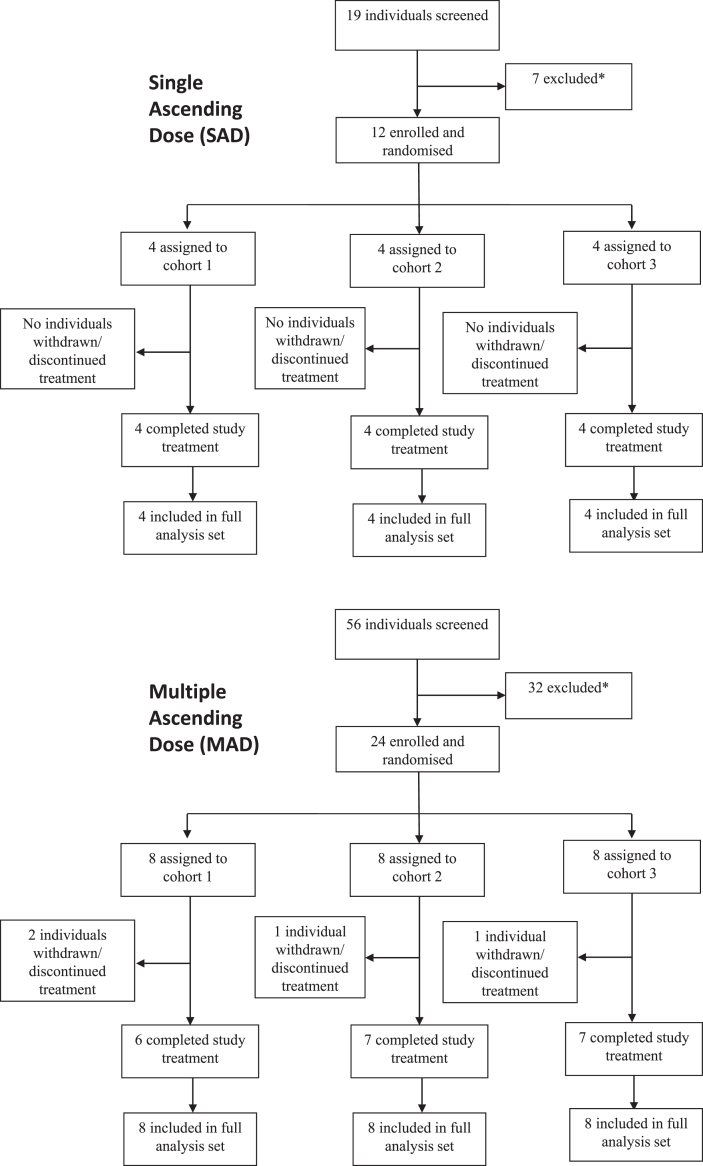
**Trial profile for SAD and MAD.** ∗Excluded = ineligible, reserves, or other.

Thirty-six healthy study individuals between 25 and 45 years old were enrolled at a Phase 1 Unit at Uppsala University Hospital, Uppsala, Sweden between 20th of September 2019 and 1st of October 2020 (Fig. 1). Baseline characteristics and demographics of the individuals are summarised in Supplementary Tables S1 and S2 for the SAD and MAD parts of the study, respectively.

The primary objective of the study was to determine the safety and tolerability profile. For all individuals, single- or multi-dosing of ILP100-Topical (1 and 10 administrations over 3 weeks, in the SAD and MAD, respectively) were considered safe and well-tolerated. No clinically significant changes from baseline of any parameters were detected during any visits. There were no serious adverse events or AEs leading to discontinuation from the study (Supplementary Tables S3 and S4). Overall, the AE profile of wounds treated with ILP100-Topical was comparable to that of wounds treated with placebo or saline (Table 1). For all cohorts, *L. reuteri* R2LC containing pSIP_CXCL12 was only identified on the skin surrounding wounds 1–2 days after treatment, no colonisation occurred, and *L. reuteri* R2LC containing pSIP_CXCL12 was not detected in blood or faeces at any time point. In addition, circulating levels of CXCL12 were not increased after single- or multi-dosing, and ADAs against CXCL12 could not be detected at any time point.

**Table 1:** Adverse events in Cohort 1, 2, and 3 up to 13 months follow-up (MAD).

	System organ class	LP100 (n = 32/cohort)		Placebo (n = 16/cohort)		Saline (n = 16/cohort)		Placebo+Saline (n = 32/cohort)		Total (n = 64/cohort)	
	Preferred term	n (%)	m	n (%)	m	n (%)	m	n (%)	m	n (%)	m
**Cohort 1**	General disorders and administration site conditions	13 (40%)	13	5 (31%)	5	9 (56%)	9	14 (44%)	14	27 (42%)	27
	Administration site eczema	11 (34%)	11	4 (25%)	4	8 (50%)	8	12 (38%)	12	23 (36%)	23
	Administration site inflammation	2 (6.3%)	2	1 (6.3%)	1	1 (6.3%)	1	2 (6.3%)	2	4 (6.3%)	4
	Infections and infestations	5 (16%)	5	5 (31%)	5	4 (25%)	4	9 (28%)	9	14 (22%)	14
	Eczema infected	0	0	2 (13%)	2	2 (13%)	2	4 (13%)	4	4 (6.3%)	4
	Wound infection	5 (16%)	5	3 (19%)	3	2 (13%)	2	5 (16%)	5	10 (16%)	10
	Skin and subcutaneous tissue disorders	6 (19%)	6	2 (13%)	2	2 (13%)	2	4 (13%)	4	10 (16%)	10
	Pruritus	2 (6.3%)	2	0	0	0	0	0	0	2 (3.1%)	2
	Skin mass	4 (13%)	4	2 (13%)	2	2 (13%)	2	4 (13%)	4	8 (13%)	8
	Injury, poisoning and procedural complications	2 (6.3%)	2	4 (25%)	5	1 (6.3%)	1	5 (16%)	6	7 (11%)	8
	Wound complication	0	0	4 (25%)	5	1 (6.3%)	1	5 (16%)	6	5 (8.0%)	6
	Wound haemorrhage	2 (6.3%)	2	0	0	0	0	0	0	2 (3.1%)	2
**Cohort 2**	Injury, poisoning and procedural complications	26 (81%)	31	9 (56%)	11	12 (75%)	13	21 (66%)	24	47 (73%)	55
	Wound complication	1 (3.1%)	1	0	0	0	0	0	0	1 (1.5%)	1
	Wound haemorrhage	25 (78%)	30	9 (56%)	11	12 (75%)	13	21 (66%)	24	46 (72%)	54
	General disorders and administration site conditions	0	0	0	0	1 (6.3%)	1	1 (3.1%)	1	1 (1.6%)	1
	Application site pruritus	0	0	0	0	1 (6.3%)	1	1 (3.1%)	1	1 (1.6%)	1
**Cohort 3**	Injury, poisoning and procedural complications	7 (22%)	9	5 (31%)	7	5 (31%)	6	10 (31%)	13	17 (27%)	22
	Wound complication	4 (13%)	4	3 (18%)	3	2 (13%)	2	5 (16%)	5	9 (14%)	9
	Wound hematoma	0	0	1 (6.3%)	1	0	0	1 (3.1%)	1	1 (1.6%)	1
	Wound haemorrhage	5 (16%)	5	3 (19%)	3	3 (19%)	4	6 (19%)	7	11 (17%)	12
	Infections and infestations	1 (3.1%)	1	0	0	0	0	0	0	1 (1.6%)	1
	Wound infection	1 (3.1%)	1	0	0	0	0	0	0	1 (1.6%)	1

Percentages are based on the number of wounds in the study period included in the full analysis set, n, number of wounds; m, number of events. Pre-treatments are not included.

In both SAD and MAD, transient inflammation of the wound and surrounding skin was observed to a higher degree for the highest ILP100-Topical levels (Supplementary Tables S5 and S6), while the prevalence of wound infections was similar between saline, placebo, and ILP100-Topical treated wounds (Table 1). Treatment with ILP100-Topical was associated with increased exudation in the two lowest doses and in the highest dose to the amount of slough/necrotic tissue, as assessed by the IEs, but not according to the Investigators (Supplementary Tables S8 and S9 and data not included). There were no evident associations between the amount of, granulation tissue, haemorrhage or hypergranulation between the different treatments in either SAD or MAD (Supplementary Tables S8, S10, and S11). Irrespective of treatment in cohort 1 in the MAD part, the Investigators reported eczema and inflammation of the skin in contact with the dressing (Table 1), which resulted in discontinuation of treatment of in total 28 wounds (Supplementary Table S4). The dressing type was therefore changed for cohort 2 and 3. ILP100 treatment did not increase wound rupture, as this was only reported for scars of two placebo-treated wounds.

**Fig. 2:**
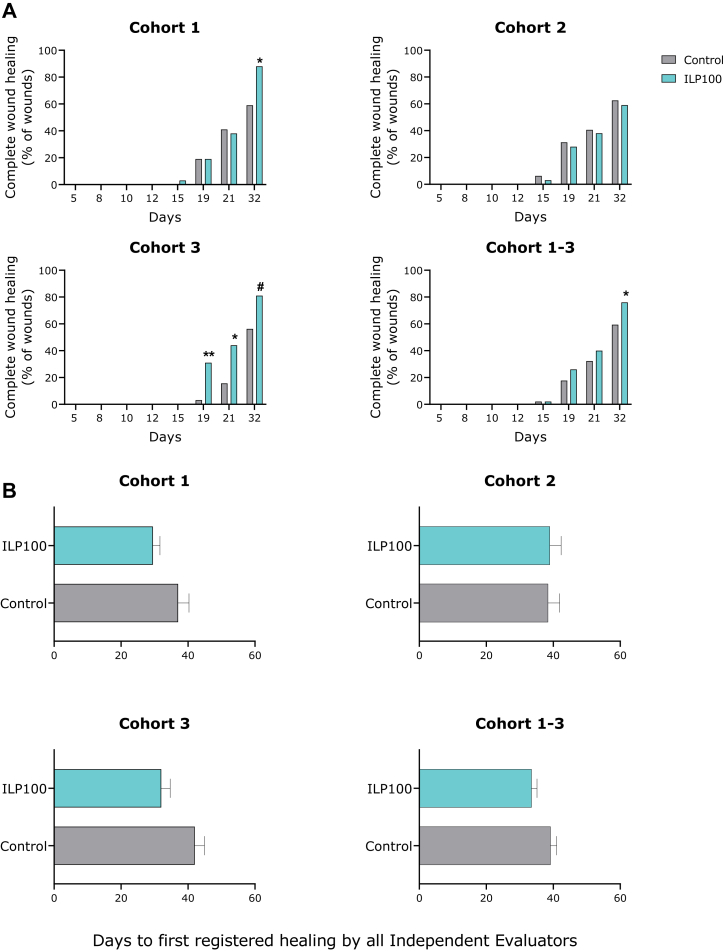
**Proportion of healed wounds at visits, and time to first registration of healed (days) for control-treated wounds and wounds treated with ILP100-Topical (MAD)**. The MAD cohorts treated with 5 × 10^5^ CFU/cm^2^ wound area (cohort 1), 5 × 10^7^ CFU/cm^2^ (cohort 2) and 1 × 10^9^ CFU/cm^2^ (cohort 3), respectively, were assessed for complete wound healing at visits. A) Wounds were defined as healed when the wound area was completely re-epithelialised (yes/no) by three blinded IEs in the MAD cohorts. #p = 0.058, ∗p ≤ 0.05, ∗∗p ≤ 0.01, compared to control. p-values (two-sided) are calculated by Fisher's exact test. B) The time point where a wound was first registered as healed at visits for the three cohorts of the MAD part, as well as for pooled cohorts. Mann–Whitney test ∗p ≤ 0.05, ∗∗p ≤ 0.01. Average time, error bars indicate standard error of the mean (SEM). Wounds with missing timepoint of wound healing or not judged as healed by the end of the study has been imputed as healed after 61 days being the next timepoint of assessment. Control group includes saline–or placebo-treated wounds.

Secondary objectives included assessments of clinical and biologic effects on wound healing. No differences in wound healing were detected between the saline–or placebo-treated wounds by either Investigators or IEs, and saline- and placebo-treatment were therefore pooled. The Investigators' assessments did not show any difference in wound healing between treatment groups. In the MAD part, the IEs’ assessments revealed treatment-related differences in wound healing at Days 32 in cohort 1, and at Days 19 and 21 (Day 32, p = 0.058) in cohort 3, where higher proportions of wounds treated with ILP100-Topical were assessed as healed by all three IEs compared to control-treated wounds (Fig. 2A). A pooled analysis of all cohorts showed that ILP100-Topical significantly improved wound healing compared to controls (p = 0.020), as 76% (73/96) of the ILP100-Topical treated wounds were healed at or prior to Day 32, as assessed by all IEs, compared to 59% (57/96) of control wounds (Fig. 2A). Further, when all doses/cohorts were pooled, the time to first registration of healed by all three IEs was on average shortened by 6 days by ILP100-Topical (p = 0.039) compared to controls. For the highest ILP100-Topical dose group, the time to first registration of healed was 10 days faster compared to controls (p = 0.0046, Fig. 2B). Similar results for time to wound healing and the proportion of healed wounds were obtained with paired statistical methods (data not included).

Irrespective of treatment, blood perfusion of the wound bed peaked at Day 8 (Supplementary Table S12), whereas wound edge perfusion decreased over time as the wounds gradually healed (Table 2) with the exception for cohort 1 where dressing-induced eczema and skin inflammation were reported (Table 1). Treatment with ILP100-Topical was found to increase wound edge blood perfusion dose dependently at Day 2 when compared to the control-treated wounds of cohorts 2 and 3, but not at Day 8 or 15 (Table 2).

**Table 2:** Wound edge blood perfusion analysed using non-invasive imaging Laser Speckle Contrast Analysis (LASCA) at Days 2, 8, and 15 in control- and ILP100-Topical-treated groups (MAD).

LASCA perfusion imaging	n, m	Day 2		Day 8		Day 15	
		Control	ILP100	Control	ILP100	Control	ILP100
**Cohort 1**	7–8, 26–32	43.8 ± 5.5	53.2 ± 5.6	34.8 ± 12.9	46.3 ± 12.1	30.4 ± 4.8	46.5 ± 10.4
**Cohort 2**	6–8, 24–32	56.1 ± 5.5	76.6 ± 6.0∗∗	31.6 ± 3.1	33.6 ± 3.0	10.9 ± 2.0	13.3 ± 1.8
**Cohort 3**	7–8, 28–32	37.1 ± 3.4	89.3 ± 5.4∗∗∗∗	32.2 ± 4.8	34.8 ± 4.4	5.4 ± 2.9	8.7 ± 2.7
**Cohort 1**–**3**	21–24, 84–96	45.7 ± 2.9	74.1 ± 3.6∗∗∗∗	32.7 ± 4.3	38.2 ± 4.3	17.1 ± 2.5	24.4 ± 4.5

The MAD cohorts treated with 5 × 10^5^ CFU/cm^2^ wound area (cohort 1), 5 × 10^7^ CFU/cm^2^ (cohort 2) and 1 × 10^9^ CFU/cm^2^ (cohort 3), respectively, were assessed for wound edge perfusion at visits Day 2, 8 and 15. Values are delta perfusion units (dPFU) and represent perfusion of the wound edge (skin area within 5 mm from the wound border) minus the reference non-wounded skin perfusion at the same image presented as Mean ± SEM. Controls represent saline–or placebo-treated wounds. n, number of individuals; m, number of wound frames analysed per group. Mann–Whitney test ∗∗p = 0.010, ∗∗∗∗≤0.0001.

Immunohistochemistry of wound biopsies from the SAD wounds revealed increased numbers by 59% of CXCL12^+^ cells in the wound edge dermis by the highest dose of ILP100-Topical (1018 ± 134 vs 1623 ± 315 for control and ILP100-treated wounds, respectively) (Table 3, Supplementary Fig. S3). No differences were detected for CXCL12 levels within tissue (Supplementary Table S13).

**Table 3:** CXCL12^+^ cells quantified following immunohistochemistry of wound edge biopsies 48 h following single dose administration of ILP100-Topical or placebo (SAD).

CXCL12^+^ cells in wound biopsies at 48 h	n, m	Higher density of CXCL12^+^ cells in ILP100 treated wounds	Placebo (m = 4/cohort)	ILP100 (m = 4/cohort)	Ratio
**Cohort 1**	4, 8	3 of 4 (75%)	479 ± 163	454 ± 126	0.95
**Cohort 2**	4, 8	1 of 4 (25.0%)	689 ± 52	485 ± 106	0.70
**Cohort 3**	4, 8	4 of 4 (100.0%)	1018 ± 134	1623 ± 315	1.59
**Cohort 1**–**3**	12, 24	8 of 12 (66.7%)	729 ± 94	846 ± 196	1.16

Data represents mean values (±SEM) of placebo-treated and ILP100-Topical-treated wounds compared within each subject in the cohort. N, number of individuals; m, number of wound biopsies analysed per treatment group.

Scar formation was assessed as normal for all healed wounds at all visits. The 3D spectroscopic scanning revealed no difference in scar areas between cohorts or treatments (Supplementary Table S14), while the sensitivity of scar volume scans did not allow for comparisons between treatment groups (Supplementary Tables S15 and S16). Scar redness normalised to skin colour was also assessed, but no differences between treatments could be detected (Supplementary Tables S17 and S18).

## Discussion

In this first-in-human trial, topical application of the first-in-class drug candidate ILP100-Topical was suggested to be safe and well-tolerated. In addition, multiple doses of ILP100-Topical supported clinical efficacy on wound healing, as demonstrated by a larger proportion of healed wounds from Day 19 and shortened time to first registered healing. Thus, genetically modified *L. reuteri* R2LC engineered to deliver and stabilise CXCL12 was suggested to be safe and effective in accelerating healing of induced wounds.

Therapeutic means to support healing has recently been focusing on altering the wound microenvironment by topical application of growth factors, plasma-derived products or cell therapies.^17^, ^18^, ^19^ These strategies are often hampered by restricted access of administered cells to wound tissue, and by limited bioavailability of therapeutic proteins due to high levels of proteases present in wounds. Another disruptive and recently recognised approach is to use genetically modified bacteria to deliver endogenous proteins to wounds.^20^ So far, two attempts have been reported to accelerate healing in mouse models: *Lactococcus lactis* expressing FGF2, CSF1, and IL-4 (AUP-1602-C) currently tested in a first-human trial (NCT04281992), and the herein investigated ILP100-Topical, *L. reuteri* R2LC expressing CXCL12.^11^^,^^21^ In addition to the onsite bacterial production, the lactic acid produced by *L. reuteri* R2LC was demonstrated to reduce CXCL12 degradation within the wound, and thereby further boosting the CXCL12-induced tissue restorative functions of macrophages.^11^ Accelerated healing of wounds by ILP100-Topical was also confirmed in minipigs.^12^

For new modalities, trial design capturing drug-specific characteristics are vital for continued clinical development. The present trial was designed to allow independent evaluations of wounds, reduce the number of individuals and overcome interindividual variability by having wounds treated with ILP100-Topical, placebo and saline in the same participant. The individuals were closely monitored using an extensive set of safety and tolerability assessments, and all wounds were imaged for subsequent off-site, unbiased, high-resolution, and traceable analyses, in addition to the on-site assessments. Notably, no clinically significant deviations from baseline were detected when safety and tolerability were assessed, and no serious AEs were recorded. Treatment of acute wounds with ILP100-Topical was therefore demonstrated to be both safe and well-tolerated at all timepoints and doses tested.

Complete wound healing is the regulatory endpoint considered to be the most clinically meaningful by FDA. In this study, wound healing was assessed by blinded and fully traceable, off-site analyses of high-resolution wound images. All three IEs found that a higher proportion of wounds treated with the highest dose of ILP100-Topcial were healed from Day 19. Further, the time to first registration of complete healing was shortened by 10 days following repeated ILP100-Topical treatment with the highest dose. These results are indeed clinically very relevant given that 1–2 days of accelerated healing in acute wounds or healing of 10–15% more non-healing ulcers in patients with diabetes compared to standard of care is regarded as clinically meaningful and suffice the requirements for marketing authorisation by regulatory authorities.^22^

To increase the probability of capturing AEs and effects on wound healing in this first-in-human trial, we combined the clinical assessment of the wounds with objective, explorative techniques measuring local blood perfusion and scar formation. These different techniques together generated more than 100 000 data points analysed in a blinded manner. While the small size of the scars precluded comparisons between treatments, a transient and dose-dependent hyperaemia around the ILP100-Topical-treated wounds were detected at early time points. Together with the observed accelerated healing and limited number of transient inflammation-related AEs, this likely reflects biologic effects of the treatment, rather than inflammatory response to bacteria. Thus, the obtained results support continued clinical development of ILP100-Topical for the treatment of difficult-to-heal skin wounds in patients. In fact, two phase 2 trials investigating ILP100-Topical as treatment in different wound types is now approved by European and US health authorities.

Limitations of this study include the single-centre design, the rather small number of study individuals and that different investigators were involved in clinical assessments. Changes in the investigator team during MAD cohort 2 might have influenced the wounding procedures and thereby explain inconsistent results compared to other cohorts. In addition, the individuals included in this study were all healthy, non-obese, and under the age of 45, and is thereby not predisposed for these factors associated with impaired or complex wound healing. Hence, while ILP100-Topical in this study show results supporting an accelerated wound healing in otherwise healthy patients (eg in trauma-related wounds), the effect might not be directly translatable to a patient population exhibiting risk factors for delayed wound healing. As a natural next step in the clinical development efficacy is already being investigated in different well-defined patient populations with pathologies linked to impaired wound healing. Strengths include its design allowing large numbers of wounds, minimal bias as wounds treated with active and control treatment in the same individuals reduce the risk for factors influencing wound healing in different treatment groups, as well as high comparability between treatments for tolerability and effects on healing. This is especially important in a First-in-Human study with few study individuals and at the same time allows for a smaller samples size with fewer individuals exposed to an experimental investigational product in early clinical development. The well-being of the study individuals was thoroughly considered, and each study individual was informed about the study procedures and risks for scar formation before giving consent. Only individuals able to fully understand the study information and comply with the protocol procedures were considered for the study. Further, each study individual fulfilled the inclusion criteria and did not present any of the exclusion criteria including conditions associated with abnormal scar formation and other physical risks, but not mental illness risks. The latter was not considered necessary to evaluate specifically, given that the individuals were informed and accepted the study risk and were assessed for their overall eligibility for participation. Another strength is the wound assessments from high-resolution images, which allows blinded, detailed analyses of both tolerability and wound healing. In conclusion, the favourable safety profile together with the clinical and biologic effects on wound healing support continued clinical development of ILP100-Topical for the treatment of complicated, non-healing wounds in patients. In addition, the study demonstrates that genetically modified bacteria is a new modality enabling the use of short-lived proteins, such as CXCL12, as therapeutics.

## Contributors

The authors Emelie Öhnstedt (EÖ), Evelina Vågesjö (EV), Andreas Fasth (AF), Hava Lofton Tomenius (HLT), Pia Dahg (PD), Sofia Jönsson (SJ), Nisha Tyagi (NT), Mikael Åström (MÅ), Zhanar Myktybekova (ZM), Lovisa Ringstad (LR), Margareth Jorvid (MJ), Peter Frank (PF), Per Hedén (PH), Stefan Roos (SR), and Mia Phillipson (MP) contributed as follows. EV, AF, PH, SR, MJ, and MP were responsible for the conceptualisation. Formal analysis and investigation were the responsibility of EÖ, EV, AF, SR, and MP. EÖ, SJ, ZM, NT, LR, PF, and HLT contributed to the methodology. Project administration was performed by PD and AF, and supervision by EV, AF, SR, and MP. Validation and verification of the data were performed by EÖ, AF, and MP. The first draft of the manuscript was written by MP and review and editing was performed by EÖ;, EV, AF, PH, MÅ, and MP.

EÖ, EV, AF, HLT, PD, SJ, NT, MÅ, ZM, LR, MJ, PF, PH, SR, and MP all had access to the dataset and accept responsibility for the decision to submit for publication.

## Data sharing statement

Deidentified participant data will be made available on request for scientific purposes by contacting the corresponding author. Study protocol are available at: https://www.ilyapharma.se/media/1249/situ-safe-ip-ct-001-sap-summary-of-changes.pdf, https://www.ilyapharma.se/media/1248/situ-safe-ip-ct-001-csp-v30-24feb2020.pdf and https://www.ilyapharma.se/media/1247/situ-safe-ip-ct-001-csp-summary-of-changes.pdf.

## Declaration of interests

MP, SR, PF, MJ, PH and EV are shareholders of Ilya Pharma. EV, AF, EÖ, HLT, LR, NT, PF, SJ, and ZM have stocks options in Ilya Pharma. AF, EÖ, EV, HLT, LR, NT, PF, SJ and ZM are employees, part time or full time, of Ilya Pharma AB. MJ, MÅ, and PD are consultants paid by Ilya Pharma AB for their services. MP and SR renumeration for work in the company Board of Directors.

## Appendix A Supplementary data

Supplementary Methods, Figs. S1–S3, and Tables S1–S18

01-IP-CT-001-Application for EudraCT Number

Abstract in Swedish

situ-safe-ip-ct-001-csp-summary-of-changes

situ-safe-ip-ct-001-csp-v30-24feb2020

situ-safe-ip-ct-001-sap-summary-of-changes
