# Supplementary material for: Engineered bacteria to accelerate wound healing: an adaptive, randomised, double-blind, placebo-controlled, first-in-human phase 1 trial
Source: eClinicalMedicine. 2023 May 25;60:102014. doi: 10.1016/j.eclinm.2023.102014 (PMC10220316; doi:10.1016/j.eclinm.2023.102014)
Supplement: Supplementary Methods, Figs. S1–S3, and Tables S1–S18 [file mmc1.pdf]

Supplementary appendix

Table of Contents

Supplementary methods 2
Supplementary figures........ 4
Supplementary tables 6

List of Figures

Supp. Figure 1 Overview of the study design 4
Supp. Figure 2 Blood perfusion analysis........ 5
Supp. Figure 3CXCL12 in wound biopsies in control- or ILP100-Topical-treated groups (SAD). 5

List of Tables

Supp. Table 1 Baseline characteristics and demographics (SAD) 6
Supp. Table 2 Baseline characteristics and demographics (MAD) 7
Supp. Table 3 Overview of adverse events up to 13 months follow-up in the SAD part. 8
Supp. Table 4 Overview of adverse events – up to 13 months follow-up in the MAD part. 8
Supp. Table 5 Inflammation of skin surrounding the wound score by Independent Evaluators...9
Supp. Table 6 Inflammation of wound and wound edge score by Independent Evaluators. 9
Supp. Table 7 Hemorrhage score by Independent Evaluators........ 10
Supp. Table 8 Exudate score by Independent Evaluators. 10
Supp. Table 9 Slough score by Independent Evaluators. 11
Supp. Table 10 Granulation score by Independent Evaluators. 11
Supp. Table 11 Hypergranulation score by Independent Evaluators. 12
Supp. Table 12 Wound bed perfusion analysed using non-invasive imaging Laser Speckle Contrast Analysis (LASCA) at Days 2, 8, and 15 in ILP100-Topical and control wounds (MAD). 12
Supp. Table 13 Biopsies analysed for total CXCL12 levels at 48 hours following single dose administration of ILP100-Topical or placebo in the SAD part. 13
Supp. Table 14 Scar area measured using 3D spectroscopic scanning 2, 4, 7, and 13 months post wound induction in ILP100 and control group (MAD). 13
Supp. Table 15 Intra-scan variability and inter-scan variability of 3D spectroscopic scanning of scars. 14
Supp. Table 16 Scar volume measured using 3D spectroscopic scanning 2, 4, 7, and 13 months post wound induction in ILP100 and control group (MAD). 14
Supp. Table 17 Intra-scan variability and inter-scan variability of 3D spectroscopic scanning of the colouring of wound areas. 15
Supp. Table 18 Scar redness measured using 3D spectroscopic scanning 2, 4, 7, and 13 months post wound induction in ILP100 and control group (MAD). 15

Supplementary methods

Study procedures

The SAD part of the study included 6 visits for treatment and initial follow-up (Day 1 to Day 14). In the MAD part, there were 12 visits for treatment and initial follow-up (Day 1 to Day 32). All individuals are part of a 5-year long-term follow-up. This report includes results from visits up to 6 weeks, and at 3, 6 and 12 months after the last dose was administered, referred to as 2, 4, 7 and 13 months indicating time post wound induction. After the initial screening visit, individuals fulfilling the study criteria were enrolled.

In SAD, separate adhesive transparent film dressings were used to isolate the wounds from each other. In cohort 1 in the MAD the same dressing was applied throughout the treatment period (Day 1 to Day 19). With repeated administrations of IMP occlusive film dressing caused eczema on the skin surrounding the wounds in the majority of study participants. In cohorts 2 and 3 of the MAD part, the dressing was therefore changed and covered with adhesive, transparent film during 48 hours after the first and second IMP application. From Day 3 and onwards, the wounds were treated with IMP and then covered with adhesive, transparent film for 1 hour only. Thereafter the film was removed, the wounds were allowed to air dry and were then be covered with non-occlusive dressing in accordance with standard wound care procedures. Each wound was dressed until healed.

3D spectroscopic scanner evaluation

In order to understand the precision and the limitations of the 3D spectroscopic scanner (Cherry Imaging, Yokneam, Israel), an evaluation of the scanner was performed using scars scanned in the SAD part. The scar volume and area evaluation were performed 2 months post wound induction, where four scars from the same subject were scanned consecutively for four times in order to assess inter-scan variability. To assess intra-scan variability, each scar was measured five times using the Cherry Imaging software. The scars used for this evaluation were very small with areas and volumes ranging from 23-26 mm^2 and 0.7-1.6 mm^3, respectively, and the results are presented in Supplementary table 15. Evaluation of the measurements of the redness of scars was performed in a similar manner where wounds or skin areas with a redness score of 0.1 to 0.9 were measured repeatedly. For the inter-scan variability 10 areas were used. In total, 20 areas per wound were included in the intra-scan variability assessment, and the defined area within the scan was repeatedly measured five times using the Cherry Imaging software. The results are presented in Supplementary table 17.

Study outcomes

CXCL12 levels in human plasma were analysed using ELISA according to the manufacturer's instructions (Human CXCL12/SDF-1a Quantikine ELISA kit and Quantikine Immunoassay Control Group 3, R&D Systems, Minneapolis, MN, USA). To determine the presence of ADAs, human plasma samples were analysed using a GLP-validated electrochemiluminescent immunoassay (ECLIA). Presence of *L. reuteri* R2LC containing the pSIP_CXCL12 plasmid was analysed in faeces, blood samples and swabs of the area surrounding the wounds by bacterial culturing. PCR and sequencing were used in the occurrence of bacterial culture colonies.

In the SAD part, one placebo-treated wound and one ILP100-Topcial-treated wound were biopsied again at 48 hrs post wounding with an 8 mm in diameter biopsy punch. The biopsy was split in two halves, one was used for histology and one for analysis of tissue CXCL12 by ELISA (Quantikine ELISA Human CXCL12 / SDF-1α Immunoassay, R&D Systems, Minneapolis, MN, USA). The tissue saved for histology was paraffin embedded and stained for CXCL12 (NSJ Bioreagents, RQ4559).

The 3D scans of scar area, volume and pigmentation were analysed (Trace software, Cherry Imaging), and validated (Supplementary Methods and Supplementary Tables 1-2). Blood flow was recorded in an area of 5x10 cm around 2 wounds at a time during at least 2 minutes. Reference perfusion was measured in an area remote from the wound (Supplementary Figure 2), whereas wound edge perfusion was measured in the surrounding skin within 5 mm from the wound border. Blood perfusion of the wound edge is reported as delta perfusion units (dPFU; wound edge perfusion subtracted by reference perfusion). In the SAD part of the study, local mechanism of action was assessed by histology of wound biopsies stained for CXCL12 (NSJ Bioreagents, RQ4559), as well as ELISA to measure total local CXCL12 levels in the wound and immediate surrounding tissue.

Statistical analysis

The statistical analyses for safety and clinical efficacy endpoints included all randomised individuals who received at least one dose of the IMP (Full analyses set; FAS).

No formal sample size calculations were performed for this first-in-human study with the primary objective to study safety and tolerability. The duration of the Treatment Period was selected as sufficiently long in order to assess the safety, PK/PD and preliminary efficacy of ILP100-Topical treatment. This is anticipated long enough to provide initial information about a clinical efficacy during treatment and sufficiently long to capture delayed AEs, a delayed onset of action and requirement of maintenance treatment versus a single dose.

Predefined statistical analyses included a mixed linear regression model for analyses of pairwise (left and right arm as well as wound position on the arm) treatment comparison of time to first registered wound healing and McNemar's paired test for proportion of healed wounds at each timepoint. However, at study design and regulatory approval, as well as at database lock, the wounds in the different treatments groups were considered to be most appropriately analysed as independent based on the influence from biologic parameters related wound healing associated with different use of the dominant and non-dominant hands. These parameters include blood circulation, muscle mass, activity, metabolism and structures of underlying muscles and other tissues, as well as mechanical impact on underlying tissues and abrasion of the skin. In the post-hoc analyses, the biologic and clinical effects were analysed using Fisher´s exact test and the Mann-Whitney test for comparing the different treatment groups for proportion healed wounds and average time to first registered healing. Since the IEs did not perform assessments after Day 32, and the Investigators assessed all wounds as healed on Day 61 (2 months) after wound induction, 61 days was imputed as the timepoint for healing if no earlier timepoint was registered for healing or for wounds with missing data. A safety review committee reviewed all safety and tolerability data throughout the treatment phase. Given the primary objective to assess the safety and tolerability, and the hypothesis-testing nature of the biologic and clinical assessments of wound healing, no adjustments for multiplicity was made.

Supplementary figures

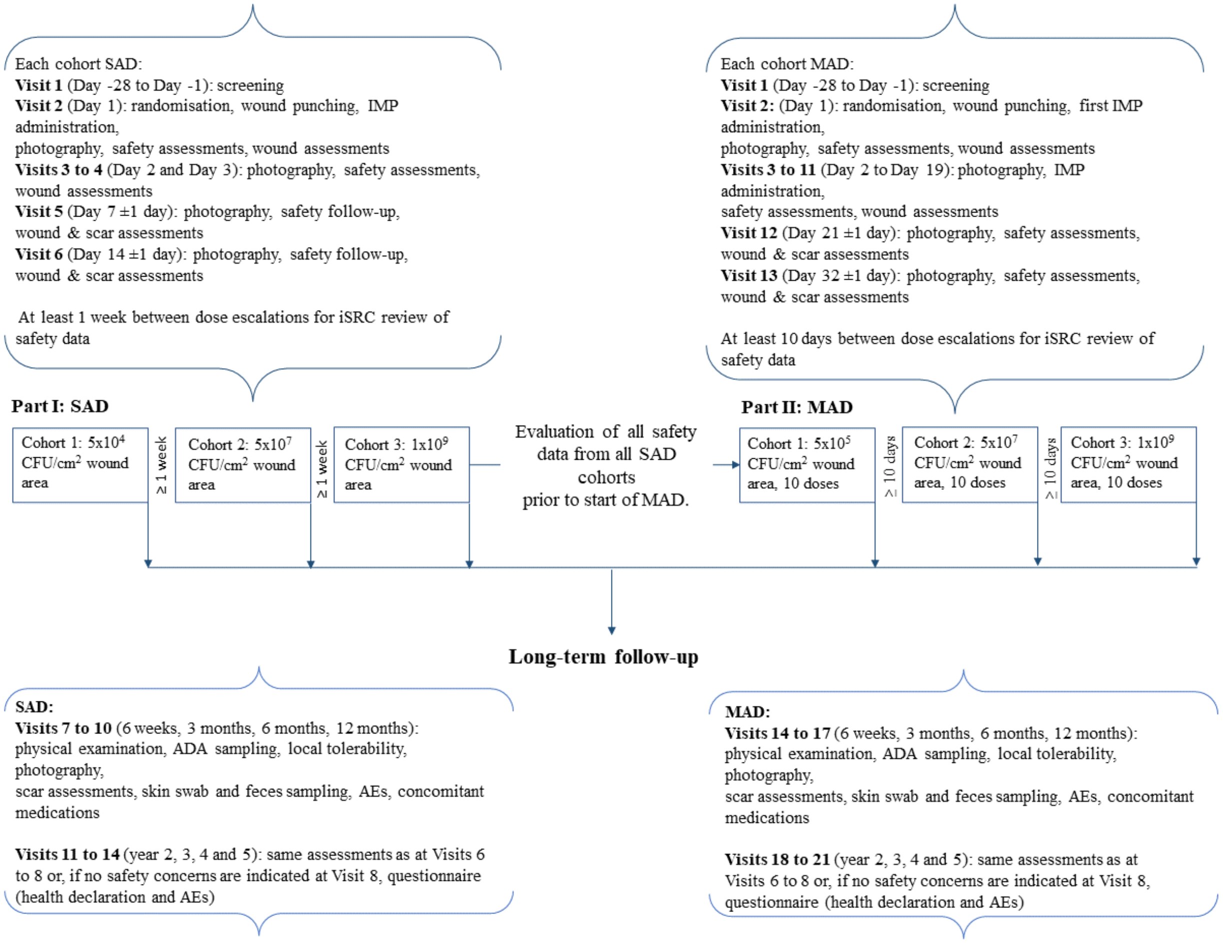

AE=Adverse Event, IE=Independent Evaluator, IMP=Investigational Medicinal Product, iSRC=Independent Safety Review Committee, LASCA=Laser Speckle Contrast Analysis, MAD=Multiple Ascending Dose, SAD=Single Ascending Dose. IMP refers to ILP100, Saline, or Placebo. Safety assessments included ECG, physical examination, vital signs, blood sampling for lab safety, local tolerability, AEs, and CXCL12 anti-drug antibody formation. Wound assessments included CXCL12 blood levels (ELISA), CXCL12 exposure in the wound (immunohistochemistry), microcirculation in the wound (LASCA), as well as evaluation of wound area, wound rupture, and wound healing by IEs (2D photographs). Scar assessments included 3D scanning measurements, as well as evaluation of scar formation by the Investigator and IEs.

Supplementary Figure 1. Overview of the study design

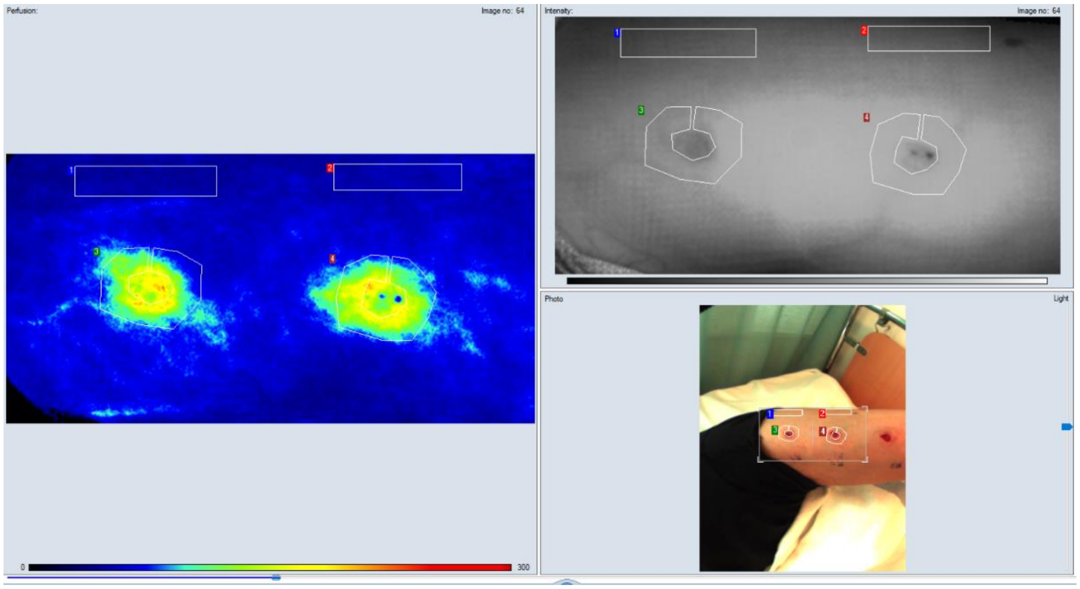

Supplementary Figure 2. Blood perfusion analysis. Blood perfusion of wound edges was measured using LASCA (Laser Speckle Contrast Analysis) at MAD visits and analysed off-site as the difference in blood perfusion between the wound edge (drawn areas surrounding wounds marked green and dark red) and reference areas (top boxes, blue and red region) in each individual.

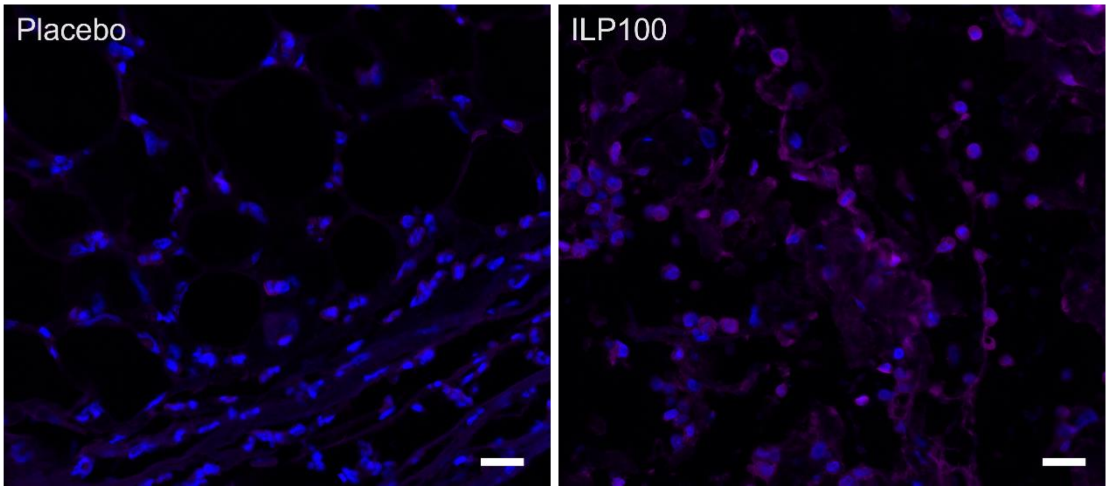

Supplementary Figure 2. CXCL12 in wound biopsies in control- or ILP100-Topical-treated groups (SAD). Representative images of immunofluorescence staining of CXCL12 measured 48 hours post-wound induction in ILP100-Topical or placebo-treated wound biopsies. Blue: nuclei, Magenta: CXCL12, White bar 20 µm.

Supplementary tables

Supplementary Table 1. Baseline characteristics and demographics (SAD)

		Cohort 1 (N=4)	Cohort 2 (N=4)	Cohort 3 (N=4)	Total (N=12)
**Age (years)**	n	4	4	4	12
	Mean (SD)	35.8 (4.6)	33.8 (3.8)	35.8 (4.3)	35.1 (4.0)
	Median (Min, Max)	36.5 (30, 40)	34.0 (30, 37)	36.5 (30, 40)	36.0 (30, 40)
**Body Mass Index (kg/m2)**	n	4	4	4	12
	Mean (SD)	23.8 (3.6)	24.2 (2.9)	25.8 (3.6)	24.6 (3.2)
	Median (Min, Max)	23.6 (21, 28)	24.6 (21, 27)	26.0 (22, 29)	24.6 (21, 29)
**Height (cm)**	n	4	4	4	12
	Mean (SD)	173.5 (6.5)	166.5 (12.7)	187.8 (7.8)	175.9 (12.5)
	Median (Min, Max)	173.5 (167, 180)	166.0 (152, 182)	188.0 (178, 197)	178.0 (152, 197)
**Weight (kg)**	n	4	4	4	12
	Mean (SD)	71.8 (13.5)	67.0 (8.8)	90.5 (8.3)	76.4 (14.2)
	Median (Min, Max)	70.0 (58, 89)	65.0 (59, 79)	88.5 (83, 102)	77.0 (58, 102)
**Sex**	Female	2 (50%)	1 (25%)	0	3 (25%)
	Male	2 (50%)	3 (75%)	4 (100%)	9 (75%)
**Race**	American Indian Or Alaska Native	0	0	0	0
	Asian	1 (25%)	1 (25%)	0	2 (17%)
	Black Or African American	0	0	0	0
	Native Hawaiian Or Other Pacific Islander	0	0	0	0
	White	3 (75%)	3 (75%)	4 (100%)	10 (83%)

Supplementary Table 2. Baseline characteristics and demographics (MAD)

		Cohort 1 (N=8)	Cohort 2 (N=8)	Cohort 3 (N=8)	Total (N=24)
**Age (years)**	n	8	8	8	24
	Mean (SD)	31.9 (5.5)	30.5 (3.6)	36.0 (5.6)	32.8 (5.3)
	Median (Min, Max)	30.5 (26, 42)	31.0 (26, 36)	35.5 (26, 44)	33.0 (26, 44)
**Body Mass Index (kg/m2)**	n	8	8	8	24
	Mean (SD)	24.2 (2.0)	24.6 (2.0)	25.8 (2.1)	24.9 (2.1)
	Median (Min, Max)	24.0 (20, 27)	25.2 (21, 27)	24.9 (24, 29)	24.9 (20, 29)
**Height (cm)**	n	8	8	8	24
	Mean (SD)	175.8 (4.6)	178.9 (9.5)	176.5 (9.3)	177.0 (7.9)
	Median (Min, Max)	176.0 (169, 181)	179.0 (169, 191)	178.5 (158, 189)	178.0 (158, 191)
**Weight (kg)**	n	8	8	8	24
	Mean (SD)	74.8 (7.8)	79.3 (12.4)	80.3 (8.8)	78.1 (9.8)
	Median (Min, Max)	77.0 (58, 84)	81.5 (60, 92)	78.5 (69, 99)	77.5 (58, 99)
**Sex**	Female	5 (63%)	0	2 (25%)	7 (29%)
	Male	3 (38%)	8 (100%)	6 (75%)	17 (71%)
**Race**	Asian	0	1 (13%)	0	1 (4.2%)
	Black Or African American	0	1 (13%)	0	1 (4.2%)
	White	8 (100%)	6 (75%)	8 (100%)	22 (92%)

Supplementary Table 3. Overview of adverse events up to 13 months follow-up in the SAD part. Percentages are based on the number of individuals in the study period included in the full analysis set, n, number of individuals; m, number of events. Study treatment refer to ILP100-Topical, placebo or saline. Percentages are based on the number of individuals included in the full analysis set. Pre-treatment events are not included.

	**Cohort 1 N=4**		**Cohort 2 N=4**		**Cohort 3 N=4**		**Total N=12**	
	**n (%)**	**m**	**n (%)**	**m**	**n (%)**	**m**	**n (%)**	**m**
**Any AE**	4 (100%)	42	4 (100%)	36	4 (100%)	30	12 (100%)	108
**Any SAE**	0	0	0	0	0	0	0	0
**Any AE leading to withdrawal of study drug**	0	0	0	0	0	0	0	0
**Any AE leading to death**	0	0	0	0	0	0	0	0
**Relationship with study treatment**								
Unlikely Related	4 (100%)	19	4 (100%)	13	4 (100%)	9	12 (100%)	41
Possibly Related	4 (100%)	23	4 (100%)	22	4 (100%)	18	12 (100%)	63
Probably Related	0	0	1 (25%)	1	3 (75%)	3	4 (33%)	4
**Severity**								
Mild	4 (100%)	34	4 (100%)	36	4 (100%)	30	12 (100%)	100
Moderate	1 (25%)	8	0	0	0	0	1 (8.3%)	8
Severe	0	0	0	0	0	0	0	0
Life-Threatening	0	0	0	0	0	0	0	0
Death	0	0	0	0	0	0	0	0

Supplementary Table 4. Overview of adverse events up to 13 months follow-up in the MAD part. Study treatment refers to ILP100-Topical, placebo or Saline. n equals number of individuals, m, number of events, SAE, Serious Adverse Events, and AE, Adverse Events. Percentages are based on the number of individuals included in the full analysis set. Pre-treatment events are not included.

	Cohort 1 N=8		Cohort 2 N=8		Cohort 3 N=8		Total N=24	
	**n (%)**	**m**	**n (%)**	**m**	**n (%)**	**m**	**n (%)**	**m**
**Any AE**	8 (100%)	81	8 (100%)	88	8 (100%)	57	24 (100%)	226
**Any SAE**	0	0	0	0	0	0	0	0
**Any AE leading to discontinuation of study treatment**	2 (25%)[a]	28	1 (13%)[b]	1	1 (13%)[c]	1	4 (17%)	30
**Any AE leading to death**	0	0	0	0	0	0	0	0
**Relationship of AE with study treatment**								
Unlikely Related	7 (88%)	23	7 (88%)	30	7 (88%)	25	21 (88%)	78
Possibly Related	8 (100%)	58	8 (100%)	58	8 (100%)	32	24 (100%)	148
Probably Related	0	0	0	0	0	0	0	0
**Severity**								
Mild	8 (100%)	77	8 (100%)	87	8 (100%)	56	24 (100%)	220
Moderate	4 (50%)	4	1 (13%)	1	0	0	5 (21%)	5
Severe	0	0	0	0	1 (13%)	1	1 (4.2%)	1
Life-Threatening	0	0	0	0	0	0	0	0
Death	0	0	0	0	0	0	0	0

a. One study individual discontinued treatment due to wound infection after 4 doses of ILP100-Topical, 4 doses of placebo, and 6 doses of saline. Another study individual discontinued treatment on placebo and saline treated wounds due to wound site eczema after receiving 4 doses.

b. One study individual did not perform day 12 to 19 due to upper respiratory tract infection, received 6 doses of ILP100-Topical, placebo, and saline.

c. One study individual did not perform day 17 due to diarrhea. This study individual received 9 doses of ILP100-Topical, placebo and saline.

Supplementary Table 5. Inflammation of skin surrounding the wound score by Independent Evaluators. Mean tolerability score for inflammation of skin surrounding the wound assessed by the three IEs. Mean ±SEM. n, number of individuals; m, number of wound frames analysed per group. Control group includes saline- or placebo-treated wounds pooled. Mean score comparisons were made for each time point by Mann-Whitney test, * indicates p-value <0.05.

			Inflammation of skin surrounding the wound											
			Pre	Post	2	3	5	8	10	12	15	19	21	32
Cohort 1	ILP100	n,N	8,32	8,32	8,32	8,32	8,32	8,32	8,32	8,32	8,32	8,32	8,32	8,32
		Mean	0.43*	0.66*	0.74	0.66	0.43	0.48	0.55	0.56	0.68	0.42	0.41	0.062
		SEM	0.038	0.072	0.095	0.069	0.046	0.091	0.11	0.10	0.13	0.083	0.13	0.023
	Control	n,N	8,32	8,32	8,32	8,32	8,32	8,32	8,32	8,32	8,32	8,32	8,32	8,32
		Mean	0.56	0.92	0.73	0.51	0.48	0.61	0.40	0.48	0.35	0.64	0.59	0.093
		SEM	0.051	0.078	0.096	0.074	0.045	0.098	0.078	0.075	0.062	0.12	0.13	0.040
Cohort 2	ILP100	n,N	8,32	8,32	8,32	8,32	8,32	8,32	8,32	8,32	8,32	8,32	8,32	8,32
		Mean	0.41	0.68	0.90	0.42	0.48	0.29	0.20	0.10	0.073	0.031	0.062	0.0
		SEM	0.047	0.094	0.12	0.079	0.086	0.047	0.033	0.031	0.029	0.017	0.028	0.0
	Control	n,N	8,32	8,32	8,32	8,32	8,32	8,32	8,32	8,32	8,32	8,32	8,32	8,32
		Mean	0.40	0.67	0.65	0.31	0.31	0.22	0.18	0.18	0.062	0.052	0.062	0.010
		SEM	0.058	0.074	0.052	0.033	0.069	0.032	0.042	0.033	0.023	0.022	0.028	0.010
Cohort 3	ILP100	n,N	8,32	8,32	8,32	8,32	8,32	8,32	8,32	8,32	8,32	8,32	8,32	8,32
		Mean	0.43	0.51	0.91*	0.87	0.40	0.42	0.31	0.26	0.13	0.093	0.073	0.010
		SEM	0.038	0.048	0.085	0.084	0.053	0.060	0.047	0.049	0.042	0.031	0.036	0.010
	Control	n,N	8,32	8,32	8,32	8,32	8,32	8,32	8,32	8,32	8,32	8,32	8,32	8,32
		Mean	0.41	0.57	0.69	0.67	0.40	0.27	0.26	0.20	0.14	0.073	0.073	0.052
		SEM	0.033	0.058	0.080	0.084	0.044	0.046	0.047	0.047	0.033	0.029	0.053	0.052

Supplementary Table 6. Inflammation of wound and wound edge score by Independent Evaluators. Mean tolerability score for inflammation of wound and wound edge assessed by the three IEs. Mean ±SEM. N, number of individuals; m, number of wound frames analysed per group. Control group include saline- or placebo-treated wounds pooled. Mean score comparisons were made for each time point by Mann-Whitney test, * indicates p-value <0.05.

			Inflammation of wound and wound edge											
			Pre	Post	2	3	5	8	10	12	15	19	21	32
Cohort 1	ILP100	n,N	8,32	8,32	8,32	8,32	8,32	8,32	8,32	8,32	8,32	8,32	8,32	8,32
		Mean	1.4	1.4	1.5	1.5	1.0	1.2	1.2	1.5	1.3	0.90	0.73	0.54
		SEM	0.095	0.096	0.10	0.11	0.12	0.14	0.14	0.12	0.14	0.12	0.11	0.065
	Control	n,N	8,32	8,32	8,32	8,32	8,32	8,32	8,32	8,32	8,32	8,32	8,32	8,32
		Mean	1.5	1.6	1.5	1.3	1.1	1.2	1.3	1.4	1.1	0.98	0.94	0.48
		SEM	0.071	0.083	0.11	0.13	0.13	0.16	0.14	0.12	0.12	0.12	0.12	0.070
Cohort 2	ILP100	n,N	8,32	8,32	8,32	8,32	8,32	8,32	8,32	8,32	8,32	8,32	8,32	8,32
		Mean	0.97	1.2	1.4	1.6	2.1*	1.8	1.4	1.1	0.53	0.36	0.40	0.14
		SEM	0.073	0.093	0.12	0.10	0.056	0.069	0.10	0.11	0.10	0.084	0.085	0.075
	Control	n,N	8,32	8,32	8,32	8,32	8,32	8,32	8,32	8,32	8,32	8,32	8,32	8,32
		Mean	1.1	1.3	1.3	1.4	1.9	1.9	1.6	1.2	0.83	0.41	0.35	0.26
		SEM	0.074	0.11	0.11	0.11	0.058	0.065	0.084	0.12	0.11	0.082	0.092	0.088
Cohort 3	ILP100	n,N	8,32	8,32	8,32	8,32	8,32	8,32	8,32	8,32	8,32	8,32	8,32	8,32
		Mean	0.73	1.2	1.3	1.9*	2.1*	1.9	1.6*	1.1	0.68	0.51	0.37	0.052*
		SEM	0.072	0.11	0.11	0.073	0.044	0.074	0.080	0.090	0.081	0.087	0.079	0.043
	Control	n,N	8,32	8,32	8,32	8,32	8,32	8,32	8,32	8,32	8,32	8,32	8,32	8,32
		Mean	0.68	1.1	1.3	1.2	1.8	1.9	1.4	1.1	0.80	0.70	0.54	0.17
		SEM	0.073	0.098	0.11	0.11	0.082	0.078	0.086	0.069	0.086	0.085	0.080	0.076

Supplementary Table 7. **Haemorrhage score by Independent Evaluators**. Mean tolerability score for haemorrhage assessed by the three IEs. Mean ±SEM. n, number of individuals; m, number of wound frames analysed per group. Control group include saline- or placebo-treated wounds pooled. Mean score comparisons were made for each time point by Mann-Whitney test, * indicates p-value <0.05.

			**Haemorrhage**											
			**Pre**	**Post**	**2**	**3**	**5**	**8**	**10**	**12**	**15**	**19**	**21**	**32**
**Cohort 1**	**ILP100**	**n,N**	8,32	8,32	8,32	8,32	8,32	8,32	8,32	8,32	8,32	8,32	8,32	8,32
		**Mean**	1.5	1.2	1.1	0.56	0.33	0.20	0.15	0.11	0.073	0.042	0.031	0.0
		**SEM**	0.10	0.11	0.13	0.084	0.060	0.047	0.062	0.049	0.029	0.020	0.017	0.0
	**Control**	**n,N**	8,32	8,32	8,32	8,32	8,32	8,32	8,32	8,32	8,32	8,32	8,32	8,32
		**Mean**	1.4	1.4	1.1	0.59	0.45	0.20	0.16	0.18	0.15	0.062	0.042	0.010
		**SEM**	0.12	0.13	0.13	0.048	0.055	0.042	0.042	0.056	0.047	0.023	0.020	0.010
**Cohort 2**	**ILP100**	**n,N**	8,32	8,32	8,32	8,32	8,32	8,32	8,32	8,32	8,32	8,32	8,32	8,32
		**Mean**	1.2	1.1	0.38*	0.40	0.39	0.28	0.26	0.16	0.16	0.30	0.18	0.031
		**SEM**	0.099	0.15	0.10	0.081	0.098	0.065	0.080	0.050	0.067	0.11	0.091	0.017
	**Control**	**n,N**	8,32	8,32	8,32	8,32	8,32	8,32	8,32	8,32	8,32	8,32	8,32	8,32
		**Mean**	1.1	0.91	0.76	0.40	0.51	0.40	0.35	0.35	0.23	0.22	0.17	0.063
		**SEM**	0.12	0.094	0.13	0.10	0.12	0.13	0.10	0.11	0.075	0.10	0.091	0.032
**Cohort 3**	**ILP100**	**n,N**	8,32	8,32	8,32	8,32	8,32	8,32	8,32	8,32	8,32	8,32	8,32	8,32
		**Mean**	2.0	0.89	0.48	0.26	0.47	0.30	0.39	0.39	0.32	0.18	0.21	0.021
		**SEM**	0.13	0.12	0.088	0.053	0.099	0.078	0.11	0.072	0.064	0.058	0.070	0.014
	**Control**	**n,N**	8,32	8,32	8,32	8,32	8,32	8,32	8,32	8,32	8,32	8,32	8,32	8,32
		**Mean**	2.0	1.1	0.93	0.53	0.46	0.47	0.42	0.32	0.23	0.17	0.10	0.0
		**SEM**	0.13	0.13	0.17	0.15	0.10	0.098	0.085	0.063	0.048	0.087	0.053	0.0

Supplementary Table 8. Exudate score by Independent Evaluators. Mean tolerability score for exudate assessed by the three IEs. Mean ±SEM. n, number of individuals; m, number of wound frames analysed per group. Control group include saline- or placebo-treated wounds pooled. Mean score comparisons were made for each time point by Mann-Whitney test, * indicates p-value <0.05.

			**Exudate**											
			**Pre**	**Post**	**2**	**3**	**5**	**8**	**10**	**12**	**15**	**19**	**21**	**32**
**Cohort 1**	**ILP100**	**n,N**	8,32	8,32	8,32	8,32	8,32	8,32	8,32	8,32	8,32	8,32	8,32	8,32
		**Mean**	0.25*	0.41	1.9	1.9*	2.1*	1.9*	1.4*	1.1*	1.1*	0.46	0.30	0.0*
		**SEM**	0.030	0.051	0.089	0.11	0.089	0.072	0.14	0.14	0.13	0.12	0.097	0.0
	**Control**	**n,N**	8,32	8,32	8,32	8,32	8,32	8,32	8,32	8,32	8,32	8,32	8,32	8,32
		**Mean**	0.43	0.48	1.7	1.4	0.90	0.96	0.81	0.56	0.39	0.35	0.30	0.083
		**SEM**	0.050	0.052	0.12	0.12	0.10	0.096	0.083	0.090	0.060	0.058	0.055	0.030
**Cohort 2**	**ILP100**	**n,N**	8,32	8,32	8,32	8,32	8,32	8,32	8,32	8,32	8,32	8,32	8,32	8,32
		**Mean**	0.27	0.57*	0.76*	1.6	2.0*	1.8*	1.5*	1.2*	0.91*	0.48	0.21	0.0*
		**SEM**	0.043	0.11	0.11	0.092	0.12	0.095	0.12	0.13	0.12	0.097	0.068	0.0
	**Control**	**n,N**	8,32	8,32	8,32	8,32	8,32	8,32	8,32	8,32	8,32	8,32	8,32	8,32
		**Mean**	0.28	0.81	1.7	1.5	1.2	0.72	0.72	0.54	0.50	0.44	0.29	0.094
		**SEM**	0.034	0.10	0.11	0.10	0.11	0.045	0.076	0.049	0.064	0.075	0.067	0.031
**Cohort 3**	**ILP100**	**n,N**	8,32	8,32	8,32	8,32	8,32	8,32	8,32	8,32	8,32	8,32	8,32	8,32
		**Mean**	0.39*	0.72	1.4	1.7	0.68*	0.77	0.81*	0.82*	0.52	0.33	0.26	0.15*
		**SEM**	0.034	0.094	0.13	0.13	0.068	0.077	0.054	0.10	0.052	0.056	0.055	0.042
	**Control**	**n,N**	8,32	8,32	8,32	8,32	8,32	8,32	8,32	8,32	8,32	8,32	8,32	8,32
		**Mean**	0.28	0.84	1.6	1.5	1.2	0.97	0.66	0.53	0.49	0.34	0.21	0.031
		**SEM**	0.045	0.060	0.13	0.087	0.096	0.081	0.077	0.045	0.050	0.055	0.044	0.023

Supplementary Table 9. Slough score by Independent Evaluators. Mean tolerability score for slough assessed by the three IEs. Mean ±SEM. n, number of individuals; m, number of wound frames analysed per group. Control group include saline- or placebo-treated wounds pooled. Mean score comparisons were made for each time point by Mann-Whitney test, * indicates p-value <0.05.

			Slough											
			Pre	Post	2	3	5	8	10	12	15	19	21	32
Cohort 1	ILP100	n,N	8,32	8,32	8,32	8,32	8,32	8,32	8,32	8,32	8,32	8,32	8,32	8,32
		Mean	0.0	0.016	0.073	0.28	0.31	0.44	0.45	0.33	0.34	0.27	0.12	0.0*
		SEM	0.0	0.016	0.029	0.050	0.042	0.089	0.081	0.045	0.044	0.041	0.033	0.0
	Control	n,N	8,32	8,32	8,32	8,32	8,32	8,32	8,32	8,32	8,32	8,32	8,32	8,32
		Mean	0.0	0.031	0.062	0.19	0.39	0.48	0.51	0.37	0.36	0.27	0.21	0.083
		SEM	0.0	0.017	0.023	0.033	0.054	0.065	0.065	0.044	0.053	0.041	0.042	0.033
Cohort 2	ILP100	n,N	8,32	8,32	8,32	8,32	8,32	8,32	8,32	8,32	8,32	8,32	8,32	8,32
		Mean	0.010	0.094*	0.20	0.24	0.21	0.22	0.31	0.34	0.17	0.042	0.16	0.031
		SEM	0.010	0.034	0.043	0.067	0.068	0.055	0.063	0.069	0.033	0.025	0.040	0.017
	Control	n,N	8,32	8,32	8,32	8,32	8,32	8,32	8,32	8,32	8,32	8,32	8,32	8,32
		Mean	0.0	0.031	0.073	0.16	0.11	0.22	0.20	0.18	0.15	0.11	0.083	0.052
		SEM	0.0	0.023	0.029	0.033	0.032	0.046	0.039	0.040	0.042	0.041	0.026	0.022
Cohort 3	ILP100	n,N	8,32	8,32	8,32	8,32	8,32	8,32	8,32	8,32	8,32	8,32	8,32	8,32
		Mean	0.042	0.073	0.31*	0.40*	0.35	0.52*	0.32	0.27	0.20	0.21	0.073	0.042
		SEM	0.020	0.029	0.072	0.088	0.098	0.11	0.083	0.041	0.029	0.042	0.025	0.020
	Control	n,N	8,32	8,32	8,32	8,32	8,32	8,32	8,32	8,32	8,32	8,32	8,32	8,32
		Mean	0.031	0.010	0.073	0.078	0.22	0.25	0.23	0.18	0.21	0.20	0.14	0.094
		SEM	0.017	0.010	0.029	0.027	0.045	0.067	0.038	0.040	0.033	0.036	0.029	0.027

Supplementary Table 10. Granulation score by Independent Evaluators. Mean tolerability score for granulation assessed by the three IEs. Mean ±SEM. n, number of individuals; m, number of wound frames analysed per group. Control group include saline- or placebo-treated wounds pooled. Mean score comparisons were made for each time point by Mann-Whitney test, * indicates p-value <0.05.

			Granulation											
			Pre	Post	2	3	5	8	10	12	15	19	21	32
Cohort 1	ILP100	n,N	8,32	8,32	8,32	8,32	8,32	8,32	8,32	8,32	8,32	8,32	8,32	8,28
		Mean	0.36	0.33	0.39	0.41	0.51	0.51	0.80	1.2	0.98	0.81	0.50	0.11
		SEM	0.035	0.030	0.040	0.051	0.074	0.083	0.10	0.12	0.14	0.16	0.13	0.074
	Control	n,N	8,32	8,32	8,32	8,32	8,32	8,32	8,32	8,32	8,32	8,32	8,32	8,28
		Mean	0.29	0.40	0.37	0.44	0.43	0.56	0.84	1.1	1.1	0.75	0.62	0.30
		SEM	0.033	0.028	0.038	0.042	0.056	0.086	0.098	0.12	0.14	0.15	0.15	0.12
Cohort 2	ILP100	n,N	8,32	8,32	8,32	8,32	8,32	8,32	8,32	8,32	8,32	8,32	8,31	8,32
		Mean	0.30	0.27	0.44	0.47	0.47	0.62	0.77	0.85	0.58	0.36	0.29	0.078
		SEM	0.023	0.035	0.064	0.057	0.065	0.090	0.10	0.10	0.13	0.12	0.12	0.046
	Control	n,N	8,32	8,32	8,32	8,32	8,32	8,32	8,32	8,32	8,32	8,32	8,28	8,32
		Mean	0.27	0.35	0.37	0.35	0.49	0.67	0.94	0.87	0.49	0.45	0.26	0.11
		SEM	0.028	0.033	0.046	0.057	0.046	0.084	0.11	0.13	0.12	0.13	0.11	0.058
Cohort 3	ILP100	n,N	8,32	8,32	8,32	8,32	8,32	8,32	8,32	8,32	8,32	8,32	8,31	8,32
		Mean	0.16	0.14	0.34	0.30	0.64	0.53	0.73	0.48*	0.23	0.078	0.20	0.094
		SEM	0.030	0.029	0.043	0.054	0.061	0.085	0.091	0.11	0.075	0.064	0.081	0.052
	Control	n,N	8,32	8,32	8,32	8,32	8,32	8,32	8,32	8,32	8,32	8,32	8,31	8,32
		Mean	0.12	0.19	0.34	0.41	0.54	0.65	0.82	0.82	0.35	0.22	0.17	0.0
		SEM	0.029	0.030	0.040	0.054	0.064	0.072	0.10	0.13	0.11	0.092	0.062	0.0

Supplementary Table 11. Hypergranulation score by Independent Evaluators. Mean tolerability score for hypergranulation assessed by the three IEs. Mean ±SEM. n, number of individuals; m, number of wound frames analysed per group. Control group include saline- or placebo-treated wounds pooled. Mean score comparisons were made for each time point by Mann-Whitney test, * indicates p-value <0.05.

			Hypergranulation											
			**Pre**	**Post**	**2**	**3**	**5**	**8**	**10**	**12**	**15**	**19**	**21**	**32**
**Cohort 1**	**ILP100**	**n,N**	8,32	8,32	8,32	8,32	8,32	8,32	8,32	8,32	8,32	8,32	8,32	8,28
		**Mean**	0.0	0.021	0.0	0.0	0.0	0.0	0.010	0.021	0.031	0.052	0.073	0.012
		**SEM**	0.0	0.014	0.0	0.0	0.0	0.0	0.010	0.014	0.023	0.026	0.029	0.012
	**Control**	**n,N**	8,32	8,32	8,32	8,32	8,32	8,32	8,32	8,32	8,32	8,32	8,32	8,28
		**Mean**	0.010	0.0	0.0	0.010	0.0	0.0	0.010	0.031	0.062	0.18	0.11	0.060
		**SEM**	0.010	0.0	0.0	0.010	0.0	0.0	0.010	0.017	0.028	0.083	0.038	0.025
**Cohort 2**	**ILP100**	**n,N**	8,32	8,32	8,32	8,32	8,32	8,32	8,32	8,32	8,32	8,32	8,31	8,32
		**Mean**	0.0	0.0	0.010	0.031	0.021	0.010	0.010	0.010	0.010	0.021	0.086	0.0
		**SEM**	0.0	0.0	0.010	0.017	0.014	0.010	0.010	0.010	0.010	0.014	0.046	0.0
	**Control**	**n,N**	8,32	8,32	8,32	8,32	8,32	8,32	8,32	8,32	8,32	8,32	8,28	8,32
		**Mean**	0.0	0.0	0.010	0.0	0.031	0.010	0.0	0.021	0.010	0.031	0.095	0.0
		**SEM**	0.0	0.0	0.010	0.0	0.017	0.010	0.0	0.014	0.010	0.017	0.054	0.0
**Cohort 3**	**ILP100**	**n,N**	8,32	8,32	8,32	8,32	8,32	8,32	8,32	8,32	8,32	8,32	8,32	8,32
		**Mean**	0.0	0.0	0.010	0.010	0.0	0.010	0.031	0.021	0.073	0.083	0.073	0.0
		**SEM**	0.0	0.0	0.010	0.010	0.0	0.010	0.017	0.014	0.051	0.083	0.073	0.0
	**Control**	**n,N**	8,32	8,32	8,32	8,32	8,32	8,32	8,32	8,32	8,32	8,32	8,32	8,32
		**Mean**	0.0	0.0	0.0	0.0	0.0	0.010	0.11	0.052	0.083	0.016	0.042	0.0
		**SEM**	0.0	0.0	0.0	0.0	0.0	0.010	0.044	0.026	0.064	0.016	0.042	0.0

Supplementary Table 12. Wound bed perfusion analysed using non-invasive imaging Laser Speckle Contrast Analysis (LASCA) at Days 2, 8, and 15 in ILP100-Topical and control wounds (MAD). Delta perfusion units (dPFU) are given and represent perfusion of the wound bed minus the reference perfusion within the same image. Mean ±SEM. n, number of individuals; N, number of wound frames analysed per group. Control group include saline- or placebo-treated wounds pooled. p= p-value by Mann-Whitney test.

**LASCA perfusion imaging**		**Day 2**			**Day 8**			**Day 15**		
	**n, N**	**ILP100**	**Control**	**p**	**ILP100**	**Control**	**p**	**ILP100**	**Control**	**p**
**Cohort 1**	7-8, 26-32	99.7±6.2	99.2±6.5	0.99	127.3±15.0	112.3±14.5	0.39	115.6±15.6	125.7±13.2	0.36
**Cohort 2**	6-8, 24-32	109.3±6.8	101.5±6.0	0.12	171.4±8.5	163.7±11.9	0.54	80.1±7.4	85.5±9.3	0.48
**Cohort 3**	7-8, 28-32	108.4±5.9	92.7±7.4	0.13	163.4±10.6	155.3±7.8	0.30	62.8±6.6	67.0±7.5	0.70
**Cohort 1-3**	21-24,84-96	106.1±3.7	97.6±3.9	0.06	154.8±6.8	144.7±7.1	0.18	89.6±7.6	96.6±7.1	0.23

Supplementary Table 13. Biopsies analysed for total CXCL12 levels at 48 hours following single dose administration of ILP100-Topical or placebo in the SAD part. Values are presented as Mean ±SEM, comparing placebo-treated and ILP100-Topical-treated wounds for each subject in the cohort. n, number of individuals; m, number of wound biopsies analysed.

CXCL12 in wound biopsies at 48 hours normalised to total protein (pg/mg total protein)	n, m	ILP100 (m=4/cohort)	Placebo (m=4/cohort)	Ratio
**Cohort 1**	4, 8	113.7±50.6	109.5±36.5	1.0
**Cohort 2**	4, 8	64.7±9.1	95.8±13.3	0.7
**Cohort 3**	4, 8	85.0±24.1	95.9±26.1	1.0
**Cohort 1-3**	12, 24	87.8±3.5	100.4±3.5	0.9

Supplementary Table 14. Scar area measured using 3D spectroscopic scanning 2, 4, 7, and 13 months post wound induction in ILP100 and control group (MAD). Measurements were made using 3D spectroscopic scanning. Mean ±SEM. n, number of individuals; m, number of wound frames analysed per group, Control group include saline- or placebo-treated wounds pooled. p= p-value by Mann-Whitney's exact test.

Scar area	n, N	2 months			4 months			7 months			13 months		
		ILP100	Control	p	ILP100	Control	p	ILP100	Control	p	ILP100	Control	p
**Cohort 1**	7-8,28-32	32.2±9.2	32.1±9.4	0.92	34.9±7.1	36.7±6.5	0.21	44.4±10.8	45.0±10.9	0.90	51.4±12.8	55.1±12.4	0.14
**Cohort 2**	7-8, 28-31	37.3±8.1	40.8±8.9	0.30	46.8±13.1	45.0±11.9	0.90	48.0±10.2	48.3±8.5	0.66	49.7±13.8	49.1±12.0	0.93
**Cohort 3**	8,31-32	36.6±10.5	34.1±5.8	0.75	39.2±7.3	38.5±9.2	0.79	45.3±8.8	43.7±10.9	0.28	47.6±8.8	44.6±8.9	0.21
**Cohort 1-3**	23-24,88-95	35.4±9.5	35.6±8.9	0.76	40.0±10.5	39.9±9.9	0.62	45.9±10.0	45.6±10.3	0.77	49.5±12.4	49.5±11.9	0.99

Supplementary Table 15. Intra-scan variability and inter-scan variability of 3D spectroscopic scanning of scars. Standard deviation (SD) range and coefficient of variance (CV) range for 5 repeated measurements performed on the scar in the same 3D scan (intra-scan variability) and in 5 different scans taken consecutively of the same scar (inter-scan variability). Mean SD refers to the mean value of all SD measurements in the evaluation.

		Intra-scan variability			Inter-scan variability		
	# scars	SD range	SD mean	CV (%) range	SD range	SD mean	CV (%) range
**Area (mm^2)**	4	0.664-2.574	1.720	3-9	0.562-1.868	1.283	2-7
**Volume (mm^3)**	4	0.037-0.482	0.087	7-61	0.106-0.483	0.252	8-44

Supplementary Table 16. Scar volume measured using 3D spectroscopic scanning 2, 4, 7, and 13 months post wound induction in ILP100 and control group (MAD). Measurements were made using 3D spectroscopic scanning. Mean ±SEM. n, number of individuals; m, number of wound frames analysed per group. Control group include saline- or placebo-treated wounds pooled.

Scar volume	n, N	2 months		4 months		7 months		13 months	
		Control	ILP100	Control	ILP100	Control	ILP100	Control	ILP100
**Cohort 1**	7-8,28-32	1.28±1.23	1.05±0.81	1.98±1.68	1.71±1.40	2.77±2.98	2.88±2.82	1.49±4.62	0.73±1.14
**Cohort 2**	7-8, 28-31	2.92±2.64	1.57±1.79	3.50±3.49	3.80±4.34	2.71±2.70	3.49±3.63	2.30±3.02	3.00±4.86
**Cohort 3**	8,31-32	1.84±1.62	2.47±2.47	2.32±1.93	2.71±1.85	2.11±2.28	2.30±1.50	1.93±2.64	2.06±2.42
**Cohort 1-3**	23-24,88-95	2.00±2.01	1.72±1.92	2.57±2.51	2.70±2.85	2.52±2.64	2.88±2.79	1.91±3.49	1.91±3.25

Supplementary Table 17. **Intra-scan variability and inter-scan variability of 3D spectroscopic scanning of the colouring of wound areas.** Standard deviation (SD) range and coefficient of variance (CV) range for 5 repeated measurements performed on skin areas or wounds in the same 3D scan (intra-scan variability) and in 5 different scans taken consecutively of the same skin area or wound (inter-scan variability). Mean SD refers to the mean value of all SD measurements in the evaluation.

	Intra-scan variability				Inter-scan variability			
	**# areas**	**SD range**	**SD mean**	**CV (%) range**	**# areas**	**SD range**	**SD mean**	**CV (%) range**
**Redness (all)**	20	0.00-0.109	0.049	0-50	10	0.011-0.054	0.034	0-25
**Redness score 0.7-0.9**	14	0.00-0.109	0.059	6-13	8	0.011-0.054	0.036	1-6
**Redness score 0.4-0.6**	3	0.00	0.000	0	-	-	-	-
**Redness score 0.1-0.3**	3	0.000-0.044	0.018	0-25	2	0.011, 0.175	0.028	6, 25%

Supplementary Table 18. Scar redness measured using 3D spectroscopic scanning 2, 4, 7, and 13 months post wound induction in ILP100 and control group (MAD). Measurements were made using 3D spectroscopic scanning. Mean ±SEM. n, number of individuals; m, number of wound frames analysed per group, Control group include saline- or placebo-treated wounds pooled. p= p-value by Mann-Whitney test.

Scar redness	n, N	2 months			4 months			7 months			13 months		
		**Control**	**ILP100**	**p**	**Control**	**ILP100**	**p**	**Control**	**ILP100**	**p**	**Control**	**ILP100**	**p**
**Cohort 1**	7-8, 28-32	0.71±0.019	0.77±0.024	0.075	0.72±0.019	0.70±0.026	0.86	0.54±0.017	0.52±0.027	0.32	0.46±0.041	0.47±0.047	0.49
**Cohort 2**	7-8, 28-31	0.62±0.017	0.64±0.020	0.66	0.62±0.024	0.60±0.023	0.46	0.47±0.036	0.50±0.026	0.98	0.26±0.037	0.23±0.038	0.59
**Cohort 3**	8, 31-32	0.63±0.022	0.65±0.030	0.43	0.61±0.028	0.61±0.024	0.99	0.40±0.033	0.37±0.047	0.63	0.25±0.039	0.17±0.049	0.24
**Cohort 1-3**	23-24, 87-95	0.66±0.012	0.68±0.016	0.16	0.65±0.015	0.64±0.015	0.56	0.47±0.018	0.46±0.021	0.53	0.32±0.024	0.28±0.029	0.53
