# Supplementary material for: Engineered bacteria to accelerate wound healing: an adaptive, randomised, double-blind, placebo-controlled, first-in-human phase 1 trial
Source: eClinicalMedicine. 2023 May 25;60:102014. doi: 10.1016/j.eclinm.2023.102014 (PMC10220316; doi:10.1016/j.eclinm.2023.102014)
Supplement: 01-IP-CT-001-Application for EudraCT Number [file mmc2.pdf]

Från: noreply@eudract.ema.europa.eu
Ämne: Application for EudraCT Number
Datum: 29 januari 2019 10:01
Till: marit.wangheim@ctc-ab.se

N

The EudraCT number 2019-000680-24 has been issued for your Sponsor's Protocol Code Number IP-CT-001.

THIS IS AN AUTOMATED EMAIL - PLEASE DO NOT REPLY AS EMAILS RECEIVED AT THIS ADDRESS WILL BE AUTOMATICALLY DELETED.
