# Supplementary material for: Engineered bacteria to accelerate wound healing: an adaptive, randomised, double-blind, placebo-controlled, first-in-human phase 1 trial
Source: eClinicalMedicine. 2023 May 25;60:102014. doi: 10.1016/j.eclinm.2023.102014 (PMC10220316; doi:10.1016/j.eclinm.2023.102014)
Supplement: Abstract in Swedish [file mmc3.docx]

*The following translations in Swedish were submitted by the authors and we reproduce them as supplied. They have not been peer reviewed. Our editorial processes have only been applied to the original abstract in English, which should serve as reference for this manuscript.*

**Bakgrund:** Långsam eller komplex sårläkning är ett växande medicinskt problem och det finns få eller inga läkemedel med dokumenterad effekt tillgängliga . I flera kontrollerade prekliniska modeller har CXCL12-uttryckande mjölksyrabakterier, *Limosilactobacillus reuteri* (ILP100-Topical), visats påskynda sårläkning. Nu rapporterars data från den första kliniska studien i människa, den primära målsättningen var att fastställa säkerhet och tolerabilitet för läkemedelskandidaten ILP100-Topical. Sekundära mål inkluderade bedömningar av kliniska och biologiska effekter på sårläkning med traditionellt accepterade metoder samt explorativa och spårbara mätningar.

**Metoder:** SITU-SAFE är en adaptiv, randomiserad, dubbelblind, placebokontrollerad, fas 1-studie (EudraCT 2019-000680-24) bestående av en del där ökande doser gavs vid ett tillfälle till varje deltagare (SAD), samt en del där flera och ökande doser gavs till varje deltagare (MAD). Både delarna av studien bestod av tre doskohorter vardera. Totalt inducerades 240 sår på överarmarna hos 36 friska frivilliga, varav 4 sår (2/arm) i 12 deltagare i SAD-delen, och 8 sår (4/arm) i 24 deltagare i MAD-delen. Såren randomiserades till behandling med placebo, saltlösning eller ILP100-Topical. Studien utfördes på Fas 1-enheten vid Akademiska sjukhuset i Uppsala, Sverige. Den data som presenteras här insamlades mellan den 20 september, 2019 och den 20 oktober, 2021.

**Resultat:** Hos alla individer och doser var behandling med ILP100-Topical säkert och tolererades väl, och systemisk exponering kunde inte påvisas. En analys med kohorterna i MAD-delen sammanlagda visade en signifikant större andel läkta sår (p=0,020) dag 32 vid multidosering av ILP100-Topical jämfört med saltlösning ochplacebo (76% (73/96) respektive 59% (57/96) läkta sår). Dessutom förkortades tiden till första registrerade läkning med i genomsnitt 6 dagar, och med 10 dagar vid högsta dos. ILP100-Topical ökade mängden CXCL12-positiva celler i såren, samt det lokala blodflödet runt såret.

**Tolkning**: Den fördelaktiga säkerhetsprofilen och de observerade gynnsamma effekterna på sårläkning stöder fortsatt klinisk utveckling av ILP100-Topical för behandling av komplicerade och svårläkta sår i patienter.
