# Supplementary material for: Engineered bacteria to accelerate wound healing: an adaptive, randomised, double-blind, placebo-controlled, first-in-human phase 1 trial
Source: eClinicalMedicine. 2023 May 25;60:102014. doi: 10.1016/j.eclinm.2023.102014 (PMC10220316; doi:10.1016/j.eclinm.2023.102014)
Supplement: situ-safe-ip-ct-001-csp-summary-of-changes [file mmc4.pdf]

Summary of changes of the Statistical Analysis Plan in Ilya Pharma

IP-CT-001 study

SAD

For the analyses of data from the first 6 weeks of the SAD part, a statistical analysis plan (SAP) covering the SAD part was finalized on 13JAN2020, prior to code breaking and soft lock. The SAP is provided. Changes in the planned analyses of data collected during the first 6 weeks in the SAD part are summarized in **Table 1.**

For the 12 months analyses of the SAD part, there were no changes to the planned analyses as described in the SAP addendum dated 11NOV2021.

Table 1 Changes in the planned analyses of data collected up to Week 6 in the SAD part

Change or clarification	Rationale
Histopathology and immunohistochemistry analyses were not performed.	Due to incorrect handling of samples by service provider when preparing for histology analysis, the data set was not prepared in time for this interim CSR. Due to this, assessments as normal/abnormal by histopathology was not possible. Immune cell infiltration is assessed but density of CXCL12+cells in dermis close to the wound and CXCL12 levels in the tissue was reported separately.
Relationship between dose of ILP100 and levels of CXCL12 in wound biopsies and blood was not evaluated using the intended correlation analysis.	High variability in CXCL12 levels between individuals, cohorts and wounds treated with active drug and placebo. Correlation analyses were not considered meaningful.
Wound healing dose-response relationship was not evaluated using the intended correlation analysis or by using McNemar's test.	Most evaluators assessed most wounds at most visits as non-healed. Correlation analysis and statistical analysis were not considered meaningful.
Wound area dose response relationship was not evaluated using the intended correlation analysis.	High variability in the measurements by the different Independent Evaluators.
Descriptive analysis of wound area	Post DBL, the Sponsor performed a review of all 2D photographs of the wounds. Following the review, a number of photographs were found to lack the scale reference necessary for correct wound area measurements. Since proper measurements of those wounds could not be done, data were

Change or clarification	Rationale
	summarized based on evaluable photographs. All measurements were however listed

MAD

For the analyses of data from the first 6 weeks of the MAD part, SAP covering the MAD part was finalized on 24FEB2020, prior to soft lock and code breaking. The SAP is provided. Changes to the planned analyses and the timing of these are summarized in **Table 2.**

For the 12 months analyses of the MAD part, according to SAP addendum dated 11NOV2021, wound rupture and scar formation normal/abnormal assessment was to be tested for differences between saline- and placebo-treated wounds using Fisher's exact test. If no statistically significant difference was seen between the saline- and placebo-treated wounds, they were to be combined into one control group and tested against ILP100-treated wounds.

Since the Investigator assessed scar tissue formation as normal for all healed wounds at all timepoints no formal statistical testing was performed. As only three events of wound rupture were reported for two wounds in one subject, statistical testing between treatment arms and between men and women was omitted.

Table 2 Changes in the planned analyses of data collected up to Week 6 in the MAD part

Change or clarification	Rationale
No descriptive statistics of ADA.	No ADAs towards recombinant CXCL12-ɑ were detected. Data are provided by subject.
No descriptive statistics of wound rupture data.	Due to the rare occurrence of wound rupture no summaries were performed. Data are listed by subject, cohort and treatment.
Additional statistical summaries and analyses:<br>• Statistical summaries of local tolerability variables using mean score and percent of wounds with grades 2 or 3 and increased score compared to baseline by Investigators and by pooling Independent Evaluators' assessment (mean score of the three Independent Evaluators). Each variable is also presented for ILP100, placebo and saline in each	To complement planned tables with aggregated data on data for mean scores and grade 2 or 3 and increased score compared to baseline was added to better display any changes in tolerability over time and to identify any potential safety issues based on the overall Independent Evaluators' assessments as one method to identify worst case.

Change or clarification	Rationale
cohort. For pooled Independent Evaluators' assessments, tolerability variables were assessed as grade 2 or 3 when at least one of the three Independent Evaluators rated them as such.	
• Statistical analysis using Fisher´s exact test of wound healing assessed by the Investigator and the Independent Evaluators using pooled data across all wounds between ILP100, placebo and saline-treated wounds, and between ILP100 and placebo and saline combined (control). The analyses were performed using StatXact Version 11.1.0 (Cytel Inc.).	The predefined statistical method (McNemar test) for paired analyses of frequencies of wound healing between ILP100-treated wounds and placebo- or saline-treated wounds within the cohorts was performed according to the SAP. After database lock the wounds in the different treatments groups were re-considered to most correctly be analyzed as independent, and the effect on wound healing compared between all wounds in the treatment groups instead of wounds within the same subject. Analyses of wound healing was therefore analyzed using Fisher´s exact test.
• Statistical analysis of time to first registered wound healing as assessed by the Investigators and the Independent Evaluators. Wounds with missing data for timepoint for heling or not judged as healed by the end of the study have been imputed as healed after 61 days. Mixed model, pairwise treatment comparison was performed using SAS Version 9.4 (SAS institute, Cary, NC) and Mann-Whitney test, considering the wounds as independent, was performed with GraphPad Prism 9.1.1.225 (GraphPad Software, LLC).	Time to event analyses was used to compare average time to healing of wounds. Sixty-one days (Visit 14; 6 weeks after last dose) was selected for imputation based on that the Investigator indirectly assessed all wounds as healed at that timepoint as scar assessments were performed for all wounds. As Independent Evaluators did not perform assessments later than Day 32 (Visit 13), also 61 days were imputed if no earlier timepoint was registered.
