# Supplementary material for: Engineered bacteria to accelerate wound healing: an adaptive, randomised, double-blind, placebo-controlled, first-in-human phase 1 trial
Source: eClinicalMedicine. 2023 May 25;60:102014. doi: 10.1016/j.eclinm.2023.102014 (PMC10220316; doi:10.1016/j.eclinm.2023.102014)
Supplement: situ-safe-ip-ct-001-csp-v30-24feb2020 [file mmc5.pdf]

**Clinical Study Protocol**	
EudraCT No.	2019-000680-24
Investigational Medicinal Product	*Lactobacillus reuteri* expressing CXCL12 (activated ILP100-DP)
Study Code	IP-CT-001
Protocol Version and Date	Final Version 3.0 24FEB2020

An adaptive, randomized, double-blind, single-center, placebo-controlled first-in-human study evaluating safety, tolerability and exposure of single and multiple ascending doses of *Lactobacillus* expressing CXCL12 administered topically to experimentally induced skin wounds

**Phase**	I, First-in human
**Indication**	Wound healing
**Test product**	*Lactobacillus reuteri* expressing CXCL12 (activated ILP100-DP)
**Sponsor signatory**	Evelina Vågesjö, Ilya Pharma AB<br>Dag Hammarskjölds väg 30<br>SE-752 37 Uppsala, Sweden
**Principal Investigator**	[redacted]<br>[redacted]<br>Uppsala University Hospital,<br>Entrance 85, 2nd level<br>SE-751 85 Uppsala, Sweden
**Clinical study conduct**	[redacted]<br>Uppsala University Hospital,<br>Entrance 85, 2nd level<br>SE-751 85 Uppsala, Sweden<br>[redacted]
**Clinical study management**	[redacted]

1 STUDY SYNOPSIS

**Study Title**	
An adaptive, randomized, double-blind, single-center, placebo-controlled first-in-human study evaluating safety, tolerability and exposure of single and multiple ascending doses of *Lactobacillus* expressing CXCL12 administered topically to experimentally induced skin wounds	
**Study code**<br>IP-CT-001	**EudraCT No**<br>2019-000680-24
**Study period**<br>Treatment phase: Q3 2019 to Q3 2020 followed by a 5-year long-term follow-up	**Phase of development**<br>I, First-in-human
**Principal Investigator**<br>[redacted]	
**Study design**<br>This is an adaptive, randomized, double-blind single-center, placebo-controlled first-in-human (FIH) study designed to evaluate safety, tolerability, exposure and preliminary efficacy of single and multiple ascending doses of *Lactobacillus reuteri* (*L. reuteri*) expressing CXCL12 administered topically to experimentally induced skin wounds in healthy subjects. The study comprises a single ascending dose (SAD) part, a multiple ascending dose (MAD) part and a 5-year long-term follow-up part.	
**Objectives**<br>**Primary objective**<br>To determine the safety and tolerability of *L. reuteri* expressing CXCL12 (activated ILP100-DP) after topical single and multiple dose administration to experimentally induced skin wounds in healthy subjects<br>**Secondary objectives**<br>• To investigate local and systemic exposure of CXCL12 and *L. reuteri* R2LC<br>• To determine the effect of activated ILP100-DP on CXCL12 levels in the wound (biopsy; SAD part only) and in blood<br>• To compare immunohistochemistry and histopathology from wound biopsies after treatment with activated ILP100-DP or placebo (SAD part only)<br>• To assess the time-course of wound healing<br>• To assess the occurrence of wound rupture<br>• To assess scar tissue formation<br>• To assess propagation of *L. reuteri* R2LC containing the pSIP411 plasmid in the blood, on the skin surrounding the wound and/or in feces	

Exploratory objectives

- To assess wound healing and scar tissue formation using a 3D camera (non-CE marked)
- Assessment of microcirculation around the wounds in early and late phase of healing (MAD part only)
- Wound microbiome analysis (from the used dressings; MAD part only)
- Collection of plasma samples and cell fractions for future analyses based on emerging safety data (MAD part only)

Endpoints

Primary endpoints

- Occurrence and frequency of adverse events (AEs)
- Clinically significant changes in laboratory parameters, vital signs, electrocardiogram (ECG), physical examination findings
- Incidence of local reactions (each wound, 0-3) – assessed by Investigator (direct observation) and independent evaluators (photography)
    - Appearance of wound and wound edge (inflammation)
    - Condition of skin surrounding the wound (inflammation)
    - Hemorrhage
    - Amount of exudate present
    - Presence of slough/necrotic tissue
    - Presence of granulation tissue
    - Hypergranulation
- Incidence of local reactions (each wound; 0-3) – subject assessment
    - Pain
    - Pruritus
- Incidence of wound infections
- Determination of bacterially derived CXCL12 anti-drug antibodies (ADA)

Secondary endpoints

- Exposure measured as levels of CXCL12 in wound biopsies (SAD part) and as levels of CXCL12 and *L. reuteri* R2LC in blood (SAD and MAD part) after dose
- Relationship between dose of activated ILP100-DP and levels of CXCL12 in biopsies (SAD part) and plasma [SAD and MAD parts])
- Histology (SAD part):
    - Overall histopathology evaluation (normal/abnormal wound healing process)
    - Immune cell infiltration close to wound area and CXCL12 (relative amounts)
- Relationship between dose of activated ILP100-DP and the time course of wound healing
- Reduction in wound area and granulation area measurements
- Frequency of wound ruptures
- Overall scar tissue formation (normal/abnormal)
- Vancouver Scar Scale total score
- Presence of *L. reuteri* R2LC containing the pSIP411 plasmid in the blood
- Presence of *L. reuteri* R2LC containing the pSIP411 plasmid on the skin surrounding the wounds

- Presence of *L. reuteri* R2LC containing the pSIP411 plasmid in feces

Exploratory endpoints

- Changes in wound and scar tissue appearance over time as assessed by 3D imaging of wound volume, wound area, wound skin color, scar volume, scar area, scar skin color and evenness/roughness of the scar
- Alterations in microcirculation and skin irritation after treatment with activated ILP100-DP, placebo or saline (MAD part only)
- Alterations to the wound microbiome after treatment with activated ILP100-DP, placebo or saline (MAD part only)
- Potential future analysis of Immunoglobulin G (IgGs) or levels of proteins associated with systemic inflammation or immune response (MAD part only)
- Potential future peripheral blood mononuclear cell (PBMC) analysis (MAD part only).

The results from the exploratory endpoints may not be reported in the clinical study report (CSR).

Number of subjects planned

Approximately 30 subjects will be screened to achieve 12 randomized subjects in the SAD part and approximately 60 subjects will be screened to achieve 24 randomized subjects in the MAD part.

Diagnosis and main eligibility criteria

Healthy male and female subjects aged 25 to 45 years who are willing to comply with the study procedures, including experimental incision of 4 to 8 wounds (diameter 6 mm), 2 per upper inner arm in the SAD and 4 per upper inner arm in the MAD, and who have given written informed consent are considered eligible to participate in the study.

Subjects with a history of any bleeding disorder, including prolonged or habitual bleeding, subjects on blood-thinning medication or subjects with e.g. a tattoo or apparent skin abnormality on the upper inner arms will not be included in the study. Neither will pregnant or lactating females..

Methodology

SAD part

In Part I of the study, single topical doses of the IMP will be administered in 3 sequential cohorts of 4 subjects. For each subject, activated ILP100-DP and placebo will be randomized to experimentally induced wounds on the left and right arm in a 1:1 ratio (i.e. 2 wounds on one arm will be treated with activated ILP100-DP and 2 wounds on the other arm will be treated with placebo as randomized). The proposed dose levels are: $5x10^4$, $5x10^7$ and $1x10^9$ CFU/cm^2 wound area.

Subjects will come for 6 visits to the research clinic for screening, treatment and initial follow-up (Visit 1 to Visit 6). After Visit 6, a 5-year long-term follow-up period (Visit 7 to 14) will be initiated as detailed below.

Treatment phase (Visit 1 to Visit 6)

Screening (Visit 1) will take place from Day -28 to Day -1. At Visit 2, eligible subjects will be admitted to the research clinic on Day 1 for pre-dose safety assessments and full-thickness wound punching with a biopsy punch (6 mm in diameter) on the ventral aspect of the upper arms (2 wounds/arm). The wounds should be separated by approximately 4 cm. One biopsy per subject will be saved for baseline histopathology analysis. Each wound will be numbered using a permanent marker. Prior to wound punching, subjects will be treated with local anesthesia (injected and/or topical) and the area will be cleaned with antiseptics (70% ethanol). The wound punching procedure will be detailed in a separate manual.

A single dose of the IMP will be topically applied to each of the wounds using a pipette; activated ILP100-DP to 2 wounds on one arm and placebo to the other 2 wounds on the other arm as randomized. There should be 1 hour (±10%) between the last wound punch and the start of IMP application to allow for pre-dose study assessments and hemostasis. Two unblinded persons will take part of the administration process, one will perform the application of IMP and the other one will confirm that the correct treatment is administered to the intended wound, i.e. in accordance with the randomization list. The IMP administration will be documented by video recording. Each wound will be dressed separately using an adhesive, transparent film, isolating the wounds from each other. An elastic, tubular bandage will be used on top of the films.

The subjects will be carefully monitored by clinical staff during and after IMP administration and will remain at the research clinic for at least 4 hours after dose. Vital signs and ECG will be checked. There is immediate access to equipment, qualified staff and an ICU in case of an acute emergency. Up to 2 subjects will be dosed on the same day.

The wounds will be photographed in a standardized setting according to instructions specified in a separate manual before treatment on Day 1 and at all subsequent visits to the research clinic. Selected members of the clinical staff will receive photography training prior to handling of the camera equipment. In addition, wounds will be analyzed using a 3D camera.

Subjects will come back to the research clinic on Day 2, Day 3 and Day 7 (Visits 3 to 5) for safety assessments (vital signs, blood sampling for safety laboratory parameters [Day 2], local tolerability, AEs and use of concomitant medications), wound photography and blood sampling for analysis of CXCL12 and *L. reuteri* exposure. In addition, on Day 3, approximately 48 hours after IMP administration, the wound healing status will be assessed, a skin swab will be performed and one wound per arm will be removed by an 8 mm biopsy punch (the same wounds from all subjects) for histology analysis. Prior to biopsy punching, the subjects will be treated with local anesthesia. The residual 2 biopsy wounds will be closed using 1-2 surgical sutures and dressed as appropriate. The other 2 wounds (one per arm) will be left untouched for continued evaluation.

The wounds must not at any visit be cleaned (unless necessary for safety reasons, e.g. due to an infection). The area surrounding the wounds should then be carefully cleaned with water if necessary. At visits when swabbing is performed, the area around the wound should be cleaned after swabbing if necessary. Before the subject leaves the research clinic, the wound should be dressed until healed.

An end-of-treatment phase visit (Visit 6) will take place on Day 14 (±1 day) or after early withdrawal.

Long-term follow-up (Visit 7 to Visit 14)

After Visit 6, a 5-year long-term follow-up period (Visits 7 to 14) will be initiated. During the first year, subjects will visit the research clinic 6 weeks and 3, 6 and 12 months after treatment (Visit 7 to Visit 10). Following Visit 10, subjects will be followed-up once yearly by visits to the research clinic (Visit 11 to Visit 14).

Physical examination, AE and concomitant medication questioning, blood sampling for CXCL12 ADA analysis, photography (standard camera and 3D camera), local tolerability assessment (investigator), assessment of scar tissue formation, skin swab and feces sampling for analysis of presence of *L. reuteri* will be performed.

Subjects will be urged to contact the clinic in between visits, including long-term follow-up visits, in case of e.g. development of any new/recurrent cancer, development of infection, immunogenicity related reactions or local reactions associated with the wound/scar area. If considered relevant by the Investigator, extra visits will be scheduled.

MAD part

Part II of the study will explore multiple ascending dosing of *L. reuteri* expressing CXCL12 (activated ILP100-DP) in 3 sequential cohorts, each of 8 subjects. For each subject in a cohort, activated ILP100-DP, placebo and saline (NaCl) will be randomized to in total 8 wounds, 4 on the

right arm and 4 on the left, in a 4:2:2 ratio (i.e. 4 wounds will be treated with activated ILP100-DP, 2 wounds will be treated with placebo and 2 wounds will be treated with saline as randomized). Activated ILP100-DP will be randomized to one of the arms and placebo and saline to the other arm. The IMPs will be administered on Day 1, Day 2 and Day 3 and then 3 times a week over the course of 3 weeks (in total 10 doses).

The proposed dose levels are $5x10^5$, $5x10^7$ and $1x10^9$ CFU/cm^2 wound area. The doses, dose escalations and the dosing schedule may be adjusted based on emerging knowledge of safety and tolerability data observed in the SAD part of the study.

Subjects will come for 13 visits to the research clinic for screening, treatment and initial follow-up (Visit 1 to Visit 13). After Visit 13, a 5-year long-term follow-up period (Visit 14 to 21) will be initiated as detailed below.

Treatment phase (Visit 1 to Visit 13)

Screening (Visit 1) will take place from Day -28 to Day -1. At Visit 2, eligible subjects will be admitted to the research clinic on Day 1 for pre-dose safety assessments and full-thickness wound punching with a biopsy punch (6 mm in diameter) on the ventral aspect of the upper arms (4 wounds/arm). The wounds should be separated by approximately 4 cm. Each wound will be numbered using a permanent marker (repeated at all post-dose visits). Prior to wound punching, subjects will be treated with local anesthesia (injected and/or topical) and the area will be cleaned with antiseptics (70% ethanol). The wound punching procedure will be detailed in a separate manual.

Subsequent to wound punching, the first dose of the IMP (activated ILP100-DP, placebo and saline) will be topically applied to each of the wounds in accordance with the randomization list. There should be 1 hour (±10%) between the last wound punch and the start of IMP application to allow for pre-dose study assessments and hemostasis. Two unblinded persons will take part of the administration process, one will perform the application of IMP and the other one will confirm that the correct treatment is administered to the intended wound, i.e. in accordance with the randomization list. The IMP administration will be documented by video recording. Each wound will be dressed separately using an adhesive, transparent film, isolating the wounds from each other. An elastic, tubular bandage will be used on top of the films. Following IMP administration on Day 1 and Day 2, the wounds will be covered with adhesive, transparent film around the clock for 2 days and 2 nights (except during wound evaluations and IMP application). From Day 3 and onwards, the wounds will be treated with IMP and covered with adhesive, transparent film for 1 hour after IMP application where after the film will be removed. The wounds will be allowed to air dry and will then be separately covered with a non-occlusive dressing that can be used during showering and protects against contamination of viruses and bacteria.

The subjects will be carefully monitored by clinical staff during and after first dose and will remain at the research clinic for approximately 4 hours after dose. Vital signs and ECG will be checked. There is immediate access to equipment, qualified staff and an ICU in case of an acute emergency. Up to 4 subjects will be dosed on the same day.

The wounds will be photographed in a standardized setting according to instructions specified in a separate manual before treatment on Day 1, pre-treatment at the subsequent dosing visits, and at all follow-up visits to the research clinic. Selected members of the clinical staff will receive photography training prior to handling of the camera equipment. In addition, wounds will be analyzed using a 3D camera.

Following the first dose on Day 1, subjects will come for 10 additional visits between Day 2 and Day 21 (Visits 3 to 12). The last dose will be given on Day 19 (Visit 11). Safety will be assessed by vital signs, safety laboratory parameters, local tolerability, AEs, use of concomitant medications and CXCL12 ADA analysis.

The wounds must not at any visit be cleaned (unless necessary for safety reasons, e.g. due to an infection). The area surrounding the wounds should then be carefully cleaned with water if necessary.

At visits when swabbing is performed, the area around the wound should be cleaned after swabbing if necessary. Before the subject leaves the research clinic, the wound should be dressed until healed.

Blood sampling for analysis of CXCL12 and *L. reuteri* systemic levels and a swab of the area surrounding the wounds and feces sampling for analysis of any presence of *L. reuteri* R2LC colonies containing the pSIP411 plasmid will be performed.

For exploratory purposes, microcirculation assessments will be performed and dressings will be collected.

An end-of-treatment phase visit (Visit 13) will take place on Day 32 (± 1 day) or after early withdrawal.

Long-term follow-up (Visit 14 to Visit 21)

After Visit 13, a 5-year long-term follow-up period (Visit 14 to 21) will be initiated. During the first year, subjects will visit the research clinic 6 weeks and 3, 6 and 12 months after treatment (Visit 14 to Visit 17). Following Visit 17, subjects will be followed-up yearly by visits to the research clinic.

Physical examination, AE and concomitant medication questioning, blood sampling for CXCL12 ADA analysis, photography (standard camera and 3D camera), local tolerability assessment (investigator), assessment of scar tissue formation, skin swab and feces sampling for analysis presence of *L. reuteri* will be performed.

Subjects will be urged to contact the clinic in between visits, including long-term follow-up visits, in case of e.g. development of any new/recurrent cancer, development of infection, immunogenicity related reactions or local reactions associated with the wound/scar area. If considered relevant by the Investigator, extra visits will be scheduled.

Investigational Medicinal Products (IMP), dosage and mode of administration

The active pharmaceutical ingredient (API) is living, genetically modified *L. reuteri* R2LC bacteria transformed with a plasmid, pSIP411 containing the gene for the human chemokine CXCL12-1a inserted behind an inducible promoter, referred to as the drug substance. The drug product, ILP100-DP, is the lyophilized drug substance.

ILP100-DP is a Gene Therapy Medicinal Product (GTMP) type of Advanced Therapy Medicinal Product (ATMP) classified as per Article 2 (1)(a,b) of Regulation (EC) No 1394/2007. The activated ILP100 DP is classified as a low risk microbe that and will hence be handled at biosafety level 1 (BSL-1).

The different doses of ILP100-DP will be prepared by dilutions. Before use, the diluted ILP100-DP will be mixed with the activation peptide SppIP, which induces the expression of CXCL12, to generate the ready-to-use product referred to as **activated ILP100-DP**. Activated ILP100-DP is intended for topical application.

Placebo is the ILP100-DP dilution buffer mixed with the activation peptide SppIP. The concentration of the activation peptide SppIP in the reconstituted products (activated ILP100-DP and placebo) is 100 ng/mL.

Sterile Saline (0.9 % NaCl) will be used as a “**no treatment**” control in the MAD part of the study. This solution will be administered in the same volume as the active treatment and placebo.

The planned dose levels of activated ILP100-DP in the SAD part are: $5x10^4$, $5x10^7$ and $1x10^9$ CFU/cm^2 wound area.

The proposed dose levels of activated ILP100-DP in the MAD part are: $5x10^5$, $5x10^7$ and $1x10^9$ CFU/cm^2 wound area.

Duration of treatment

SAD part: a single topical application of activated ILP100-DP to 2 wounds and placebo to 2 wounds

MAD part: 10 topical applications of activated ILP100-DP to 4 wounds, placebo to 2 wounds and saline to 2 wounds

Duration of subject's involvement in the study

SAD part: Each subject is expected to participate for approximately 42 days (including a 28-day screening period) in the first phase of the SAD part and for an additional 5 years in the long-term follow-up.

MAD part: Each subject is expected to participate for approximately 49 days (including a 28-day screening period) in the first phase of the MAD part and for an additional 5 years in the long-term follow-up.

Safety assessments:

- AEs (including wound infection)
- Clinical laboratory parameters
- Vital signs
- ECG
- Physical examinations
- Local tolerability reactions (assessed by the investigator, the subject and by 3-5 independent evaluators)
- CXCL12 ADA formation

Other assessments:

- CXCL12 exposure in wound (biopsy; SAD part only)
- CXCL12 exposure in blood (ELISA and blood culturing).
- Histology (biopsy; SAD part only)
- Wound healing (assessed by the investigator and by 3-5 independent evaluators, also exploratively assessed using 3D camera)
- Wound area (assessed by 3-5 independent evaluators)
- Wound rupture (assessed by the investigator and by 3-5 independent evaluators)
- Scar tissue formation (Vancouver scar scale) assessed by the investigator, by 3-5 independent evaluators; also exploratively assessed using 3D camera)
- Presence of *L. reuteri* R2LC colonies containing the pSIP411 plasmid on the skin surrounding the wound (skin swab)
- Presence of *L. reuteri* R2LC colonies containing the pSIP411 plasmid in feces (feces sample)

**Statistical methods**<br>No formal sample size calculation has been performed. The proposed sample size is considered sufficient to provide adequate information for the study objectives.<br>A statistical analysis plan (SAP) will be prepared prior to database lock (DBL).<br>Data will be summarized by descriptive statistics as appropriate. All descriptive summaries and statistical analyses will be performed using SAS Version 9.4 or later (SAS Institute, Inc., Cary, NC).
**Study reporting**<br>Interim reports based on unblinded data will be prepared after completion of the 6 weeks visits (Visit 7 [SAD part] and Visit 14 [MAD part] and after completion of the 12 months visits (Visit 10 [SAD part] and Visit 17 [MAD part]; one report with combined 12 months SAD and MAD data). The data for the reports will be produced from exports of the cleaned database but no formal DBL will be performed until after the last subject has performed the last 5-year follow-up assessment. A complete ICH-E3 compliant CSR will be written following the final DBL. Data from the long-term follow-up will be reported in yearly development safety update reports (DSURs).

2 TABLE OF CONTENTS

1 STUDY SYNOPSIS ... 2
2 TABLE OF CONTENTS ... 10
3 LIST OF ABBREVIATIONS AND DEFINITIONS OF TERMS ... 17
4 IMPORTANT MEDICAL PROCEDURES TO BE FOLLOWED BY THE INVESTIGATOR ... 20
4.1 Medical emergencies contacts ... 20
5 INVESTIGATOR AND STUDY ADMINISTRATIVE STRUCTURE ... 20
6 INTRODUCTION ... 22
6.1 Background ... 22
6.1.1 The lactic acid bacteria ... 22
6.1.2 Product characteristics ... 23
6.1.3 Mechanism of action ... 23
6.1.4 Non-clinical pharmacology ... 24
6.1.5 Brief summary of non-clinical findings: ... 25
6.1.6 Non-clinical pharmacokinetics and drug metabolism ... 25
6.1.7 Non-clinical toxicology ... 26
6.1.8 Clinical experience ... 27
6.2 Study rationale ... 27
6.3 Risk/benefit assessment ... 27
7 STUDY OBJECTIVES AND ENDPOINTS ... 30
7.1 Primary objective ... 30
7.1.1 Primary endpoints ... 30
7.2 Secondary objectives ... 31
7.2.1 Secondary endpoints ... 31
7.3 Exploratory objectives ... 33
7.3.1 Exploratory endpoints ... 33
8 STUDY DESIGN ... 34
8.1 Overall study design and schedule of events ... 34
8.1.1 Single Ascending Dose (SAD) ... 36
8.1.2 Multiple Ascending Dose (MAD) ... 41
8.2 Rationale for study design ... 48
8.3 Selection of starting dose and rationale for planned dose escalation ... 48
8.3.1 Selection of starting dose and rationale for dose escalation ... 48

8.3.2 Maximum exposure and dose ... 50
8.4 Dose escalation strategy ... 51
8.4.1 Stopping criteria for dose escalation ... 51
8.4.2 From single to multiple dosing ... 53
8.4.3 Internal safety review committee ... 53
9 STUDY POPULATION ... 54
9.1 Recruitment ... 54
9.2 Screening and enrolment log ... 54
9.3 Number of subjects ... 54
9.4 Inclusion criteria ... 55
9.5 Exclusion criteria ... 55
9.6 Restrictions during the study ... 57
9.6.1 General restrictions ... 57
9.6.2 Prior and concomitant therapy ... 58
9.7 Screen failures ... 59
9.8 Criteria for subject withdrawal ... 59
9.8.1 General withdrawal criteria ... 59
9.8.2 Procedures for discontinuation of a subject from the study ... 59
9.8.2.1 Subject replacement ... 60
9.9 Randomization ... 60
9.10 Blinding ... 61
9.11 Emergency unblinding during the study ... 61
10 TREATMENTS ... 61
10.1 Identity of investigational medicinal products ... 61
10.2 Identity of non-investigational medicinal products ... 62
10.3 Manufacturing, packaging and labelling ... 62
10.4 Conditions for storage ... 63
10.5 Dispensing and accountability ... 63
10.6 Treatment administration and dressing ... 63
10.7 Continuation of treatment with Investigational Medicinal Product ... 64
10.8 Treatment compliance ... 64
10.9 Return and destruction of investigational medicinal products ... 64
11 STUDY ASSESSMENTS ... 65
11.1 Recording of data ... 65

11.2 Procedures to mitigate and monitor environmental spread of gene modified organisms ... 65
11.3 Demographics and other baseline characteristics ... 65
11.3.1 Informed consent ... 65
11.3.2 Eligibility criteria ... 65
11.3.3 Demographic information ... 65
11.3.4 Weight and height ... 65
11.3.5 Medical/surgical history ... 66
11.3.6 Prior and concomitant medication ... 66
11.3.7 HIV and Hepatitis B/C ... 66
11.3.8 Pregnancy test ... 66
11.3.9 Urine drug screen ... 66
11.3.10 Alcohol breath test ... 66
11.3.11 Baseline symptoms ... 66
11.4 Wound punching/biopsy ... 67
11.5 Wound and Scar Photography ... 67
11.6 Dose formulation analysis ... 68
11.7 Safety assessments ... 68
11.7.1 Adverse events ... 68
11.7.1.1 Definition of adverse event ... 68
11.7.1.2 Definition of serious adverse event ... 68
11.7.1.3 Definition of adverse drug reaction ... 69
11.7.1.4 Definition of serious adverse drug reaction ... 69
11.7.1.5 Definition of suspected unexpected serious adverse reaction ... 69
11.7.1.6 Time period and frequency for collecting adverse events ... 69
11.7.1.7 Assessment of severity/intensity ... 69
11.7.1.8 Assessment of causal relationship ... 70
11.7.1.9 Assessment of outcome ... 70
11.7.1.10 Collecting adverse events ... 71
11.7.1.11 Recording adverse events ... 71
11.7.1.12 Reporting of serious adverse events ... 71
11.7.1.13 Reporting of SUSARs to EudraVigilance, local CA and IEC ... 72
11.7.1.14 Treatment and follow-up of adverse events ... 72
11.7.1.15 Procedures in case of pregnancy ... 73

11.7.1.16 Treatment of overdose 73
11.7.2 Physical examination 73
11.7.3 Vital signs 73
11.7.4 Resting 12-lead ECG 73
11.7.5 Laboratory safety assessments 74
11.7.6 Evaluation of local tolerability 75
11.7.6.1 Evaluation of local tolerability by the Investigator and by independent evaluators 75
11.7.6.2 Evaluation of local tolerability by the subjects 75
11.7.7 Wound infections 75
11.7.8 Assessment of CXCL12 anti-drug antibody formation 76
11.8 Assessments related to secondary endpoints 76
11.8.1 Blood sampling for analysis of systemic exposure of CXCL12 in plasma and L. reuteri in blood 76
11.8.1.1 Plasma collection for CXCL12 analysis 76
11.8.1.2 Blood culturing for analysis of L. reuteri in blood 76
11.8.1.3 PBMC analysis 77
11.8.2 Biopsy for analysis of local exposure of CXCL12 in the wound, immunohistochemistry, and histopathology (SAD part only) 77
11.8.3 Assessment of wound healing and wound rupture 77
11.8.4 Wound area measurements 78
11.8.5 Evaluation of scar tissue formation 78
11.8.5.1 Vancouver scar scale 78
11.8.6 Assessment of L. reuteri colonies on the skin surrounding the wounds 78
11.8.7 Assessment of presence of L. reuteri in feces 78
11.9 Assessments related to exploratory endpoints 79
11.9.1 3D Imaging 79
11.9.2 Microcirculation 79
11.9.3 Dressing collection for microbiome analysis 79
11.10 Appropriateness of measurements 79
12 PROCEDURES FOR BIOLOGICAL SAMPLES 80
12.1 Sample collection 80
12.2 Volume of blood 80
12.3 Handling, storage and destruction of laboratory samples 80
12.4 Chain of custody of biological samples 80

12.5 Withdrawal of informed consent for donated biological samples................. 81
13 QUALITY MANAGEMENT, QUALITY ASSURANCE AND QUALITY CONTROL 81
13.1 Critical process, system and data identification 81
13.2 Quality assurance and quality control.. 81
14 ETHICAL AND REGULATORY REQUIREMENTS 82
14.1 Ethical conduct of the study 82
14.2 Ethics and regulatory review.. 82
14.3 Subject information and consent 82
14.4 Subject information card 82
14.5 Subject data protection 83
14.6 Changes to the approved clinical study protocol.. 83
14.7 Audits and inspections 84
14.8 Insurance 84
15 STUDY MANAGEMENT 84
15.1 Training of study site personnel.. 84
15.2 Clinical monitoring.. 84
15.3 Medical Monitoring... 85
15.4 Source data documents 85
15.5 Study agreements.. 86
15.6 Study time table and end of study... 86
15.7 Discontinuation of the study 86
15.8 Reporting and publication 86
15.8.1 Clinical study report 86
15.8.2 Annual safety report 87
15.8.3 Confidentiality and ownership of study data 87
15.8.4 Publication 87
15.9 Archiving 87
16 DATA MANAGEMENT 87
16.1 The web based eCRF.. 88
16.2 The entering of data into the eCRF 88
16.3 The query process.. 88
16.4 Audit trail... 88
16.5 External data.. 89

16.6 Medical coding **89**
16.7 Database lock **89**
17 STATISTICAL METHODS AND DETERMINATION OF SAMPLE SIZE . **89**
17.1 General **90**
17.2 Determination of sample size **90**
17.3 Analysis data sets **90**
17.3.1 Full analysis set 90
17.3.2 Per protocol set 90
17.4 Description of study population **90**
17.4.1 Demographics and baseline characteristics 90
17.4.2 Medical/surgical history and prior/concomitant medication 91
17.4.3 Treatment compliance 91
17.5 Analysis of safety endpoints (primary endpoints) **91**
17.5.1 Adverse events 91
17.5.2 Physical examination 91
17.5.3 Vital signs 91
17.5.4 12-lead ECG 91
17.5.5 Safety laboratory analyses 92
17.5.6 Local tolerability 92
17.5.7 Anti-drug-antibody analysis 92
17.6 Analysis of secondary endpoints **92**
17.6.1 CXCL12 levels in blood and in the wound 92
17.6.2 Histology analysis 93
17.6.3 Wound healing 93
17.6.4 Wound area 93
17.6.5 Scar formation 93
17.6.6 Detection of L. reuteri colonies on skin 93
17.6.7 Detection of L. reuteri colonies in feces 93
17.7 Analysis of exploratory endpoints **94**
17.7.1 Changes in wound and scar appearance by 3D imaging 94
17.7.2 Microcirculation analysis 94
17.7.3 Wound microbiome alterations 94
18 REFERENCES **95**
19 APPENDICES **98**

19.1 Signatures ... 98
19.1.1 Principal investigator statement ... 98
19.1.2 Signature page (approval of the clinical study protocol) ... 99
19.2 Declaration of Helsinki ... 100

List of Tables

Table 8.1-1 Schedule of events, SAD part ... 38
Table 8.1-2 Schedule of events, MAD part ... 44
Table 8.3-1 Overview of intended dosing of ILP100-DP (CFU/mL CFU or ng SppIP/wound, cm^2 of wound surface and per BSA) ... 50
Table 8.3-2 Dose margins between high dose animals of the minipig study and the proposed starting and final doses of the SAD and MAD parts ... 50
Table 8.4-1 Dose escalation and stopping rules ... 52
Table 9.9-1 Example of randomization (MAD) ... 60
Table 11.8-1 Vancouver Scar Scale ... 78

List of Figures

Figure 6.1-1 Schematic overview of the CXCL12 mode of action ... 24
Figure 8.1-1 Overview of the study design ... 35

3 LIST OF ABBREVIATIONS AND DEFINITIONS OF TERMS

Abbreviation or term	Explanation
ADA	Anti-drug antibody
ADL	Activities of daily living
ADR	Adverse drug reaction
AE	Adverse event
ALP	Alkaline phosphatase
ALT	Alanine aminotransferase
API	Active pharmaceutical agent
APTT	Activated Partial Thromboplastin Time
AST	Aspartate aminotransferase
ATC	Anatomical therapeutic chemical
ATMP	Advanced therapy medicinal product
AUC	Area under the plasma concentration time curve
BMI	Body mass index
BSA	Body surface area
BSL-1	Biosafety level 1
CA	Competent authority
CFU	Colony forming unit
CIOMS	Council for International Organizations of Medical Sciences
C_{max}	Maximum plasma concentration
CRM	Clinical research manager
CSP	Clinical study protocol
CSR	Clinical study report
[redacted]	[redacted]
CTCAE	Common terminology criteria for adverse events
[redacted]	[redacted]
DBL	Database lock
DFU	Diabetic foot ulcer
DMP	Data management plan
DSUR	Development safety update report
ECG	Electrocardiogram
eCRF	Electronic case report form
EDC	Electronic data capture
EEA	European Economic Area
EMA	European Medicines Agency
ERA	Environment Risk Analysis
FAS	Full analysis set

Abbreviation or term	Explanation
FIH	First-in-human
FDA	U.S. Food and Drug Administration
GCP	Good clinical practice
GDPR	General Data Protection Regulation
GGT	Gamma-glutamyl transferase
GMM	Genetically modified microorganism
GMO	Genetically modified organism
GMP	Good manufacturing practice
GTMP	Gene therapy medicinal product
Hb	Hemoglobin
HBsAg	Hepatitis B surface antigen
HCV	Hepatitis C virus
HIV	Human immunodeficiency virus
IB	Investigator’s brochure
ICF	Informed consent form
ICH	International conference on harmonization
ICU	Intensive care unit
IEC	Independent ethics committee
IMP	Investigational medicinal product
ISF	Investigator site file
iSRC	Internal safety review committee
IUD	Intrauterine device
IUS	Intrauterine hormone releasing system
LAB	Lactic acid producing bacteria
LASCA	Laser Speckle Contrast Analysis
MAD	Multiple ascending dose
MedDRA	Medical dictionary for regulatory activities
MOA	Mechanism of action
MPA	Medical Products Agency
N	Number
NCI	National Cancer Institute
NIH	National Institute of Health
NSAID	Non-steroidal anti-inflammatory drugs
OTC	Over-the-counter
PBMC	Peripheral blood mononuclear cell
PD	Pharmacodynamic(s)
PII	Personally identifiable information
PK	Pharmacokinetic(s)

Abbreviation or term	Explanation
PK(INR)	Prothrombin Complex International Normalized Ratio
PPS	Per protocol set
PT	Preferred term
QC	Quality control
RBC	Red blood cell(s)
RBM	Risk-based monitoring
RSI	Reference Safety Information
SAD	Single ascending dose
SADR	Serious adverse drug reaction
SAE	Serious adverse event
SAP	Statistical analysis plan
SD	Standard deviation
SDV	Source data verification
SNIF	Summary Notification Information Format
SOC	System organ class
SOP	Standard operating procedures
SUSAR	Suspected unexpected serious adverse reaction
TGF-ß	Transforming growth factor beta
TMF	Trial master file
WBC	White blood cell(s)
WHO	World Health Organization

4 IMPORTANT MEDICAL PROCEDURES TO BE FOLLOWED BY THE INVESTIGATOR

4.1 Medical emergencies contacts

The Principal Investigator is responsible for ensuring that procedures and expertise are available to handle medical emergencies during the study. **A medical emergency usually constitutes a serious adverse event (SAE) and is to be reported as such. Detailed SAE reporting procedures are included in Section 11.7.1.12.**

In the case of a medical emergency, the Investigator may contact the Medical Monitor.

Name	Function in the study	Telephone number and e-mail
[redacted]	[redacted]	[redacted]

5 INVESTIGATOR AND STUDY ADMINISTRATIVE STRUCTURE

Sponsor
Ilya Pharma AB
Dag Hammarskjölds väg 30
SE-752 37 Uppsala

Sponsor's Medical Monitor
[redacted]

Sponsor's CEO
Evelina Vågesjö, PhD
Phone: +46 (0)70 636 64 94
E-mail: evelina.vagesjo@ilyapharma.se

Clinical conduct
[redacted]
Uppsala University Hospital,
Entrance 85, 2nd level
SE-751 85 Uppsala, Sweden

Principal Investigator (also member of the iSRC)
[redacted]

Study management
[redacted]

Clinical Research Manager
[redacted]

Scientific Advisor
[redacted]

Biostatistician

[redacted]

Medical writer

[redacted]

Laboratory

- Safety lab

[redacted]

Laboratory

- Safety lab

[redacted]

Laboratory

- CXCL12 in plasma
- Analysis of colonies following blood culture
- Skin swab and feces analysis

[redacted]

Laboratory

- CXCL12 ADA analysis
- CXCL12 in wound biopsies

[redacted]

Laboratory

- biopsies; CXCL12 immunohistochemistry

[redacted]

IMP manufacturing and packaging

[redacted]

IMP labelling

[redacted]

Electronic data capture (EDC; [redacted]) provider

[redacted]

Signatures are provided in Appendix 19.1.

6 INTRODUCTION

6.1 Background

Wounds, acute and chronic, entail a huge distress for people and the disrupted barrier is an immediate window for microbes. Wound care takes up 2 to 4% of the total health care budget in industrialized countries today (Gottrup, 2004; Guest *et al.*, 2015, Heyer *et al.*, 2016, Sen *et al.*, 2009).

There is a large unmet medical need in treatment of acute post-surgical and chronic wounds. Impaired wound healing is a growing medical problem associated with metabolic diseases and aging. Diabetic foot ulcers (DFUs) are chronic in nature and a common complication in persons with diabetes. These ulcers have a high risk of becoming infected, which ultimately might result in limb amputation. The mortality rate after an amputation is 30% within the first year, which is similar to several cancers (Armstrong *et al.*, 2007). The prevalence of DFUs in patients with diabetes is reported from 9% to up to 18% (Norlund *et al.*, 2001; SBU, 2014). Up to 6 million Europeans and an additional 5 million US residents have diabetes and a DFU (Rathmann and Giani, 2004).

Standard care for chronic ulcers comprises surgical or chemical removal of necrotic tissue, repeated dressing changes, and antibiotics to fight infections (Lipsky *et al.*, 2013; IBPG, 2013). With standard care treatment <25 % of the DFUs heal within 12 weeks (OECD, 2012). As DFUs often get infected, patients may need to be treated with intravenous antibiotics for periods extending to weeks. Such treatment requires hospitalization and significantly increases the risk of spreading antibiotic resistance. However, up to 50% of antibiotic regiments prescribed for these reasons were unnecessary or inappropriate (Wise *et al.*, 1998).

In the clinical trials pipeline, mainly Phase I and II, different drug candidates are being evaluated for active wound care such as cell-based therapies, biologics, siRNAs and specific antibiotics. The best alternatives evaluated in late-stage clinical trials are growth factors e.g. FGF, VEGF and HGF and the approved rhPDGF-BB (Becaplermin) that was withdrawn in Sweden 2012. In the US, the product was removed by the Food and Drug Administration (FDA) in 2018.

Ilya Pharma has developed a candidate drug for improved healing of acute post-surgical and chronic wounds; *Lactobacillus reuteri* (*L. reuteri*) expressing the chemokine CXCL12. The primary target indication with a high unmet medical need is DFUs in diabetes patients.

6.1.1 *The lactic acid bacteria*

L. reuteri is a common, commensal bacterial organism found naturally in the gut of humans and mammals. *L. reuteri* is a gram-positive, non-sporing, catalase negative and devoid of cytochromes (Holzapfel *et al.*, 2001) lactic acid producing bacteria (LAB) that has a history as a food additive for the purpose of improved mouth and gut health and is thus normally identified as a probiotic (Mercenier *et al.*, 2003).

The strain *L. reuteri* R2LC was considered as the best candidate in screening/selection experiments for this project. The strain is a natural, gut commensal in some species (mammals and human) strain that was originally isolated from rats.

6.1.2 *Product characteristics*

The active pharmaceutical ingredient (API) is living, genetically modified *L. reuteri* R2LC bacteria, i.e. a genetically modified organism (GMO) with a plasmid containing the gene for human chemokine CXCL12-1a located behind an inducible promoter, referred to as the drug substance. The expression of CXCL12 is induced by the activation peptide SppIP.

The drug product is the lyophilized drug substance. The ready-to-use drug product (ILP100-DP mixed with the activation peptide SppIP) is referred to as activated ILP100-DP. Activated ILP100-DP is intended as a topical treatment to wounds for accelerated healing.

ILP100-DP is a Gene Therapy Medicinal Product (GTMP) type of Advanced Therapy Medicinal Product (ATMP) classified as per Article 2 (1)(a,b) of Regulation (EC) No 1394/2007.

The activated ILP100 DP is classified as a low risk genetically modified microorganism (GMM) and will be handled at biosafety level 1 (BSL-1) according to AFS 2011:2. BSL-1 organisms are not associated with disease in healthy adult humans (WHO 2004).

For details regarding potential risks, their likelihood and risk management to reduce the likelihood of risks, refer to the Environment Risk Analysis (ERA) and the Summary Notification Information Format (SNIF) for the release of genetically modified organisms other than higher plants in accordance with Article 11 of Directive 2001/18/EC.

6.1.3 *Mechanism of action*

During the inflammation phase of wound healing, immune cells accumulate in response to alarm signals, cytokines, and chemokines released by injured or activated cells (Bianchi, 2007; Gurtner *et al.*, 2008; Orlova *et al.*, 2007; Phillipson and Kubes, 2011; Sozzani *et al.*, 1995). The chemokine CXCL12 (Stromal Cell-Derived Factor 1α) is associated with beneficial effects in models of cutaneous wounds (Badillo *et al.*, 2007; Feng *et al.*, 2014) and binds CXCR4 expressed by immune cells and keratinocytes (Bollag and Hill, 2013). Macrophages and neutrophils represent the major immune cell populations at the wound site, where they are essential for keeping invading microorganisms at bay and also for fueling the healing process by secreting additional chemokines, growth factors, and matrix digesting enzymes. During the course of healing, macrophages shift phenotype toward an anti-inflammatory one and subsequently promote tissue restitution. This shift is induced by macrophage phagocytosis of cell debris and by microenvironmental signals (Boniakowski *et al.*, 2017) such as CXCL12 (Beider *et al.*, 2014; Sanchez-Martin *et al.*, 2011). Immune suppression therapies (Bootun, 2013) and experimental immune cell depletion (Goren *et al.*, 2009; Lucas *et al.*, 2010) delay wound healing, revealing a pivotal immune cell involvement.

Lactobacilli produce lactic acid, which is expected to cause a slight reduction of local pH in the wound following application of activated ILP100-DP. The reduced pH is expected to increase the bioavailability of the delivered CXCL12 due to a pH-dependent inhibition of the CXCL12-degrading peptidase CD26 (Proost et al., 1998).

Wound healing is expected to accelerate based on a bacterial-delivered CXCL12-induced proliferation and phenotype shift of local macrophages in the dermis immediate to the wound. The macrophages are expected to express transforming growth factor beta (TGF-ß) both faster, and in higher concentrations, as compared to in wounds receiving no treatment or treatment with placebo.

An overview of the mechanism of action (MOA) is presented Figure 6.1-1.

Figure 6.1-1   Schematic overview of the CXCL12 mode of action

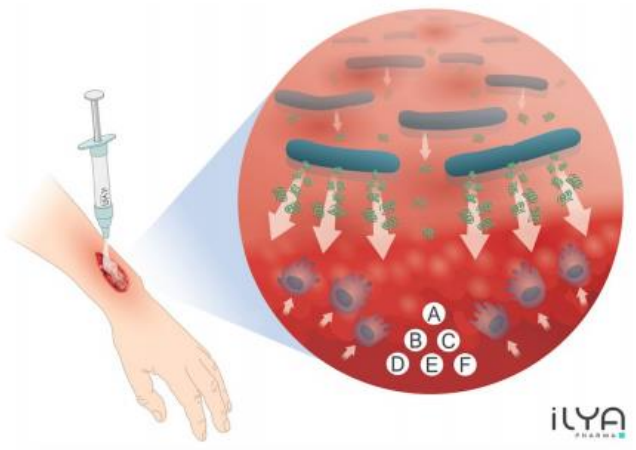

A. Onsite production and delivery of CXCL12 by *L. reuteri* within the wound.

B. Reduction of local pH by the lactic acid produced by *L. reuteri* within the wound.

C. Increased bioavailability of CXCL12 through inactivation of the CXCL12-degrading enzyme CD26 by the local pH reduction.

D. Accumulation of macrophages and increased macrophage production of TGF-β

E. Dermal cell proliferation

F. Strongly accelerated wound closure

6.1.4 *Non-clinical pharmacology*

In summary, the following studies have been performed:

- Multiple analysis of the growth, survival and stability of the *L. reuteri* R2LC with pSIP411 in vitro and in vivo, as well as the bacterial production and release of the chemokine CXCL12 (mouse or human), for details refer to the Investigator's Brochure (IB).
- Multiple studies in mice using different doses, different drug delivery methods and timing of administration of CXCL12-producing *L. reuteri* R2LC or CXCL12 as a recombinant protein to wounds in healthy mice or in mouse models of peripheral ischemia or hyperglycemia, see Section 6.1.5.
- One repeated dose toxicity study in Göttingen minipigs where efficacy parameters were measured along with standard safety parameters and exposure, see Section 6.1.6 and Section 6.1.7.
- A study using biopsies of human skin, a re-epithelialization assay where superficial wounds were created by skin biopsies, which were cultured for 14 days with or without treatment with the *L. reuteri* expressing human CXCL12 (Vågesjö *et al* 2018).

6.1.5 *Brief summary of non-clinical findings:*

- When Lactobacillus expressing CXCL12 is applied topically, the expression of CXCL12 is significant for approximately one hour. The efficacy has been investigated in open dry uncovered wounds in mice and in wounds covered by a film dressing resulting in a moist enclosed wound environment in minipigs. There is no colonization of the wounds by the *lactobacilli* and in uncovered wounds, close to all *lactobacilli* die within one hour. When the wounds are enclosed with a film dressing, some *lactobacilli* can be found on the skin immediate to the wound after 2 to 3 days.
- Administration of *Lactobacillus* expressing CXCL12 to induced wounds in mice daily until healing (ca 8 days) resulted in an acceleration of wound closure as compared to no treatment, treatment with native *Lactobacillus* or recombinant CXCL12.
- Administration of *Lactobacillus* expressing CXCL12 to induced wounds on 15 occasions over 4 weeks in minipigs was associated with an acceleration of wound closure and reduction of scar size.
- *Ex vivo* treatment of human dermal wounds with *Lactobacillus* expressing human CXCL12 for up to 14 days showed increased epithelialization compared to no treatment.
- CXCL12 promotes wound healing by inducing proliferation and phenotype shift of local macrophages in the dermis close to the wound area, resulting in increased expression of TGF-ß. In addition, the reduced local pH as a result of lactic acid produced by the *lactobacilli* inhibits degradation of the delivered CXCL12. Thus, the system with a continuous delivery of CXCL12 in low doses for a short time close to the wound surface and inhibition of inactivation results in a local increase of CXCL12 binding to its receptor CXCR4 on the target cells that induce the phenotype shift.
- The MOA was outlined in multiple studies investigating effects of recombinant- and *Lactobacillus*-derived CXCL12 and included investigations where the target immune cells were blocked. Treatment effects were also studied in mouse models of ischemia and hyperglycemia.
- The systemic exposure of the *Lactobacillus* and *Lactobacillus*-derived CXCL12 have been assessed in mice and in minipigs. No systemic exposure of the *Lactobacillus* (living or DNA from) or *Lactobacillus*-derived CXCL12 could be established in any of the studies despite high doses of activated drug product being administered (see Section 6.1.6 and 6.1.7). Treatment results in increased levels of CXCL12 in the dermis closest to the wound edge and distribution of CXCL12 in wound tissue was detected with immunohistochemistry but no differences between control groups and treatment groups were observed. The biodistribution data obtained reveal no relevant hazard as regards to environmental spread and persistence of *L. reuteri* R2LC.

For further details, refer to the IB and to Section 6.1.6 and 6.1.7 below.

6.1.6 *Non-clinical pharmacokinetics and drug metabolism*

The systemic exposure of the *Lactobacillus* and *Lactobacillus*-derived CXCL12 have been assessed in mice and in minipigs, the latter as a part of a conducted repeated dose toxicity study. No systemic exposure of the *Lactobacillus* or *Lactobacillus*-derived CXCL12 could be

established in any of the studies. Analysis of living bacteria has been conducted by cultivation, being the method with the proven lowest limiting detection level. In mice the wounds were treated with a high dose of $2x10^{10}$ CFU/cm^2 and blood samples were collected via heart puncture three hours post wound induction and treatment. In minipigs, blood sampling was performed at multiple time points over 4 weeks; at the first day, blood samples were taken at baseline (before wound induction), 1 hour post wound induction and 1 hour post wound treatment (2 hours post wound induction). The highest dose used was 1.1 x 10^{10} CFU/cm^2 and six wounds were treated. The highest dose per wound surface area applied in the mouse and minipig study was 20- and 11-fold, respectively, the highest dose proposed to be administered in the SAD part of the FIH study. The total dose of CFU per body surface area (BSA) given to the minipigs is 517-fold the high dose to be given in the SAD.

In the minipig study, dressings were applied on the wound, and thus no shedding of bacteria to surrounding tissue could occur. This will be the case also for the present FIH study.

6.1.7 *Non-clinical toxicology*

One 4-week repeat dose toxicity study including testing of tolerability, sensibility and pharmacokinetics (PK) has been performed in minipigs. Three dose levels of the activated drug product, the activation peptide, wild-type *L. reuteri* were applied topically to full thickness wounds on 15 occasions over 4 weeks.

The mini pig was considered the species of choice for investigating toxicity of dermally applied products (van der Laan et al, 2010; Stricker-Krongrad et al., 2017) since this species allows the testing of doses/concentrations of the product that are many-fold that intended for clinical use and thus enables a reliable toxicity assessment..

The study included in total 6 dose groups with 3 male Göttingen minipigs in each group. There was one control group (Gr 1) receiving no treatment and then there were two “placebo” groups, one receiving the activation peptide (Gr 2) and one receiving wild type R2LC (Gr 3). Gr 4,Gr 5 and Gr 6 were all treated with activated ILP100-DP (R2LC_CXCL12) at $7x10^{10}$ CFU/mL. Gr 4 had 4 wounds treated while Gr 5 had 6 wounds treated. Gr 6 also had 6 wounds treated but the volume applied to these wounds was 500 µL compared to 100 µL in Gr 4 and Gr 5. The wounds were induced by punch biopsy needles where each circular wound had a diameter of 20 mm and an area of 3.14 cm^2.

The design allowed for the assessment of the concentration/dose effect relationship as well as the relationship between number of treated wounds (total dose) and systemic exposure. The testing of 2 concentrations and treatment on 4 to 6 wounds/animal was considered sufficient for this type of product where no systemic availability of either the *Lactobacillus* or *Lactobacillus*-derived CXCL12 was expected.

No treatment-related local or systemic toxicity was identified. Systemic exposure of *Lactobacillus*-derived CXCL12 could not be detected.

All wounds healed normally with earlier onset of reepithelization seen in Group 5 (100 µL/wound) and Group 6 (500 µL/wound). The wound healing process was advanced in all wounds at the end of the study.

There was no treatment related clinical observations and no differences between dose groups were seen on food consumption, body weight, ophthalmoscopy, clinical chemistry or electrocardiogram (ECG) parameters. Examination of wound appearance from photographs

taken during the course of the study showed no treatment-related adverse effects and neither did standard histopathology examinations.

6.1.8 *Clinical experience*

The proposed study is a FIH study and there is no prior clinical experience from administration of ILP100-DP.

6.2 Study rationale

The present study aims to evaluate safety, tolerability, exposure and preliminary efficacy following application of activated ILP100-DP on experimentally induced, full thickness wounds in healthy volunteers.

A FIH study in healthy subjects with multiple induced wounds is proposed as the first study on activated ILP100-DP rather than a Phase I/II study in patients with DFUs based on the novelty of the API and the heterogeneity among 1) patients with DFUs and 2) the microenvironment in the different DFUs that could also be different in different locations.

In addition, with the proposed design, safety can be evaluated by early biopsies of the wounds determining the exact amount of the bacterial-delivered hCXCL12 to the immediate wound tissue.

The study is intended to provide important information to support the design of further studies in patients.

6.3 Risk/benefit assessment

As the healthy volunteers in this study will have no medical benefit from participation, their safety and wellbeing are of outmost importance. The study involves the first administration of *L. reuteri* expressing CXCL12 (activated ILP100-DP) to humans, and there are no previous data on the effects or adverse effects of the drug in humans. It is therefore difficult to make predictions about possible adverse reactions.

Even though the toxicology studies have not indicated any concerns at the dose levels studied, there is still a need for attention to risk mitigation and careful dose escalation. The selection of the starting-dose and dose-escalation steps represents a careful approach to administer the drug for the first time in humans (see Section 8.3). Based on the characteristics of activated ILP100-DP, for which no systemic exposure has been observed, genotoxicity or toxicity studies have not been considered relevant. Hence both males and females will be included in the study.

Subjects will serve as their own control, being concomitantly treated with both activated ILP100-DP and placebo. This approach is considered acceptable since no systemic treatment related effects were found in the non-clinical program, and no *L. reuteri* or bacterial derived-CXCL12 were detected in blood or plasma. The intended exposure in the proposed study is lower than in the repeated dose toxicity study in minipigs, both in terms of dose and number of administrations. The wound surface, as a percent of body surface, is significantly lower in average humans as compared to minipigs.

Immunogenicity is not expected but blood samples for potential CXCL12 anti-drug antibodies (ADAs) will be taken throughout both parts of the study. Formation of autoreactive T-cells is

considered unlikely. One cell fraction will however be saved following the last dose in the MAD part of the study for exploratory peripheral blood mononuclear cell (PBMC) analysis.

In the minipig toxicity study (Section 6.1.7), a small fraction of *L. reuteri* R2LC colonies was detected on the skin surrounding the wounds. Regular skin swabs and feces sampling will therefore be performed in the present study to trace the potential formation of any *L. reuteri* R2LC colonies. All wounds will be covered using a transparent dressing to protect the wounds and to avoid shedding of the bacteria to the surrounding tissue.

No plasmid transfer between bacterial species is expected, for details refer to the ERA and the IB. The plasmid contains an erythromycin resistance marker. To avoid applying a selection pressure for the plasmid, antibiotic treatment with erythromycin, clindamycin or linezolid (the 2 latter due to cross resistance) will not be allowed from 14 days prior to start of IMP administration and should be avoided until at least the 6 weeks follow-up visit in each part (provided that the skin swab and feces samples were negative).

Subjects will remain in the research clinic for at least 4 hours after the administration of the first dose of investigational medicinal product (IMP) and will be closely monitored by medical staff. Visits at the research clinic may be prolonged in case the Investigator finds it medically warranted for safety reasons. In addition, based on emerging safety and tolerability data, additional visits or in-house stays may be added and subjects will be urged to contact the clinic in between visits, including long-term follow-up visits, in case of e.g. development of any new/recurrent cancer, development of infection, immunogenicity related reactions or local reactions associated with the wound/scar area.

Each subject will be provided with a subject information card with information about the subject's participation in a study, see Section 14.4.

Overdosing is not likely to occur since all IMP will be administered by site personnel. In cases of accidental overdose, standard supportive measures should be adopted as required. For further information regarding overdosing, refer to Section 11.7.1.16.

An internal safety review committee (iSRC) will monitor emerging safety and tolerability data over the course of the study and must give a favorable recommendation prior to any dose escalation, see Section 8.4 for stopping criteria for dose escalation and for further information about the iSRC.

The Principal Investigator at the research clinic will ascertain that adequate facilities and procedures are available to handle emergency situations should they occur during the study. The medical staff at [redacted] has extensive experience from early Phase I and FIH studies and adequate procedures are in place to handle unexpected and expected adverse reactions in the study subjects. The FIH research clinic is located adjacent and on the same floor as the Intensive Care Unit (ICU) at the University Hospital in Uppsala. [redacted] has a separate agreement with the ICU for support in case of an emergency and has been regularly inspected by the Swedish Medical Products Agency (MPA) regarding the conduct of FIH studies.

Besides the risks related to the IMP administration as described above, there may also be risks related to the experimental induction of wounds, such as pain, bleeding and bruising. In order to reduce the pain during wound punching, subjects will be treated with local anesthetics (injected and/or topical) prior to the procedure. A brief stinging pain may occur when the local an aesthetic is injected. Anaphylaxis is a possible complication associated with local anesthesia although a true type I hypersensitivity reaction to local anesthesia is very rarely seen. Any anaphylactic reaction is likely to occur prior to the IMP administration.

Bleeding is the most common complication during skin biopsy. Compression for hemostasis will hence be applied if necessary. In case of excessive bleeding (which is rare but may occur), where hemostasis is not achieved by compression, diathermy may be used. For details, refer to Section 11.4. Subjects with bleeding tendency, known bleeding disorders, or subjects who are taking blood-thinning medications such as warfarin will not be included in the study. Also, any vasoactive (constrictor or dilator) medication, prescription or over-the-counter (OTC) drugs that could modulate blood flow is disallowed from 2 weeks prior to screening and during the study. Importantly, this also includes non-steroidal anti-inflammatory drugs (NSAIDs) which are disallowed from 2 weeks prior to screening and during the whole study until Visit 6 of the SAD part and Visit 13 of the MAD part.

Damage to structures such as nerves or vessels are rarely observed complications during skin biopsy.

To minimize the risk for wound infections, the skin will be carefully cleaned with antiseptics (70% ethanol) prior to wound punching and a protective, sterile, non-absorbing dressing will be applied to the wound after IMP administration.

Subjects may experience allergic reactions to the adhesive material of the dressings and will be urged to contact the clinic in case of any local reactions including, but not limited to, itching or other signs of allergy. Extra visits to the clinic will be scheduled if considered necessary by the investigator. Subjects with known history or ongoing allergy/hypersensitivity to the adhesive material of the dressings will not be included in the study.

Scarring with or without hypo- or hyperpigmentation is a common complication seen after healing of the skin biopsy site. Scars can be atrophic or hypertrophic. Very rarely, subjects may develop a keloid over the biopsy site. The sites of wounding (upper inner arms) have been chosen to minimize the exposure of potential scars.

Potential risks associated with the medical devices used in the study e.g. indwelling venous catheters are considered to be low and ethically justifiable, since these are devices that are used in routine medical care. Evaluations and sampling procedures, like blood-pressure measurements using a blood pressure cuff and frequent blood-sampling, may cause transient discomfort but the risk is deemed to be low and ethically justifiable.

The combined safety data from the pre-clinical studies have not revealed any safety issues that would outweigh the expected benefits of the study. The planned study assessments are considered sufficient to meet the scientific and medical goals for the study. It is therefore concluded that the potential benefits from the study will outweigh the potential risks for the treated subjects.

More detailed information about the known and expected benefits and risks of activated ILP100-DP is found in the IB.

7 STUDY OBJECTIVES AND ENDPOINTS

7.1 Primary objective

To determine the safety and tolerability of *L. reuteri* expressing CXCL12 (activated ILP100-DP) after topical single and multiple dose administration to experimentally induced skin wounds in healthy subjects

7.1.1 *Primary endpoints*

Objective	Endpoint	Part	Evaluator	Assessment(s)
To determine the safety and tolerability of *L. reuteri* expressing CXCL12 (activated ILP100-DP) after topical single and multiple dose administration to experimentally induced skin wounds in healthy subjects	Occurrence and frequency of adverse events (AEs)	SAD+ MAD	Investigator	AE reporting and questioning (Section 11.7.1)
	Clinically significant changes in laboratory parameters, vital signs, ECG and physical examination findings	SAD+ MAD	Investigator	Blood sampling for clinical chemistry and hematology (Section 11.7.5),<br>Blood pressure and pulse (Section 11.7.3),<br>12-lead ECG (Section 11.7.4),<br>Physical examination (Section 11.7.2)
	Incidence of local reactions (each wound, 0-3)<br>○ Appearance of wound and wound edge (inflammation)<br>○ Condition of skin surrounding the wound (inflammation)<br>○ Hemorrhage<br>○ Amount of exudate present<br>○ Presence of slough/necrotic tissue<br>○ Presence of granulation tissue<br>○ Hypergranulation	SAD+ MAD	Investigator (direct observation)<br>Independent evaluators (photography)	Local tolerability (Section 11.7.6.1)<br>Photography (Section 11.5)
	Incidence of local reactions (each wound; 0-3)<br>○ Pain<br>○ Pruritus	SAD+ MAD	Subject (direct observation)	Local tolerability (Section 11.7.6.2)
	Incidence of wound infections	SAD+ MAD	Investigator (direct observation)	Wound infection (Section 11.7.7)
	Determination of bacterially derived CXCL12 anti-drug antibodies (ADA)	SAD+ MAD	[redacted]	Blood sampling for ADA analysis (ELISA or AlphaLisa; Section 11.7.8)

SAD: single ascending dose, MAD: multiple ascending dose.

7.2 Secondary objectives

- To investigate local and systemic exposure of CXCL12 and *L. reuteri* R2LC
- To determine the effect of activated ILP100-DP on CXCL12 levels in the wound (biopsy; single ascending dose [SAD] part only) and in blood
- To compare immunohistochemistry and histopathology from wound biopsies after treatment with activated ILP100-DP or placebo (SAD part only)
- To assess the time-course of wound healing
- To assess the occurrence of wound rupture
- To assess scar tissue formation
- To assess propagation of *L. reuteri* R2LC colonies containing the pSIP411 plasmid in the blood, on the skin surrounding the wound and/or in feces

7.2.1 *Secondary endpoints*

Objective	Endpoint	Part	Evaluator	Assessment(s)
To investigate local and systemic exposure of CXCL12 and *L. reuteri* R2LC	Exposure measured as levels of CXCL12 in wound biopsies (SAD part) and as levels of CXCL12 and *L. reuteri* R2LC in blood (SAD and MAD) after dose	SAD	[redacted]	Biopsies for analysis of CXCL12 in the wound, immunohistochemistry (Section 11.8.2)
		SAD+ MAD	[redacted]	Blood sampling for analysis of CXCL12 in plasma (ELISA; Section 11.8.1)
		SAD+ MAD	[redacted]	Blood sampling for analysis of *L. reuteri* R2LC in blood (Blood culturing, Section 11.8.1.2)
To determine the effect of activated ILP100-DP on CXCL12 levels in the wound (biopsy; single ascending dose [SAD] part only) and in blood (SAD and MAD part)	Relationship between dose of activated ILP100-DP and levels of CXCL12 in biopsies (SAD) and plasma (SAD+MAD)	SAD+ MAD		Biopsies for analysis of CXCL12 in the wound (Section 11.8.2)<br>Blood sampling for analysis of CXCL12 in plasma (ELISA; Section 11.8.1)

Objective	Endpoint	Part	Evaluator	Assessment(s)
To compare histopathology from wound biopsies after treatment with activated ILP100-DP or placebo	Overall histopathology evaluation (normal/abnormal wound healing process)	SAD	Evaluated by histopathologist	Biopsies for histopathology (Section 11.8.2)
	Immune cell infiltration close to the wound area and CXCL12 (relative amounts)	SAD	[redacted]	Biopsies for immunohistochemistry (Section 11.8.2)
To assess the time-course of wound healing	Relationship between dose of activated ILP100-DP and the time course of wound healing	SAD+ MAD	Investigator (direct observation)<br>Independent evaluators (photography)	Wound healing definition (Section 11.8.3)
	Reduction in wound area and granulation area measurements	SAD+ MAD	Independent evaluators (photography)	Wound area measurements (Section 11.8.4)
To assess the occurrence of wound rupture	Frequency of wound ruptures	SAD+ MAD	Investigator (direct observation)<br>Independent evaluators (photography)	Wound rupture assessment (Section 11.8.3)
To assess scar tissue formation	Overall scar tissue formation (normal/abnormal)	SAD+ MAD	Investigator (direct observation)<br>Independent evaluators (photography)	Overall assessment of scar tissue formation (Section 11.8.5)
	Vancouver Scar Scale total score on Day 21 and onwards	SAD+ MAD	Investigator (direct observation)<br>Independent evaluators (photography)	Vancouver scar scale (Section 11.8.5.1)

Objective	Endpoint	Part	Evaluator	Assessment(s)
To assess propagation of *L. reuteri* R2LC containing the pSIP411 plasmid in the blood, on the skin surrounding the wound and/or in feces	Presence of *L. reuteri* R2LC containing the pSIP411 plasmid in blood	SAD+ MAD	[redacted]	Blood sampling for analysis of *L. reuteri* R2LC in blood (Blood culturing, Section 11.8.1.2).<br>Note, the blood culture assay is used to answer different objectives regarding exposure and colonization, respectively.
	Presence of *L. reuteri* R2LC containing the pSIP411 plasmid on the skin surrounding the wounds	SAD+ MAD	[redacted]	Skin swab followed by method of culturing conditions specific to detect *L. reuteri* R2LC; PCR and sequencing (Section 11.8.6)
	Presence of *L. reuteri* R2LC containing the pSIP411 plasmid in feces	SAD+ MAD	[redacted]	Feces sampling followed by method of culturing conditions specific to detect *L. reuteri* R2LC, PCR and sequencing (Section 11.8.7)

7.3 Exploratory objectives

- To assess wound healing and scar tissue formation using a 3D camera (non-CE marked)
- Assessment of microcirculation around the wounds in early and late phase of healing (MAD part only)
- Wound microbiome analysis (from the used dressings; MAD part only)
- Collection of plasma samples and cell fractions for future analyses based on emerging safety data (MAD part only)

7.3.1 *Exploratory endpoints*

Objective	Endpoint	Part	Evaluator	Assessment(s)
To assess wound healing and scar tissue formation using a 3D camera (non-CE marked)	Changes in wound and scar tissue appearance over time as assessed by 3D imaging of wound volume, wound area, wound skin color, scar volume, scar area, scar skin color and evenness/roughness of the scar	SAD+ MAD	Trace software	3D imaging (Section 11.9.1)
Assessment of microcirculation around the wounds in early	Alterations in microcirculation and skin irritation after treatment with activated ILP100-DP, placebo or saline	MAD	PIM software.	Laser Speckle Contrast Analysis (Section 11.9.2)

and late phase of healing				
Wound microbiome analysis	Alterations to the wound microbiome after treatment with activated ILP100-DP, placebo or saline	MAD	Illumina or equivalent.	Transfer of the film (used dressings) to and Eppendorf tube or similar (Section 11.9.3)
Collection of plasma samples and cell fractions for future analyses based on emerging safety data	Potential future analysis of Immunoglobulin G (IgGs) or levels of proteins associated with systemic inflammation or immune response.	MAD	TBD	Blood sampling (Section 11.8.1.1)
	Potential future PBMC analysis.	MAD	TBD	Section 11.8.1.3

The results from the exploratory endpoints may not be reported in the clinical study report (CSR).

8 STUDY DESIGN

8.1 Overall study design and schedule of events

This is an adaptive, randomized, double-blind single-center, placebo-controlled FIH study designed to evaluate safety, tolerability, exposure and preliminary efficacy of single and multiple ascending doses of *L. reuteri* expressing CXCL12 (activated ILP100-DP) administered topically to experimentally induced skin wounds in healthy subjects. The study comprises a SAD part, a MAD part, each followed by a 5-year long-term follow-up part, see Section 8.1.1 and Section 8.1.2.

An overview of the study design is shown in Figure 8.1-1.

Figure 8.1-1 Overview of the study design

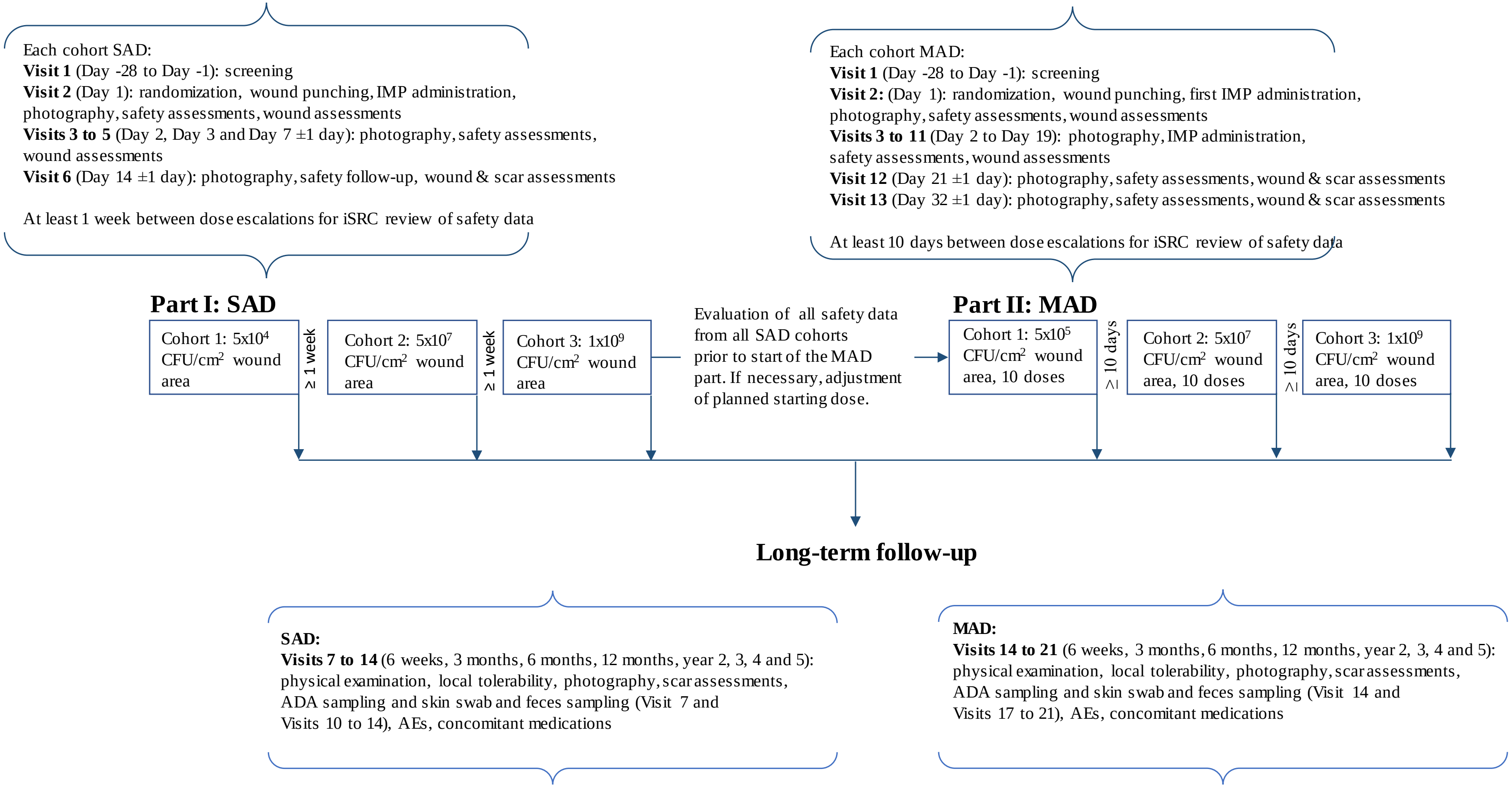

8.1.1 *Single Ascending Dose (SAD)*

In Part I of the study, single topical doses of the IMP will be administered in 3 sequential cohorts of 4 subjects. For each subject, activated ILP100-DP and placebo will be randomized to experimentally induced wounds on the left and right arm in a 1:1 ratio (i.e. 2 wounds on one arm will be treated with activated ILP100-DP and 2 wounds on the other arm will be treated with placebo as randomized).

The proposed dose levels are: $5x10^4$, $5x10^7$ and $1x10^9$ CFU/cm^2 wound area. The rationale for the starting dose and the planned dose escalation is detailed in Section 8.3.

Subjects will come for 6 visits to the research clinic for screening, treatment and initial follow-up (Visit 1 to Visit 6), see Table 8.1-1 for details. After Visit 6, a 5-year long-term follow-up period (Visit 7 to 14) will be initiated as detailed below.

Treatment phase (Visit 1 to Visit 6)

Screening (Visit 1) will take place from Day -28 to Day -1. At Visit 2, eligible subjects will be admitted to the research clinic on Day 1 for pre-dose safety assessments and full-thickness wound punching with a biopsy punch (6 mm in diameter) on the ventral aspect of the upper arms (2 wounds/arm). The wounds should be separated by approximately 4 cm. One biopsy per subject will be saved for baseline histopathology analysis using the procedure described in Section 11.8.2. Each wound will be numbered using a permanent marker. Prior to wound punching, subjects will be treated with local anesthesia (injected and/or topical) and the area will be cleaned with antiseptics (70% ethanol). The wound punching procedure will be detailed in a separate manual.

A single dose of the IMP will be topically applied to each of the wounds using a pipette; activated ILP100-DP to 2 wounds on one arm and placebo to the other 2 wounds on the other arm as randomized. There should be 1 hour (±10%) between the last wound punch and the start of IMP application to allow for pre-dose study assessments and hemostasis. Two unblinded persons will take part of the administration process, one will perform the application of IMP and the other one will confirm that the correct treatment is administered to the intended wound, i.e. in accordance with the randomization list. The IMP administration will be documented by video recording. Each wound will be dressed separately using an adhesive, transparent film, isolating the wounds from each other. An elastic, tubular bandage will be used on top of the films. For details on the wound incision and IMP application processes, refer to Section 11.4 and Section 10.6, respectively.

The subjects will be carefully monitored by clinical staff during and after IMP administration and will remain at the research clinic for at least 4 hours after dose. Vital signs and ECG will be checked as detailed in Table 8.1-1. There is immediate access to equipment, qualified staff and an ICU in case of an acute emergency. Up to 2 subjects will be dosed on the same day.

The wounds will be photographed in a standardized setting according to instructions specified in a separate manual, and briefly summarized in Section 11.5, before treatment on Day 1 and at all subsequent visits to the research clinic. Selected members of the clinical staff will receive photography training prior to handling of the camera equipment. In addition, wounds will be analyzed using a 3D camera as outlined in Section 11.9.1.

Subjects will come back to the research clinic on Day 2, Day 3 and Day 7 (Visits 3 to 5) for safety assessments (vital signs, blood sampling for safety laboratory parameters [Day 2], local tolerability, AEs and use of concomitant medications), wound photography and blood

sampling for analysis of CXCL12 and *L. reuteri* exposure as detailed in Table 8.1-1. In addition, on Day 3, approximately 48 hours after IMP administration, the wound healing status will be assessed, a skin swab will be performed and one wound per arm will be removed by an 8 mm biopsy punch (the same wounds from all subjects) for histology analysis (see Section 11.8.2). Prior to biopsy punching, the subjects will be treated with local anesthesia. The residual 2 biopsy wounds will be closed using 1-2 surgical sutures and dressed as appropriate. The other 2 wounds (one per arm) will be left untouched for continued evaluation.

The wounds must not at any visit be cleaned (unless necessary for safety reasons, e.g. due to an infection). The area surrounding the wounds should then be carefully cleaned with water if necessary. At visits when swabbing is performed, the area around the wound should be cleaned after swabbing if necessary. Before the subject leaves the research clinic, the wound should be dressed until healed.

An end-of-treatment phase visit (Visit 6) will take place on Day 14 (±1 day) or after early withdrawal. For details on assessments, see Table 8.1-1 and Section 11.

Long-term follow-up (Visit 7 to Visit 14)

After Visit 6, a 5-year long-term follow-up period (Visits 7 to 14) will be initiated. During the first year, subjects will visit the research clinic 6 weeks and 3, 6 and 12 months after treatment (Visit 7 to Visit 10). Following Visit 10, subjects will be followed-up once yearly by visits to the research clinic (Visit 11 to Visit 14).

Physical examination, AE and concomitant medication questioning, blood sampling for CXCL12 ADA analysis, photography (standard camera and 3D camera), local tolerability assessment (investigator), assessment of scar tissue formation, skin swab and feces sampling for analysis of presence of *L. reuteri* will be performed at visits outlined in Table 8.1-1.

Subjects will be urged to contact the clinic in between visits, including long-term follow-up visits, in case of e.g. development of any new/recurrent cancer, development of infection, immunogenicity related reactions or local reactions associated with the wound/scar area. If considered relevant by the Investigator, extra visits will be scheduled.

General

Over the course of the study, the investigator will assess local tolerability, wound healing and scar tissue formation by direct observation. The subjects will assess local tolerability (pruritus and pain). Three to five independent experts will assess local tolerability, wound area, wound healing and scar tissue formation by using the wound/scar photographs. For details on timing of assessments, refer to Table 8.1-1.

Before initiating a new cohort, all subjects in the previous cohort must have been treated and Day 3 safety and tolerability data for all treated subjects must have been evaluated by the iSRC, see Section 8.4.3. Once Day 3 safety and tolerability data for the last subject in each cohort has been collected, there will be at least 1week between dose escalations.

Each subject is expected to participate for approximately 42 days (including a 28-day screening period) in the first phase of the SAD part and for an additional 5 years in the long-term follow-up.

The schedule of events is shown in Table 8.1-1. Study assessments are described in Section 11.

Table 8.1-1 Schedule of events, SAD part

	Treatment phase						Long-term follow-up					
	Year 1										Year 2-4	Year 5
	Screening	Randomization & Treatment	Outpatient	Outpatient	Outpatient	End-of-treatment phase[1]	Follow-up	Follow-up	Follow-up	Follow-up	Follow-up	End-of-study
**Visit number**	1	2	3	4	5	6	7	8	9	10	11-13	14
**Assessment / Timepoint**	Day -28 to -1	Day 1	Day 2	Day 3	Day 7 ± 1 day	Day 14 ± 1 day	6 weeks ± 3 days	3 months ± 1 week	6 months ±1 week	12 months ±1 week	Once yearly ±2 weeks	5 years ±1 month
Informed Consent	X											
Inclusion/exclusion criteria	X	X[2,3]										
Demographics	X											
Medical/surgical history	X											
Physical examination	X	X[2]				X	X	X	X	X	X	X
Weight and height	X									X[4]	X[4]	X[4]
Urine analysis (dip stick)	X	X[2]				X						
HIV, Hepatitis B and C	X											
Urine drug screen[5]	X	X[2]				X	X	X	X	X	X	X
Alcohol breath test	X	X[2]				X	X	X	X	X	X	X
Pregnancy test	X[6]	X[2, 6]				X[6]	X					
12-lead safety ECG	X	X[2,7]			X	X						
Vital signs (blood pressure & pulse)	X	X[2, 7]	X	X	X	X						
Body temperature		X[2]										
Hematology, coagulation and clinical chemistry	X	X[2]	X			X						
Randomization		X[2]										
CXCL12 ADA sample		X[8]				X	X			X	X	X
Wound incision by punch biopsy		X[9]										
IMP administration		X										

	**Treatment phase**						**Long-term follow-up**					
	**Year 1**										**Year 2-4**	**Year 5**
	**Screening**	**Randomization & Treatment**	**Outpatient**	**Outpatient**	**Outpatient**	**End-of-treatment phase[1]**	**Follow-up**	**Follow-up**	**Follow-up**	**Follow-up**	**Follow-up**	**End-of-study**
**Visit number**	**1**	**2**	**3**	**4**	**5**	**6**	**7**	**8**	**9**	**10**	**11-13**	**14**
**Assessment / Timepoint**	**Day -28 to -1**	**Day 1**	**Day 2**	**Day 3**	**Day 7 ± 1 day**	**Day 14 ± 1 day**	**6 weeks ± 3 days**	**3 months ± 1 week**	**6 months ±1 week**	**12 months ±1 week**	**Once yearly ±2 weeks**	**5 years ±1 month**
IMP for viable count		X[10]										
Wound/scar photography		X[11]	X	X	X	X	X	X	X	X	X	X
3D camera		X[11]	X	X	X	X	X	X	X	X		
Local tolerability[12]		X[11, 12]	X[12]	X[12]	X[12]	X[12]	X[13]	X[13]	X[13]	X[13]	X[13]	X [13]
Blood sampling for analysis of CXCL12 and *L. Reuteri*		X[14]	X[15]	X[15]	X[15]	X[15]						
Punch biopsy for immunohistochemistry and histopathology				X[16]								
Wound healing[17]				X	X	X						
Wound area[18]		X[19]	X	X	X	X						
Scar tissue formation[20]						X	X	X	X	X	X	X
Skin swab for analysis of *L. reuteri* colonies				X		X	X			X	X	X
Hand out of feces sampling kit	X				X	X			X	X	X	X
Feces sample for analysis of *L. reuteri* colonies		X[8]				X	X			X	X	X
Dressing		X	X	X	X	X[21]						
Baseline symptoms	X											
Adverse events		X[22]	X	X	X	X	X[23]	X[23]	X[23]	X[23]	X[23]	X[23]
Prior and concomitant medications[24]	X	X	X	X	X	X	X	X	X	X	X	X

1. Or after early withdrawal.
2. Prior to wound punching.
3. Re-check of eligibility criteria

4. Weight only.
5. Drug tests may also be performed at 1 to 2 additional random occasions during the study.
6. Females only. Screening: blood/serum tests, other visits: urine test.
7. Pre-dose and 1 and 4 hours (before the subject leaves the clinic) after dose.
8. Prior to the first IMP administration. Can be performed at screening or at any time between screening and the first IMP administration.
9. One biopsy (wound punch) should be saved for baseline histopathology analysis as described in Section 11.8.2.
10. One sample of resuspended drug product for viable count, see Section 11.6. Cohort 1 only.
11. After wound punching, prior to IMP application and 4 h post application.
12. Local tolerability assessed by the Investigator (direct observation) and by 3 to 5 independent evaluators using photographs. Day 1: after wound punching, prior to IMP application and 4 hours post application. Subjects assess local tolerability from Day 2.
13. Local tolerability during the long-term follow-up assessed by the Investigator only.
14. CXCL12 blood sampling prior to wound punching at 9:00±2 hours, 1 hour post the last wound punch (within 5 minutes of start of IMP application, which should start 1 hour (±10% post the last wound punch) and 1 hour (±10%) after the last IMP application.
15. CXCL12 blood sampling at 9:00±2 hours. On Day 2: 24h±2 hours post dose but no later than 11:00. On Day 3 and 14, also plating of blood samples prior to centrifugation for blood culturing, see Section 11.8.1 and Section 11.8.1.2.
16. 48 hours after IMP administration (one biopsy from a CXCL12 treated wound and one from a placebo treated wound)
17. Wound healing assessed by the Investigator (direct observation) and by 3 to 5 independent evaluators using photographs.
18. Wound area assessed by 3 to 5 independent evaluators using photographs and the Image J software (National Institutes of health [NIH]) or equivalent.
19. Baseline wound area measurements based on photos taken after wound punching prior to IMP application.
20. Scar tissue formation assessed by the Investigator (direct observation) and by 3 to 5 independent experts using photographs at Visit 6. Scar tissue formation during the long-term follow-up assessed by the Investigator only. Normal/abnormal and Vancouver scar scale. One assessment per wound.
21. Dressing if indicated.
22. From administration of IMP.
23. Collection of AEs including, but not limited to, mortality, development of any new/recurrent cancer, development of infection, immunogenicity related reactions.
24. For definitions of prior and concomitant medications, see Section 11.3.6.

8.1.2 *Multiple Ascending Dose (MAD)*

Part II of the study will explore multiple ascending dosing of *L. reuteri* expressing CXCL12 (activated ILP100-DP) in 3 sequential cohorts, each of 8 subjects. For each subject in a cohort, activated ILP100-DP, placebo and saline (NaCl) will be randomized to in total 8 wounds, 4 on the right arm and 4 on the left, in a 4:2:2 ratio (i.e. 4 wounds will be treated with activated ILP100-DP, 2 wounds will be treated with placebo and 2 wounds will be treated with saline as randomized). Activated ILP100-DP will be randomized to one of the arms and placebo and saline to the other arm. The IMPs will be administered on Day 1, Day 2 and Day 3 and then 3 times a week over the course of 3 weeks (in total 10 doses).

The proposed dose levels are $5x10^5$, $5x10^7$ and $1x10^9$ CFU/cm^2 wound area. The rationale for the starting dose and the planned dose escalation is detailed in Section 8.3. The doses, dose escalations and the dosing schedule may be adjusted based on emerging knowledge of safety and tolerability data observed in the SAD part of the study.

Subjects will come for 13 visits to the research clinic for screening, treatment and initial follow-up (Visit 1 to Visit 13), see Table 8.1-2 for details. After Visit 13, a 5-year long-term follow-up period (Visit 14 to 21) will be initiated as detailed below.

Treatment phase (Visit 1 to Visit 13)

Screening (Visit 1) will take place from Day -28 to Day -1. At Visit 2, eligible subjects will be admitted to the research clinic on Day 1 for pre-dose safety assessments and full-thickness wound punching with a biopsy punch (6 mm in diameter) on the ventral aspect of the upper arms (4 wounds/arm). The wounds should be separated by approximately 4 cm. Each wound will be numbered using a permanent marker (repeated at all post-dose visits). Prior to wound punching, subjects will be treated with local anesthesia (injected and/or topical) and the area will be cleaned with antiseptics (70% ethanol). The wound punching procedure will be detailed in a separate manual.

Subsequent to wound punching, the first dose of the IMP (activated ILP100-DP, placebo and saline) will be topically applied to each of the wounds in accordance with the randomization list. There should be 1 hour (±10%) between the last wound punch and the start of IMP application to allow for pre-dose study assessments and hemostasis. Two unblinded persons will take part of the administration process, one will perform the application of IMP and the other one will confirm that the correct treatment is administered to the intended wound, i.e. in accordance with the randomization list. The IMP administration will be documented by video recording. Each wound will be dressed separately using an adhesive, transparent film, isolating the wounds from each other. An elastic, tubular bandage will be used on top of the films. Following IMP administration on Day 1 and Day 2, the wounds will be covered with adhesive, transparent film around the clock for 2 days and 2 nights (except during wound evaluations and IMP application). From Day 3 and onwards, the wounds will be treated with IMP and covered with adhesive, transparent film for 1 hour after IMP application where after the film will be removed. The wounds will be allowed to air dry and will then be separately covered with a non-occlusive dressing that can be used during showering and protects against contamination of viruses and bacteria. For details on the wound incision and IMP application processes, refer to Section 11.4 and Section 10.6, respectively.

The subjects will be carefully monitored by clinical staff during and after first dose and will remain at the research clinic for approximately 4 hours after dose. Vital signs and ECG will

be checked as detailed in Table 8.1-2. There is immediate access to equipment, qualified staff and an ICU in case of an acute emergency. Up to 4 subjects will be dosed on the same day.

The wounds will be photographed in a standardized setting according to instructions specified in a separate manual, and briefly summarized in Section 11.5, before treatment on Day 1, pre-treatment at the subsequent dosing visits, and at all follow-up visits to the research clinic. Selected members of the clinical staff will receive photography training prior to handling of the camera equipment. In addition, wounds will be analyzed using a 3D camera as outlined in Section 11.9.1.

Following the first dose on Day 1, subjects will come for 10 additional visits between Day 2 and Day 21 (Visits 3 to 12). The last dose will be given on Day 19 (Visit 11). Safety will be assessed by vital signs, safety laboratory parameters, local tolerability, AEs, use of concomitant medications and CXCL12 ADA analysis at visits outlined in Table 8.1-2.

The wounds must not at any visit be cleaned (unless necessary for safety reasons, e.g. due to an infection). The area surrounding the wounds should then be carefully cleaned with water if necessary. At visits when swabbing is performed, the area around the wound should be cleaned after swabbing if necessary. Before the subject leaves the research clinic, the wound should be dressed until healed.

Blood sampling for analysis of CXCL12 and *L. reuteri* systemic levels and a swab of the area surrounding the wounds and feces sampling for analysis of any presence of *L. reuteri* R2LC colonies containing the pSIP411 plasmid will be performed at time points and visits specified in Table 8.1-2.

For exploratory purposes, microcirculation assessments will be performed and dressings will be collected at time points outlined in Table 8.1-2.

An end-of-treatment phase visit (Visit 13) will take place on Day 32 (± 1 day) or after early withdrawal.

Long-term follow-up (Visit 14 to Visit 21)

After Visit 13, a 5-year long-term follow-up period (Visit 14 to 21) will be initiated. During the first year, subjects will visit the research clinic 6 weeks and 3, 6 and 12 months after treatment (Visit 14 to Visit 17). Following Visit 17, subjects will be followed-up yearly by visits to the research clinic.

Physical examination, AE and concomitant medication questioning, blood sampling for CXCL12 ADA analysis, photography (standard camera and 3D camera), local tolerability assessment (investigator), assessment of scar tissue formation, skin swab and feces sampling for analysis presence of *L. reuteri* will be performed at visits outlined in Table 8.1-2.

Subjects will be urged to contact the clinic in between visits, including long-term follow-up visits, in case of e.g. development of any new/recurrent cancer, development of infection, immunogenicity related reactions or local reactions associated with the wound/scar area. If considered relevant by the Investigator, extra visits will be scheduled.

General

The investigator will assess local tolerability, wound healing and scar formation by direct observation. The subjects will assess local tolerability (pruritus and pain). Three to five independent experts will assess local tolerability, wound area, wound healing and scar

formation by using the wound/scar photographs. For details on timing of assessments, refer to Table 8.1-2.

Before initiating a new cohort, all subjects in the previous cohort must have been treated and 21 days safety and tolerability data for all treated subjects must have been evaluated by the iSRC, see Section 8.4.3. Once Day 21 safety and tolerability data for the last subject in each cohort has been collected, there will be at least 1 week between dose escalations.

Each subject is expected to participate for approximately 49 days (including a 28-day screening period) in the first phase of the MAD part and for an additional 5 years in the long-term follow-up.

The schedule of events is shown in Table 8.1-2. Study assessments are described in Section 11.

Table 8.1-2 Schedule of events, MAD part

	Treatment phase							Long-term follow-up					
	Year 1											Year 2-4	Year 5
	Screening	Randomization & treatment	Treatment visits Dosing 3 times/week, in total 10 doses			Outpatient	End-of-treatment phase[1]	Follow-up	Follow-up	Follow-up	Follow-up	Follow-up	End-of-study
**Visit number**	1	2	3	4	5-11	12	13	14	15	16	17	18-20	21
			Time points in relation to first dose (Day 1)					Time points in relation to last dose (Day 19)					
**Assessment / Day**	Day -28 to -1	Day 1	Day 2	Day 3	Day 5, 8, 10, 12, 15, 17, 19 ±1 day on Days 12 to 19	Day 21 ± 1 day	Day 32 ± 1 day	6 weeks ± 3 days	3 months ± 1 week	6 months ± 1 week	12 months ± 1 week	Once yearly ± 2 weeks	5 years ± 1 month
Informed Consent	X												
Inclusion/exclusion criteria	X	X[2,3]											
Demographics	X												
Medical/surgical history	X												
Physical examination	X	X[2]					X	X	X	X	X	X	X
Weight and height	X										X[4]	X[4]	X[4]
Urine analysis (dip stick)	X	X[2]					X						
HIV, Hepatitis B and C	X												
Urine drug screen[5]	X	X[2]				X	X	X	X	X	X	X	X
Alcohol breath test	X	X[2]				X	X	X	X	X	X	X	X
Pregnancy test	X[6]	X[6]				X[6]	X						
12-lead safety ECG	X	X[2,7]			X[8]		X						
Vital signs (BP & pulse)	X	X[2,7]		X	X	X	X						
Body temperature		X[2]											
Hematology, coagulation and clinical chemistry	X	X[2]	X				X						

	**Treatment phase**							**Long-term follow-up**					
	**Year 1**											**Year 2-4**	**Year 5**
	**Screening**	**Randomization & treatment**	**Treatment visits Dosing 3 times/week, in total 10 doses**			**Outpatient**	**End-of-treatment phase[1]**	**Follow-up**	**Follow-up**	**Follow-up**	**Follow-up**	**Follow-up**	**End-of-study**
**Visit number**	**1**	**2**	**3**	**4**	**5-11**	**12**	**13**	**14**	**15**	**16**	**17**	**18-20**	**21**
			**Time points in relation to first dose (Day 1)**					**Time points in relation to last dose (Day 19)**					
**Assessment / Day**	**Day -28 to -1**	**Day 1**	**Day 2**	**Day 3**	**Day 5, 8, 10, 12, 15, 17, 19 ±1 day on Days 12 to 19**	**Day 21 ± 1 day**	**Day 32 ± 1 day**	**6 weeks ± 3 days**	**3 months ± 1 week**	**6 months ± 1 week**	**12 months ± 1 week**	**Once yearly ± 2 weeks**	**5 years ± 1 month**
Randomization		X[2]											
CXCL12 ADA sample		X[9]				X		X			X	X	X
Wound incision by punch biopsy		X											
IMP administration		X	X	X	X								
IMP for viable count		X[10]			X[10]								
Wound/scar photography		X[11]	X	X	X	X	X	X	X	X	X		
3D camera		X[11]	X	X	X	X	X	X	X	X	X		
Local tolerability[12]		X[11, 12]	X[12]	X[12]	X[12]	X[12]	X[12]	X[13]	X[13]	X[13]	X[13]	X[13]	X[13]
Blood sampling for analysis of CXCL12 and *L. reuteri*		X[14]	X[15]	X[15, 17]	X[16, 17]	X[18]							
PBMC sampling		X[19]			X[19]								
Wound healing[20]					X	X	X						
Wound area[21]		X[22]	X	X	X	X							
Scar tissue formation[23]					X	X	X	X	X	X	X	X	X
Skin swab for analysis of *L. reuteri*				X	X[24]	X	X	X			X	X	X
Hand out of feces sampling kit	X				X[25]		X			X	X	X	X

	**Treatment phase**							**Long-term follow-up**					
	**Year 1**											**Year 2-4**	**Year 5**
	**Screening**	**Randomization & treatment**	**Treatment visits Dosing 3 times/week, in total 10 doses**			**Outpatient**	**End-of-treatment phase**[1]	**Follow-up**	**Follow-up**	**Follow-up**	**Follow-up**	**Follow-up**	**End-of-study**
**Visit number**	**1**	**2**	**3**	**4**	**5-11**	**12**	**13**	**14**	**15**	**16**	**17**	**18-20**	**21**
			**Time points in relation to first dose (Day 1)**					**Time points in relation to last dose (Day 19)**					
**Assessment / Day**	**Day -28 to -1**	**Day 1**	**Day 2**	**Day 3**	**Day 5, 8, 10, 12, 15, 17, 19 ±1 day on Days 12 to 19**	**Day 21 ± 1 day**	**Day 32 ± 1 day**	**6 weeks ± 3 days**	**3 months ± 1 week**	**6 months ± 1 week**	**12 months ± 1 week**	**Once yearly ± 2 weeks**	**5 years ± 1 month**
Feces sample for analysis of *L. reuteri*		X[9]				X[26]		X			X	X	X
Microcirculation			X[27]		X[27]								
Dressing		X	X	X	X	X[28]							
Microbe analysis (dressing collection)			X[29]	X[29]									
Baseline symptoms	X												
AEs		X[30]	X	X	X	X	X	X[31]	X[31]	X[31]	X[31]	X[31]	X[31]
Prior and concomitant medication[32]	X	X	X	X	X	X	X	X	X	X	X	X	X

1. Or after early withdrawal.
2. Prior to wound punching.
3. Re-check of eligibility criteria.
4. Weight only.
5. Drug tests may also be performed at 1 to 2 additional random occasions during the study.
6. Females only. Screening: blood/serum tests, other visits: urine test.
7. Pre-treatment and 1 and 4 hours (i.e. before the subject leaves the clinic) after dose
8. ECG on Day 10.
9. Prior to first IMP administration. Can be performed at screening or at any time between screening and the first IMP administration.
10. Day 1, cohort 1 only, and Day 19, all cohorts, one sample of resuspended drug product for viable count, see Section 11.6. All cohorts.
11. After wound punching, prior to IMP application and 4 h post application.
12. Local tolerability assessed by the Investigator (direct observation) and by 3 to 5 independent evaluators using photographs. Day 1: after wound punching, prior to IMP application and 4 hours post application. Subjects assess local tolerability from Day 2.

13. Local tolerability during long-term follow-up assessed by the investigator only.
14. CXCL12 blood sampling prior to wound punching at 9:00±2 hours, 1 hour post the last wound punch (within 5 minutes of start of IMP application, which should start 1 hour (+/10% post the last wound punch) and 1 hour (±10%) after the last IMP application.
15. Pre-treatment. The pre-treatment sample should be taken at 9:00±2 hours.
16. Pre-treatment on Day 5, 8, 15 and 19. The pre-treatment samples should be taken at 9:00±2 hours.
17. On Day 3, 5, 8, 15 and 21, also plating of blood samples prior to centrifugation for culturing, see Section 11.8.1 and Section 11.8.1.2.
18. The CXCL12 blood sample should be taken at 9:00±2 hours.
19. Pre-dose Day 1 and pre-dose on Day 19, see Section 11.8.1.
20. Wound healing assessed by the Investigator (direct observation) and by 3 to 5 independent experts using photographs. No assessment by independent experts on Day 17.
21. Wound area assessed by 3 to 5 independent experts using photographs and the Image J software (NIH) or equivalent.
22. Baseline wound area measurements based on photos taken after wound punching prior to IMP application.
23. Scar tissue formation assessed by the Investigator (direct observation) and by 3to 5 independent experts using photographs Visit 9 to Visit 12. Scar tissue formation during the long-term follow-up assessed by the Investigator only. Normal/abnormal and Vancouver scar scale. One assessment per wound.
24. Day 5, Visit 5.
25. Day 15, Visit 9.
26. At any time between Day 15 and Day 21.
27. Just prior to treatment on Day 2, Day 8 and Day 15.
28. Dressing if indicated.
29. Dressing collection on Day 2 (applied Day 1), Day 3 (applied Day 2).
30. From administration of IMP.
31. Collection of AEs including, but not limited to, mortality, development of any new/recurrent cancer, development of infection, immunogenicity related reaction.
32. For definitions of prior and concomitant medications, see Section 11.3.6.

8.2 Rationale for study design

The European Medicines Agency (EMA) guideline on strategies to identify and mitigate risks for FIH and early clinical trials with IMPs (EMEA/CHMP/SWP/28367/07 Rev. 1) has been considered as have the European Directive 2001/18/EC on the deliberate release into the environment of GMOs and the guidance on the environmental risk assessment of genetically modified organisms from the EMA CHMP/GTWP/125491/06. A 5-year long-term follow-up has been included in line with the recommendations given in EMEA/CHMP/GTWP/60436/2007.

The SAD/MAD design of the study is based on the aim to study safety, tolerability and exposure of selected doses of activated ILP100-DP in a limited number of healthy volunteers.

The design is adaptive to allow for flexible dose escalation and involves careful monitoring of the subject’s well-being. The study will provide important safety and tolerability data and preliminary efficacy data to support the design of further studies, both in healthy volunteers and in patients.

A placebo control will be used to establish the frequency and magnitude of changes in endpoints that may occur in the absence of active treatment. Subjects will serve as his/her own control receiving both placebo and active treatment on the same occasion. The placebo component consist of the ILP100-DP dilution buffer and the activation peptide SppIP. The dilution buffer contains sucrose, which may have a wound healing effect. Recombinant CXCL12 is not used as placebo since the half-life is too short (approximately 6 seconds) to provide clinically relevant data (Laguri *et al.*, 2007; Lambeir *et al.*, 2001). *Lactobacillus* wild-type is not used as placebo since extensive testing in non-clinical studies have shown that it has no effect on wound healing.

Saline (0.9 % NaCl) will be used as a “no treatment” control for several purposes, 1) all wounds must be treated with a solution to keep the blind, 2) all wounds must be exposed to comparable moisture during the study since moisture is a parameter that affects wound healing and 3) NaCl will identify any wound healing properties of sucrose in the dilution buffer used for both ILP100-DP and placebo.

Randomization will be used to minimize bias in the assignment of treatments to wounds.

Blinded treatment will be used to reduce potential bias during data collection and evaluation of endpoints.

All subjects in the MAD part will be treated at 10 dosing occasions over 3 weeks despite wound healing status. For this FIH study with focus on safety, and in order to obtain the same exposure time for all subjects, this approach was chosen rather than to continue treatment only until wound healing is achieved.

8.3 Selection of starting dose and rationale for planned dose escalation

8.3.1 *Selection of starting dose and rationale for dose escalation*

The dose is determined per area of the wound and is given in the unit colony forming units (CFUs) per cm^2.

The planned starting dose of activated ILP100-DP in the SAD part ($5x10^4$ CFU/cm^2 wound area) has been selected to give an adequate safety margin based on the preclinical studies. The planned doses to be administered in cohorts 2 and 3 of the SAD are $5x10^7$ and $1x10^9$ CFU/cm^2 wound area.

The highest dose used in non-clinical studies in mice with uncovered wounds was to $2x10^{10}$ CFU/cm^2. The highest dose used in the non-clinical work in minipigs with covered wounds was $1.1x10^{10}$ CFU/ cm^2. No treatment-related systemic or local effects have been detected at any dose level tested. The SAD starting dose of $5x10^4$ CFU/cm^2 wound area is 400 000 times less the maximum dose given to mice and 220 000 times less the maximum dose given to minipigs (Table 8.3-2).

The lowest dose of $5x10^4$ CFU/cm^2 wound area in the SAD part has been selected based on the hypothesis that this dose will be outside the lower spectrum of the therapeutic window. This is based on experiments in mice where $3.3x10^2$ CFU/cm^2 failed to accelerate wound healing. The working hypothesis is that $5x10^7$ CFU/cm^2 is a therapeutic dose and the highest dose of $1x10^9$ CFU/cm^2 is 11 times lower than the highest dose given to minipigs ($1.1x10^{10}$ CFU/cm^2) at which no toxicity effects were detected. On a total dose basis (CFU/m^2 body surface are [BSA]), the highest dose to be administered in the SAD is 517 times lower than that administered in minipigs (Table 8.3-2). The total dose of the SppIP (ng/m^2 BSA) to be administered in the SAD is 42 times lower than that administered to minipigs. Hence, the human safety margin is considered sufficient.

The preliminary starting dose in the MAD part ($5x10^5$ CFU/cm^2 wound area) has been chosen based on the assumption that the $5x10^4$ CFU/cm^2 wound area dose, given as starting dose in the SAD part, will be non-efficacious. The starting dose in the MAD part will be finally set once the iSRC has evaluated the safety and tolerability data from the SAD part. The planned doses to be administered in cohorts 2 and 3 of the MAD are $5x10^7$ and $1x10^9$ CFU/cm^2 wound area. Doses in the MAD will be applied to the wounds approximately 3 times a week during 3 weeks (in total 10 doses).

Depending on the results in the SAD part the dose range might be narrowed in the MAD part to better capture differences and understand what dose is the most likely to have profile suitable for a therapeutic dose. If the $5x10^4$ CFU/cm^2 dose in the SAD part indicates an efficacy similar to the other doses, the lowest dose in the MAD part will be changed to $5x10^3$ CFU/cm^2. In case of no, or a small effect of the $5x10^4$ CFU/cm^2 dose in the SAD (which is the expected scenario), the starting dose in the MAD part will be $5x10^5$ CFU/cm^2.

For a summary of the planned doses in the present study, see Table 8.3-1. The administered volume of IMP is always 50 µL.

For further details on the prerequisites for the transition from single to multiple dosing, refer to Section 8.4.2.

Table 8.3-1 Overview of intended dosing of ILP100-DP (CFU/mL CFU or ng SppIP/wound, cm^2 of wound surface and per BSA)

Cohort	Wound area (cm^2)	Treated wounds/ Total no of wounds	Material	Volume applied per wound µl	CFU/wound (*L. reuteri*)	CFU/ cm^2 (*L. reuteri*)	Exposure per body surface CFU (*L. reuteri*)/ m^2 BSA
					**ng/wound (SppIP)**	**ng/cm^2 (SppIP)**	**ng SppIP/ m^2 BSA**
1 SAD	0.28 Ø 6 mm	2/4	ILP100-DP activated	50	$1.4x10^4$	$5x10^4$	$1.7x10^4$
					5	18	6.2
2 SAD	0.28 Ø 6 mm	2/4	ILP100-DP activated	50	$1.4x10^7$	$5x10^7$	$1.7x10^7$
					5	18	6.2
3 SAD	0.28 Ø 6 mm	2/4	ILP100-DP activated	50	$2.8x10^8$	$1x10^9$	$3.5x10^8$
					5	18	6.2
1 MAD (10 doses)	0.28 Ø 6 mm	4/8	ILP100-DP activated	50	$1.4x10^5$	$5x10^5$	$3.5x10^5$
					5	18	12.3
2 MAD (10 doses)	0.28 Ø 6 mm	4/8	ILP100-DP activated	50	$1.4x10^7$	$5x10^7$	$3.5x10^7$
					5	18	12.3
3 MAD (10 doses)	0.28 Ø 6 mm	4/8	ILP100-DP activated	50	$2.8x10^8$	$1x10^9$	$6.9x10^8$
					5	18	12.3

*Body surface area (BSA) = 1.6 m^2

8.3.2 *Maximum exposure and dose*

In the 4-week repeated dose toxicity study in minipigs, 3 levels of exposure of the *L. reuteri* R2LC expressing CXCL12 were used, see Section 6.1.6. There was no treatment-related toxicity detected at any of the dose levels used (Section 6.1.6). The highest dose given to minipigs was $1.1x10^{10}$ CFU/cm^2.

The dose margins between the highest dose given to mini pigs ($1.1x10^{10}$ CFU/cm^2) and the proposed starting doses and maximum doses in the SAD and MAD parts, respectively are outlined in Table 8.3-2. The maximum dose of activated ILP100-DP to be administered in the present study is $1x10^9$ CFU/cm^2 wound area.

Table 8.3-2 Dose margins between high dose animals of the minipig study and the proposed starting and final doses of the SAD and MAD parts

Dose Parameter	SAD		MAD	
	Starting Dose	Final Dose	Starting Dose	Final Dose
CFU/cm^2	220 x 10^3	11	22 x 10^3	11
CFU/m^2 BSA	10.6 x 10^6	517	517 x 10^3	260

8.4 Dose escalation strategy

An adaptive dosing strategy will be applied to allow for flexible and safe dose escalation. Refer to Section 8.3.1 for the planned dose escalation.

Intermediate dose levels and/or additional dose groups may be considered if recommended by the iSRC (Section 8.4.3). The actual doses given will be guided by the iSRC recommendations and available safety and tolerability data will determine whether or not to follow the above provisional dose levels. Every dose step is thus adjustable.

Depending on results obtained during the study, the number of dose levels/regimens and planned sampling times might have to be adjusted. These adjustments will be documented in non-substantial amendments to the clinical study protocol (CSP).

8.4.1 *Stopping criteria for dose escalation*

The Principal Investigator and the iSRC (Section 8.4.3) will follow the recommendations and grading system of Common Terminology Criteria for Adverse Events (CTCAE) v5.0, see Section 11.7.1.7 but also take into account the recommendations published by Sibille *et al.* 2010, which is an adaptation to FIH studies of the grading systems previously proposed by National Cancer Institute (NCI), World Health Organization (WHO), National Institute of Health (NIH) and the US Food and Drug Administration (FDA). The grade, the frequency of AEs and the blindness will be considered.

A rolling review of emerging safety data will be performed throughout the study taking into account any AEs and their relation to pharmacodynamic (PD) effects, the number of subjects in whom they occur, concurrency of more than one event within the same subject and any trends. Changes from baseline measurements will also be considered and not just absolute cut-off based on upper- or lower limits of normal that might apply for healthy volunteers.

Trends or safety signals described above, which are not necessarily covered by the stopping criteria in Table 8.4-1 may warrant the scheduling of ad hoc iSRC meetings after which the iSRC will make recommendations to Sponsor on e.g. whether to terminate the study or to stop dosing in individual subjects or a certain cohort.

Stopping criteria related to exposure (area under the curve [AUC] and maximum plasma concentration [C_{max}] of CXCL12) are considered as not applicable for the study since the systemic exposure of CXCL12 is predicted to be very low or undetectable.

Table 8.4-1 Dose escalation and stopping rules

Level	Action taken
If no subject has an SAE considered at least possibly related to the IMP administration, i.e. a serious adverse drug reaction (SADR)	Escalate to the next higher dose level.
If 1 subject has an SAE assessed as at least possibly related to IMP administration	1) Stop of further dosing at this dose level<br>2) If the SAE is clearly associated with a single, or multiple wound(s): unblinding of these wounds. Only voting members of the iSRC will be unblinded.<br>If the etiology of the SAE is not obviously associated with a single, or multiple, wound(s), no unblinding will be performed. The SAE will be regarded as related to ILP100-DP.<br>3) Evaluation by iSRC. The iSRC makes recommendation to Sponsor.<br>Subject meets stop criterion: stop further dosing at this dose level for all subjects. Sponsor decides if the study will be terminated or if dosing will commence at a lower dose level or at an intermediate dose level.
If 2 subjects on active treatment have severe, non-serious AEs assessed at least possibly related to the IMP administration (independent of within or not within the same System organ class [SOC])	1. Stop dosing of subjects with severe, non-serious potential ADRs.<br>2. If the AEs are clearly associated with a single, or multiple wound(s): unblinding of these wounds. Only voting members of the iSRC will be unblinded.<br>If the etiology of the AEs is not obviously associated with a single, or multiple, wound(s), no unblinding will be performed. The AEs will be regarded as related to ILP100-DP.<br>3. Evaluation by iSRC. The iSRC makes recommendation to Sponsor.<br>4. Sponsor decides how to proceed:<br>Both subjects meet stop criterion: stop further dosing at this dose level for all subjects. Sponsor decides if the study will be terminated or if dosing should commence at a lower dose level or at an intermediate dose level.
If 2 subjects in different cohorts have SAEs assessed as at least possibly related to IMP administration	1. Stop dosing of subjects with potential SADR.<br>2. If the SAEs are clearly associated with a single, or multiple wound(s): unblinding of these wounds. Only voting members of the iSRC will be unblinded.<br>If the etiology of the SAEs is not obviously associated with a single, or multiple, wound(s), no unblinding will be performed. The SAEs will be regarded as related to ILP100-DP.<br>3. Evaluation by iSRC. The iSRC makes recommendation to Sponsor.<br>Both subjects meet stop criterion: Termination of study.

8.4.2 *From single to multiple dosing*

The SAD part of the study will be completed prior to the start of the MAD part. Following completion of the SAD part, the iSRC will evaluate all safety and tolerability data from the SAD and, if necessary, suggest adjustments to dose levels or dosing regimens in a written recommendation to the Sponsor. Dose adjustments or adjustments to dose regimens may be recommended based on the SAD data. If no safety or tolerability concerns are identified, the MAD part will commence as decided by the Sponsor. The iSRC recommendation as well as Sponsor’s decision, will be documented in a non-substantial amendment.

8.4.3 *Internal safety review committee*

Before initiating a new cohort, all subjects in the previous cohort must have been treated and 3 days (SAD) and 21 days (MAD) safety and tolerability data for all treated subjects must have been evaluated by the iSRC. Based on emerging safety and tolerability data, the amount of required safety data to be reviewed after a completed cohort might be adjusted. Details regarding timing of iSRC review and the data to be reviewed and the documentation procedure will be provided in a separate iSRC charter.

The voting members of the iSRC will consist of the Principal Investigator, the Medical Monitor (i.e. the Sponsor’s medically responsible person) or delegate and an independent, external clinical expert. The clinical research manager (CRM), and additional Sponsor representatives will be invited as appropriate. Further internal or external experts may be consulted by the iSRC as necessary.

An adaptive dosing strategy will be applied to allow for a flexible and safe dose escalation. The planned dose escalation is outlined in Section 8.3.1. The actual doses given in each cohort will be guided by the iSRC recommendations based on available safety and tolerability data. Every dose step is thus adjustable and the recommendation to the Sponsor may be to continue with a higher or lower dose than the intended dose, repeat the same dose level, continue with an intermediate dose level or to stop dosing.

The decision of the iSRC on the recommendation of the next dose level will be taken in consensus between the iSRC members and documented as appropriate. In case there is disagreement between the 3 voting members, the most conservative approach will be taken. It is not acceptable to repeat a dose level where any of the dose escalation stopping rules have been met.

The treatment code for individual wounds may be broken by the iSRC during the assessment process (partial un-blinding) in accordance with stopping criteria described in Table 8.4-1. The medical staff and the subjects will still be blinded for the treatments (active drug, placebo or saline) to be administered in the subsequent dose groups/cohorts in order to minimize bias. If unblinding was considered necessary, the iSRC meeting may consist of a closed part and an open part.

9 STUDY POPULATION

Prospective approval of protocol deviations to eligibility criteria, also known as protocol waivers or exemptions, is not permitted.

9.1 Recruitment

The subjects will be recruited from [redacted] database of healthy volunteers and from advertising in media (including social media).

9.2 Screening and enrolment log

Investigators must keep a record of all screened subjects even if they were not subsequently included in the study. This information is necessary to verify that subjects were selected without bias. The reason for screen failure should be stated for all subjects screened but not included. The reason for withdrawal should be stated for all subjects included but not completed.

A screening number will be allocated to each subject in connection to the informed consent process at the Screening visit. The screening number is automatically generated in the electronic case report Form (eCRF). The screening number will allow identification of subjects irrespective of their possible eligibility for the study.

Subjects included and randomized will be assigned a randomization number (1101, 1102 etc. the SAD, cohort 1; 2101, 2102 etc. in the MAD, cohort 1 and so on). The first digit will correspond to the study part, the second digit will correspond to the cohort and the 3rd and 4th digits correspond to the subject number.

If a subject cannot receive the planned dose of IMP within 27 days after screening (*i.e.*, the time interval between signing informed consent until dose administration) the subject should be re-screened before proceeding in the study. For details regarding re-screening, refer to Section 9.7.

9.3 Number of subjects

Approximately 90 subjects will be screened (approximately 30 for the SAD and 60 for the MAD) to achieve a total of 36 randomized and dosed subjects. The number may increase if additional dose cohorts need to be investigated and/or if recommended by the iSRC.

SAD: 12 healthy subjects will be randomized and dosed.

MAD: 24 healthy subjects will be randomized and dosed.

To account for potential drop-outs and/or additional cohorts depending on recommendations from the iSRC, additional subjects might be included in the study.

9.4 Inclusion criteria

For inclusion in the study, subjects must fulfil the following criteria:

1. Willing and able to provide a written informed consent for participation in the study.
2. Healthy males and females aged ≥25 and ≤45 years.
3. Willing and able to comply with study requirements.
4. Non-obese subjects with a body Mass Index (BMI) ≥18.0 and ≤30.0 kg/m^2 and weight at least 50 kg and no more than 120 kg at screening.
5. Clinically normal medical history, physical findings, vital signs, ECG and laboratory values at the time of screening, as judged by the Investigator.
6. Women of child bearing potential (WOCBP) must agree to use a highly effective method of contraception with a failure rate of < 1% to prevent pregnancy (combined [estrogen and progestogen containing] hormonal contraception associated with inhibition of ovulation [oral, intravaginal, transdermal], progestogen-only hormonal contraception associated with inhibition of ovulation [oral, injectable, implantable], intrauterine device [IUD]or intrauterine hormone-releasing system [IUS]) from at least 4 weeks prior to dose until 3 months after the last dose of IMP and until confirmation of negative 6 weeks skin swab and feces analyses concerning *L. reuteri* R2LC and/or pILP100 findings. Their male partner must agree to use a condom during the same time frame.

   Women of non-childbearing potential are defined as pre-menopausal females who are sterilized (tubal ligation or permanent bilateral occlusion of fallopian tubes); or post-menopausal defined as 12 months of amenorrhea (in questionable cases a blood sample with simultaneous detection of follicle stimulating hormone [FSH] 25-140 IE/L and estradiol <183 pmol/l is confirmatory).

   Male subjects must be willing to use condom or be vasectomized to prevent pregnancy and drug exposure of a partner and refrain from donating sperm from the date of dosing until 3 months after the last dose of IMP and until confirmation of negative 6 weeks skin swab and feces analyses concerning *L. reuteri* R2LC and/or pILP100 findings. Their female partner of child-bearing potential must use contraceptive methods with a failure rate of < 1% to prevent pregnancy (see above).
7. Subjects must agree to refrain from donating blood/blood components and organs from the first dose of IMP until 3 months after the last dose of IMP and until confirmation of negative 6 weeks skin swab and feces analyses concerning *L. reuteri* R2LC and/or pILP100 findings.

9.5 Exclusion criteria

Subjects must not enter the study if any of the following exclusion criteria are fulfilled:

1. Pregnant or lactating female.
2. History of any clinically significant disease or disorder including, but not limited to, gastrointestinal, cardiovascular, cerebrovascular, pulmonary, neurologic, renal, liver disease which, in the opinion of the Investigator, may either put the subject at risk because of participation in the study, or influence the results or the subject´s ability to participate in the study.

3. Clinically significant hypertension or circulatory disease.
4. History or presence of any bleeding disorder including prolonged or habitual bleeding and subjects who are taking blood-thinning medications such as warfarin within 1 month before the IMP application.
5. Any clinically significant illness, medical/surgical procedure or trauma within 4 weeks of the planned first administration of IMP.
6. Any planned major surgery within the duration of the study.
7. Birthmarks, tattoos or any skin abnormality at the site of wounding, as assessed by the investigator.
8. Skin condition or skin quality that, in the opinion of the Investigator, may increase the risk for abnormal scar formation or other injury to the patient or that may obstruct the conduct of the study assessments.
9. Suspicion of melanoma at the site of the biopsy.
10. Use of topical dermatological drug therapy on arms.
11. History or presence of skin diseases.
12. History of keloid formation of skin scars.
13. Seropositive for human immunodeficiency virus (HIV), Hepatitis B surface antigen (HBsAg), or Hepatitis C virus (HCV) antibodies.
14. Positive screen for drugs of abuse or a positive alcohol result at screening or admission to the research clinic.
15. History of severe allergy/hypersensitivity or ongoing allergy/hypersensitivity, as judged by the Investigator, history or ongoing allergy/hypersensitivity to the adhesive material of the dressings or history of hypersensitivity to drugs with a similar biological substance, a similar chemical structure or class to the IMP or any of the excipients or local anesthetics
16. Regular use of any prescribed or non-prescribed medication including antibiotics (in particular erythromycin, clindamycin or linezolid), antacids, analgesics, herbal remedies, vitamins and minerals within 2 weeks prior to the first application of IMP, except occasional intake of paracetamol (maximum 2 000 mg/day; and not exceeding 3 000 mg/week), at the discretion of the Investigator and nasal decongestants without cortisone or antihistamine for a maximum of 10 days, at the discretion of the Investigator.
17. Use of any vasoactive (constrictor or dilator) medication or any prescription or OTC drugs that could modulate blood flow within 2 weeks of the screening visit. Examples of such drugs include nitroglycerine, antihypertensive drugs, antihistamines, NSAIDs, aspirin and OTC cough/common cold products containing antihistamines and/or either phenylpropanolamine or phentolamine during the study up until Visit 6 in the SAD part and Visit 13 in the MAD part.
18. Use of systemic corticosteroids within 2 months prior to the screening visit.
19. Planned treatment or treatment with another investigational drug within 3 months prior to Day -1 or has participated in any other clinical study that included drug treatment with less than 3 months between administration of last dose and first dose of IMP in this study. Subjects consented and screened but not dosed in previous Phase I studies are not excluded.
20. Current, or history of, alcohol abuse/alcoholism/excessive alcohol use and/or use of anabolic steroids or drugs of abuse as judged by the Investigator.

21. Any use of alcohol within 48 hours of admission to the research clinic.
22. Plasma donation within 1 month of screening or any blood donation or corresponding blood loss during 3 months prior to screening.
23. Investigator considers the subject unlikely to comply with study procedures, restrictions and requirements.
24. Abnormal vital signs at screening after 10 min supine rest defined by either:
    - Diastolic blood pressure <50 or >90 mmHg, or
    - Systolic blood pressure <90 or >150 mmHg, or
    - Pulse <40 or >90 bpm
25. Prolonged QTcF (>450 ms), cardiac arrhythmias or any clinically significant abnormalities in the resting ECG at the time of screening, as judged by the Investigator.
26. Current smokers or users of nicotine products. Irregular use of nicotine (e.g. smoking, snuffing, chewing tobacco) less than 3 times per week is allowed before the screening visit.
27. Investigator or any other team member involved directly or indirectly in the conduct of the clinical study.
28. Malignancy within the past 5 years or resected benign colonic polyps.
29. Regular excessive caffeine consumption defined by a daily intake of >5 cups of caffeine containing beverages.

9.6 Restrictions during the study

The subjects must be willing to comply with the following restrictions during the entire study duration *i.e.*, from screening to the Visit 6 of the SAD part and Visit 13 of the MAD part unless otherwise stated below.

9.6.1 *General restrictions*

- Contraception Requirements: The male volunteers are expected to use condom to prevent pregnancy and drug exposure of a female partner and refrain from donating sperm from the date of the first dose until 3 months after the last dose of IMP and until confirmation of negative 6 weeks skin swab and feces analyses concerning *L. reuteri* R2LC and/or pILP100 findings. In addition, they need to ensure that their fertile female partners should use contraceptive methods with a failure rate of < 1% to prevent pregnancy (for details, refer to inclusion criterion No 6). The female volunteers are expected to use a contraceptive method with a failure rate of < 1% to prevent pregnancy (for details, refer to inclusion criterion No 6). Abstinence alone is not defined as a highly effective prevention for men or WOCBP.
- Alcohol: Consumption of alcohol is not allowed within 48 hours prior to all visits to the clinic including the end-of-study visit and during visits at the research clinic.

  In addition, in the MAD, consumption of alcohol is disallowed from the first dose until the subjects leave the research clinic after the last dose.
- Nicotine: Smoking or use of nicotine-containing products is not allowed during the study (from screening to Visit 6 in the SAD part and Visit 13 in the MAD part).

- Xanthine or taurine containing products/beverages: Energy drinks (e.g. Redbull) are not allowed during the study (from screening to Visit 6 in the SAD part and Visit 13 in the MAD part).
- Coffee: Not more than 5 cups of coffee per day will be allowed during the study (from screening to Visit 6 in the SAD part and Visit 13 in the MAD part).
- Blood donation, blood components and organs: The subjects must not donate blood, plasma, other blood components or any organs during the study until 3 months after the last dose of IMP and until confirmation of negative 6 weeks skin swab and feces analyses concerning *L. reutrei* R2LC and/or pILP100 findings.
- Participation in other clinical studies: Study subjects are not allowed to participate in any other clinical study during the study period until 3 months after the last dose of IMP and until confirmation of negative 6 weeks skin swab and feces analyses concerning *L. reuteri* R2LC and/or pILP100 findings.
- Activity: study subjects will abstain from strenuous exercise for 72 hours before each blood collection for clinical laboratory tests.
- Study specific restrictions: Study subjects must refrain from any arm exercise during 14 days after the last IMP application. Shower the day after wound incision and IMP application is allowed but bathing is not allowed until 14 days after the last IMP application. Subjects are not allowed to remove the dressings or the elastic tubular bandage between visits to the research clinic. The tubular bandage should be kept for as long as the wounds need dressing (i.e. at least until Visit 6 [SAD] and Visit 13 [MAD]).

9.6.2 ***Prior and concomitant therapy***

Prohibited medication

Use of any prescribed or non-prescribed medication including antacids, analgesics, herbal remedies, vitamin supplements and minerals and other OTC drugs from 2 weeks prior to the (first) administration of IMP until Visit 6 in the SAD part and Visit 13 in the MAD part is disallowed.

Antibiotic treatment with erythromycin, clindamycin or linezolid is disallowed from 14 days prior to start of IMP administration and should be avoided until at least the 6 weeks follow-up visit in each part (Visit 7 and Visit 14, respectively) provided that the skin swab and feces samples were negative).

Importantly, use of NSAIDS (for example, but not limited to, common pain killers containing acetyl salicylic acid [e.g. Treo, Aspirin and Bamyl], diclofenac [e.g. Voltaren and Diclofenac] and ibuprofen [e.g. Ipren and Ibumetin and Ibuprofen]) are disallowed from 2 weeks prior to screening and during the whole study up until and including Visit 6 in the SAD part and Visit 13 in the MAD part.

Allowed medication

- Paracetamol in doses up to 3000 mg/day for a maximum of 4 days per week.
- Nasal decongestants without cortisone, antihistamine or anticholinergics for a maximum of 10 days.

Other medications considered necessary for the subject´s safety and wellbeing may be given at the discretion of the Investigator during the residential period. Following consultation with the Sponsor, the Investigator will determine whether or not the subject should continue in the study.

9.7 Screen failures

Screen failures are defined as subjects who consent to participate in the clinical study but are not subsequently randomized in the study. A minimal set of screen failure information is required to ensure transparent reporting of screen failure subjects. Minimal information includes documentation of signed and dated informed consent form (ICF) and reason(s) for screening failure.

Re-screening can be performed once if any of the following were reasons for screening failure or non-randomization (as judged by the Investigator):

- Practical reasons.
- No significant medical conditions (e.g. influenza, nasopharyngitis).
- Reserve subject in previous cohort.
- Plasma or blood donation outside allowed time windows.

For subjects who are re-screened, a new screening number will be assigned and a new, signed ICF will be collected.

9.8 Criteria for subject withdrawal

9.8.1 *General withdrawal criteria*

Subjects are free to discontinue their participation in the study at any time and for whatever reason without affecting their right to an appropriate follow-up investigation or their future care. If possible, the reason for discontinuation of consent should be documented.

Subjects may be withdrawn from the study at any time at the discretion of the Investigator for any of the following reasons:

- Severe non-compliance to study protocol procedures, as judged by the Investigator and/or Sponsor.
- Subject is lost to follow-up.
- Significant AEs posing a risk for the subject, as judged by the Investigator and/or Sponsor.
- Withdrawal of informed consent to the use of biological samples.
- Subject takes systemic steroids or NSAIDs within the disallowed time frame as judged by the Investigator.

9.8.2 *Procedures for discontinuation of a subject from the study*

A subject who prematurely discontinues participation in the study will always be asked about the reason(s) for discontinuation and the presence of any AEs. If a subject withdraws consent,

the investigator must ask the subject if he/she is willing, as soon as possible, to be assessed according to the procedures scheduled for Visit 6 and Visit 13 visit in each part. Any ongoing AEs will be followed as described in Section 11.7.1.14.

The primary reason for discontinuation/early withdrawal must be specified in the end-of-treatment form of the eCRF and final drug accountability must be performed.

9.8.2.1 Subject replacement

Subjects who are randomized but not dosed may be replaced as may subjects who were prematurely withdrawn from the study for any reason except the occurrence of AEs assessed at least possibly related to the IMP. Subjects withdrawn after Visit 6 (SAD part) or Visit 13 (MAD part) will not be replaced.

9.9 Randomization

The randomization in the SAD part of study will be done by arm, i.e. either active treatment to the right arm and placebo to the left arm or vice versa. Each arm will have 2 wounds which will be on same location on both arms.

The randomization in the MAD part of the study will also be done by arm, i.e. active treatment to 4 wounds to the right arm and placebo and saline to 2 wounds each on the left arm or vice versa. The wounds will be placed on the same location on both arms. For details, refer to Table 9.9-1.

A computer-generated randomization list will be created using SAS Proc Plan, SAS Version 9.4. The randomization list will be kept by the randomizer in a sealed envelope until database lock (DBL). A copy of the randomization list will be kept by an unblinded pharmacist at the site.

Sealed individual, treatment code envelopes will be kept in a locked and restricted are at the research clinic in case of need for emergency unblinding and at [redacted]

Table 9.9-1 Example of randomization (MAD)

Sequence	Location (seen from shoulder)	Treatment
2101	L1 and L2	NaCl
	L3 and L4	Placebo
	R1, R2, R3 and R4	Active
2102	L1 and L2	Placebo
	L3 and L4	NaCl
	R1, R2, R3 and R4	Active
2103	L1, L2, L3 and L4	Active
	R1 and R2	NaCl
	R3 and R4	Placebo
2104	L1, L2, L3 and L4	Active
	R1 and R2	Placebo
	R3 and R4	NaCl
Etc.		

9.10 Blinding

This is a double-blind study and the allocation of treatments will not be disclosed to clinical staff involved in study-related evaluations nor to independent evaluators until the 12 months long-term follow-up visit in each part has been conducted.

The activated ILP100-DP is a pale-yellow to yellow solution. Placebo and saline are transparent solutions. Every effort will be made at the research clinic in order to maintain the blind. Both the activated ILP100-DP, the placebo and the saline solutions will be masked in such a way that study subjects and study staff will remain blinded during the study. An unblinded pharmacist will prepare the activated ILP100-DP/placebo/saline under supervision of an unblinded study nurse or delegate. The procedure will be documented by video recording. Unblinded staff will also apply the IMP, see Section 10.6. The unblinded staff will not be involved in any study-specific evaluations (study-specific evaluations do not include sample handling). Once the adhesive film has been applied on each wound it is not possible to distinguish between the treatments.

The subjects will be blindfolded during the administration procedure.

9.11 Emergency unblinding during the study

The treatment code may only be broken by the study medical staff in case of emergency when knowledge of the treatment received is necessary for the proper medical management of the subject. The code breaking procedure should be carefully documented.

The randomization code may be broken by the iSRC during the assessment process (partial unblinding) to enable their decision on continued dosing of further cohorts or to stop the dose escalation, see Section 8.4.1. The medical staff and the subjects will still be blinded for the treatments to be administered in the subsequent cohorts in order to minimize bias (see Section 8.4.3).

For unblinding procedures in case of a potential suspected unexpected serious adverse reaction (SUSAR), refer to Section 11.7.1.13.

10 TREATMENTS

10.1 Identity of investigational medicinal products

The API is living, genetically modified *L. reuteri* R2LC bacteria transformed with a plasmid, pSIP411 with the sequence for the human chemokine CXCL12-1a inserted behind an inducible promoter, referred to as the drug substance. The drug product, ILP100-DP, is the lyophilized drug substance for topical use.

ILP100-DP is a GTMP type of ATMP classified as per Article 2 (1)(a,b) of Regulation (EC) No 1394/2007. The activated ILP100 DP is classified as a low risk microbe that and will hence be handled at BSL-1.

The **drug product ILP100-DP** will be provided in a freeze-dried formulation in a glass vial. Besides the drug product, the formulation contains ascorbic acid and sucrose. One strength of ILP100-DP will be provided: $5x10^9$ CFU/vial.

The **activator is the peptide SppIP** that induced the expression of CXCL12 by binding to the promoter in the expression vector. The activator is formulated in sterile 2.5 mM acetate buffer, pH 4.0 in a glass vial referred to as the SppIP solution.

Buffer A is used to dissolve ILP100-DP. Buffer A is a 45 mM acetate buffer, pH 5.5.

Buffer B is used to dilute ILP100-DP to 2 additional strengths. Buffer B is a 0.5% ascorbic acid w/v, 19% sucrose w/v, 60 mM phosphate, 45 mM acetate buffer, pH 4.7.

Buffer C is used to dilute the activator before use. Buffer C is a 2.5 mM acetate buffer, pH 4.0.

Buffer A, B and C are sterile and provided in separate glass vials.

The different doses of ILP100-DP will be prepared by dilutions in Buffer B. Before use, the diluted ILP100-DP will be mixed with the activation peptide SppIP to generate the ready-to-use product referred to as **activated ILP100-DP**. The activation should be done between 30 minutes and 8 hours after dilution of ILP100-DP. The activated ILP100-DP must be administered to subjects from 20 minutes to 2 hours after activation.

Placebo is Buffer B (0.5% ascorbic acid w/v, 19% sucrose w/v, 60 mM phosphate, 45 mM acetate buffer, pH 4.7) mixed with the activation peptide SppIP (SppIP solution).

The concentration of the activation peptide SppIP in the reconstituted products (activated ILP100-DP and placebo) is 100 ng/mL.

A separate IMP preparation manual detailing all steps needed to prepare activated ILP100-DP at different doses and placebo will be provided.

Sterile Saline (0.9 % NaCl) will be used as a "**no treatment**" control in the MAD part of the study. This solution will be administered in the same volume as the active treatment and placebo.

The start and stop time for the IMP application will be recorded in the eCRF (first wound and last wound).

10.2 Identity of non-investigational medicinal products

Not applicable.

10.3 Manufacturing, packaging and labelling

ILP100-DP, dilution buffers A, B and C, SppIP solution and placebo will be manufactured, packaged and released by [redacted]

The labelling of the IMP (including saline) will be performed by [redacted].

Labels will comply with applicable Good Manufacturing Practice (GMP) requirements in accordance with EudraLex Volume 4, Annex 13, 2010 and will contain information describing that the drug product is a GMO.

The labelled IMP will be shipped to the research clinic [redacted]

10.4 Conditions for storage

ILP100-DP (lyophilized powder), the SppIP solution and saline will be stored in an access-controlled refrigerator at +2°C to +8°C at [redacted]. Other solutions will be stored in an access-controlled freezer below -20°C at [redacted]

Diluted ILP100-DP must be stored at room temperature for at least 30 minutes prior to activation but may be stored at room temperature for up to 8 hours prior to activation.

Ready-to-use activated ILP100-DP and placebo must be stored at room temperature for at least 20 minutes after activation but may be stored at room temperature for up to 2 hours prior to application.

Temperature logs will be kept for the areas where the IMP is stored. The temperature should be noted on a daily basis (working days only unless automatic temperature readings are available).

10.5 Dispensing and accountability

The ready-to-use activated ILP100-DP and placebo will be prepared by a trained, unblinded pharmacist as briefly described in Section 10.1 and as detailed in a separate IMP preparation manual. A second unblinded person will oversee the preparation and dispensing process, which will be documented by video recording.

Dispensed IMP will be labelled with the following information:

- Study code
- Subject ID
- Dosing Day
- Blinded treatment ID
- Contains GMO

[redacted] and the Investigator will maintain a Storage and Accountability log and a Drug Dispensing Log detailing the dates and quantities of study medication received, dispensed to and used by each subject and study medication returned or destroyed at the end of the study. Any discrepancies between dispensed and returned IMP must be explained and documented. Products deliberately and/or accidentally destroyed by the site or the subject must be accounted for.

10.6 Treatment administration and dressing

The IMP will be administered topically on each wound by site staff in the morning of each treatment day.

Before use, diluted ILP100-DP or placebo will be mixed with the activator to generate the ready-to-use activated ILP100-DP and the placebo product, respectively, see Section 10.1 and Section 10.5. The concentration of the activation peptide SppIP in the reconstituted products will be 100 ng/ml. Detailed IMP preparation instructions will be provided separately.

The proposed dose levels in the SAD part are: $5x10^4$, $5x10^7$ and $1x10^9$ CFU/cm^2 wound area.

The proposed dose levels in the MAD part are: $5x10^5$, $5x10^7$ and $1x10^9$ CFU/cm^2 wound area.

Analysis of the dose formulation (viable count) will be performed on Day 1 (SAD and MAD, first cohort) and Day 19 (MAD, all cohorts) by [redacted]

Using a pipette, 50 µL IMP solution will be applied in the center of each wound. Two unblinded persons will take part of the administration process, one will perform the application of IMP and the other one will confirm that the correct treatment is administered to the intended wound, i.e. in accordance with the randomization list. The IMP administration will be documented by video recording. After application, each wound will be dressed with an adhesive, transparent film that will protect the wounds and isolate them from each other. Separate, non-overlapping dressings will be used for each wound. Any leakage of the treatment(s) outside the film should be commented on in the eCRF. An elastic, tubular bandage will be used on top of the films.

Following IMP administration, the wounds will be covered with adhesive, transparent film around the clock for 2 days and 2 nights (except during wound evaluations and IMP application). From Day 3 and onwards, the wounds will be treated with IMP and covered with adhesive, transparent film for 1 hour after IMP application where after the film will be removed. The wounds will be allowed to air dry and will then be separately covered with a non-occlusive dressing that can be used during showering and protects against contamination of viruses and bacteria.

At visits outlined in Table 8.1-2, the dressings will be removed and stored in -70 for future analysis (Section 11.9.3) and the area around the wound will be cleaned if necessary. Following wound photography (Section 11.5), assessment of local tolerability reactions (Section 11.7.6) and IMP application (multiple dosing in MAD only), new dressing material will be used to cover each wound. An elastic, tubular bandage will be used on top of the films.

10.7 Continuation of treatment with Investigational Medicinal Product

This is a Phase I study in healthy volunteers who will have no medical benefit from the treatment. There will be no treatment with activated ILP100 DP after end of study participation.

10.8 Treatment compliance

All IMP will be administered at the research clinic by clinical staff to ensure compliance. Any leakage of the treatment(s) outside the film should be commented on in the eCRF.

10.9 Return and destruction of investigational medicinal products

Any unused study medication will be returned to the Sponsor for destruction. Empty containers will be destroyed at the study site. The Monitor will perform final IMP accountability reconciliation at the study end to verify that all unused IMP is adequately destroyed/returned and documented.

11 STUDY ASSESSMENTS

The study assessments are described in the sections below and the timing of these assessments are detailed in the schedule of events; Table 8.1-1 and Table 8.1-2.

11.1 Recording of data

The Principal Investigator will provide the Sponsor with all data produced during the study from the scheduled study assessments. He/she ensures the accuracy, completeness, legibility, and timeliness of the data reported to Sponsor in the eCRF and in all required reports.

11.2 Procedures to mitigate and monitor environmental spread of gene modified organisms

In order to address the general environmental risk assessments on the release of GMOs into the environment highlighted in 2002:1086, [redacted] will follow general procedures for handling of high risk clinical waste in the clinic (e.g. HIV contaminated material) and also perform specific investigations on potential environmental spread of *L. reuteri* R2LC with pSIP411 in wound dressings from subjects, presence on the skin surface outside the wound area, presence in feces, presence in waste water from water locks at the research clinic.

For details regarding potential risks, their likelihood and risk management to reduce the likelihood of risks, refer to the ERA.

11.3 Demographics and other baseline characteristics

11.3.1 *Informed consent*

Signed informed consent must be obtained before any screening procedures are initiated.

The informed consent procedure is further described in Section 14.3.

11.3.2 *Eligibility criteria*

Eligibility criteria should be checked during screening and verified before randomization. The criteria are specified in Sections 9.4 and 9.5.

11.3.3 *Demographic information*

The following demographic data will be recorded: gender, age, ethnicity, race and dominant hand.

11.3.4 *Weight and height*

Weight and height will be measured without shoes. BMI will be calculated from the height and weight recorded.

11.3.5 *Medical/surgical history*

Medical/surgical history will be obtained by subject interview in order to verify that the eligibility criteria are met.

11.3.6 *Prior and concomitant medication*

Prior medication will be obtained by subject interview in order to verify that the eligibility criteria are met (see also Section 9.6.2).

Medications are classified as prior if the stop date was before or on the day of the first dose administration (pre-dose) and as concomitant if ongoing on the day of the first dose administration, stopped after the first dose administration or started after the first dose administration.

Any use of concomitant medication from screening until the last end-of-study visit must be documented in the subject's eCRF. Relevant information (*i.e.* name of medication, dose, unit, frequency, start and stop dates, reason for use) must be recorded. All changes in medication should be noted in the eCRF.

On Day 1 in each part, the time of administration of any concomitant medication, or stop of any concomitant medication, must be given in the eCRF.

11.3.7 *HIV and Hepatitis B/C*

Subjects will be tested for HIV and hepatitis B/C prior to inclusion into the study in order to protect personnel handling the blood and tissue samples.

11.3.8 *Pregnancy test*

All females will do a pregnancy test at screening (blood/serum) and at additional visits (urine) specified in Table 8.1-1 and Table 8.1-2.

11.3.9 *Urine drug screen*

Urine will be screened for drugs of abuse at visits outlined in the schedule of events (Table 8.1-1 and Table 8.1-2) using the Alere™ Drug Screen Test Panel. Additional random tests can be performed during the study period.

11.3.10 *Alcohol breath test*

An alcohol breath test will be performed at visits outlined in the schedule of events (Table 8.1-1 and Table 8.1-2). Additional random tests can be performed during the study period.

11.3.11 *Baseline symptoms*

A baseline symptom is defined as an event that occurs between subject's signing of the ICF until the first administration of IMP (i.e. an event that occurs during the screening period).

Such events are not AEs and will be recorded as baseline symptoms in the Medical History Log in the eCRF.

11.4 Wound punching/biopsy

At Visit 2 (Day 1) of each part, standardized full-thickness punch biopsy wounds (6 mm in diameter) will be produced under local anesthesia on the right and left inner arm (2 wounds per arm in the SAD part and 4 wounds per arm in the MAD part).

In the SAD part, one wound punch biopsy per subject will be saved for baseline histology analysis using the procedure described in Section 11.8.2. In addition, on Day 3 of the SAD part, approximately 48 hours after dose, punch biopsies (8 mm) from one wound per arm will be sampled under local anesthesia (injected and/or topical) and saved for histological analysis as described in Section 11.8.2.

Subjects will be treated with local anesthetics (injected and/or topical) in accordance with the manufacturer's instructions and each upper inner arm will be cleaned with 70% ethanol prior to wound incision. The wounds should be placed at least 4 cm apart. Each wound will be labelled with a permanent marker (SAD: L1 to L2 on the left arm, R1 to R2 on the right arm, MAD: L1 to L4 on the left arm and R1 to R4 on the right arm). The position of each number will be the same in all subjects). Subjects will be instructed to fill in the numbering as necessary (at least once weekly up until and including Visit 6 in the SAD part and Visit 13 in the MAD part).

Following wound incision, compression for hemostasis will be applied if necessary. In case of excessive bleeding (which is rare but may occur), where hemostasis is not achieved by compression, diathermy may be used to stop the bleeding. If diathermy is used, this should be noted in the eCRF. The subject may be treated with IMP as planned once hemostasis is achieved. There should be 1 hour (±10%) between the last wound punch and the start of IMP application to allow for pre-dose study assessments and hemostasis. For details on IMP application, refer to Section 10.6. The wound punching procedure will be detailed in a separate manual.

11.5 Wound and Scar Photography

Photographs of the wounds will be taken prior to IMP application on Day 1 and pre-treatment at all subsequent visits. Each inner arm with 2 to 4 wounds will be photographed separately (i.e. one photo of all wounds, and individual photos of each wound, on the left inner arm and one photo of all wounds on the right inner arm). A self-adhesive ruler, the subject ID, date, time and visit number must be included on each photograph. Photographs should be taken with a high-resolution camera with a macro lens and using standardized lighting (ring flash), using standardized lighting, angle and distance

Details on the camera settings and the photography procedure will be described in a separate manual.

The photographs will be evaluated by 3 to 5 independent evaluators in terms of local tolerability (Section 11.7.6.1), wound healing (Section 11.8.3), wound area (Section 11.8.4) and scar formation (Section 11.8.5).

The photographs will be uploaded in the eCRF.

11.6 Dose formulation analysis

One sample of resuspended drug product for viable count analysis will be taken at visits specified in Table 8.1-1 and Table 8.1-2. The samples will be sent for analysis to [redacted] on the same day as they were taken.

11.7 Safety assessments

11.7.1 *Adverse events*

The Principal Investigator is responsible for ensuring that all medical staff involved in the study is familiar with the content of this section and the content of the [redacted] standard operating procedures (SOPs) regarding emergencies and FIH studies.

11.7.1.1 Definition of adverse event

An AE is any untoward medical occurrence in a clinical study subject administered a pharmaceutical product and which does not necessarily have a causal relationship with this treatment. An AE can therefore be any unfavorable and unintended sign (including clinically significant abnormal values from relevant tests, such as clinical safety laboratory tests, ECGs, vital signs), symptom, or disease temporally associated with the use of an IMP, regardless of whether it is considered related to the IMP.

Local bleeding and mild, transient inflammation in the wound area are common and expected reactions following skin biopsy. Therefore, bleeding and/or mild, transient inflammation observed during the early local tolerability evaluations, and when judged by the Investigator to be caused by a study procedure performed pre-treatment (i.e. due to biopsy) is reported as procedure-related in the eCRF. Bleeding, inflammation and infection assessed as non-procedure related will be reported a AEs.

11.7.1.2 Definition of serious adverse event

An SAE is any AE that:

- results in death
- is life-threatening (this refers to an event in which the subject was at risk of death at the time of the event; it does not refer to an event that hypothetically might have caused death had it been more severe)
- requires inpatient hospitalization or prolongation of existing hospitalization
- results in persistent or significant disability/incapacity
- is a congenital anomaly/birth defect
- is medically important (this refers to an event that may not be immediately life-threatening or result in death or hospitalization, but may jeopardize the subject or may require intervention to prevent any of the SAEs defined above)

Examples of medically important events are intensive treatment in an emergency room for allergic bronchospasm or blood dyscrasias, convulsions that do not result in hospitalization, development of drug dependency, and drug abuse.

Planned hospitalizations or surgical interventions for a condition that existed before the subject signed the ICF and that did not change in intensity are not SAEs.

If there is any doubt as to whether an AE meets the definition of an SAE, a conservative viewpoint must be taken, and the AE must be reported as an SAE.

11.7.1.3 Definition of adverse drug reaction

The term adverse drug reaction (ADR) is to be used whenever either the Investigator or Sponsor or designee assessed the SAE as at least possibly related to the IMP.

11.7.1.4 Definition of serious adverse drug reaction

The term SADR is to be used whenever either the Investigator or Sponsor or designee assessed the SAE as at least possibly related to the IMP.

11.7.1.5 Definition of suspected unexpected serious adverse reaction

A SUSAR is any SADR whose nature or intensity is not consistent with the current version of the IB.

11.7.1.6 Time period and frequency for collecting adverse events

AEs (including SAEs) will be collected from the start of IMP administration until the end-of-study visit. From Visit 7 and 14 in the SAD and MAD part, respectively, pre-defined AEs will be asked for.

Any AE with start date on the day of first IMP administration must be recorded with start time.

At the end-of-study visit, information on new AEs, if any, and stop dates for previously reported AEs must be recorded (if known).

Investigators are not obligated to actively seek AE or SAE after conclusion of the study participation. However, if the investigator learns of any SAE, including a death, at any time after a participant has been discharged from the study, and he/she considers the event to be reasonably related to the study intervention or study participation, the investigator must promptly notify the sponsor.

11.7.1.7 Assessment of severity/intensity

The grading of the severity/intensity of AEs will follow the CTCAE v5.0. Grade refers to the severity of the AE. The CTCAE displays Grades 1 through 5 with unique clinical descriptions of severity for each AE based on this general guideline.

The Investigator must assess the severity/intensity of an AE using the following definitions, and record it on the AE Log in the eCRF:

Grade 1	Mild; asymptomatic or mild symptoms; clinical or diagnostic observations only; intervention not indicated.
Grade 2	Moderate; minimal, local or non-invasive intervention indicated; limiting age-appropriate instrumental activities of daily living (ADL)*.

Grade 3	Severe or medically significant but not immediately life-threatening; hospitalization or prolongation of hospitalization indicated; disabling; limiting self- care ADL**.
Grade 4	Life-threatening consequences; urgent intervention indicated.
Grade 5	Death related to AE.

**Instrumental ADL refers to preparing meals, shopping for groceries or clothes, using the telephone, managing money, etc.*

***Self- care ADL refers to bathing, dressing and undressing, feeding self, using the toilet, taking medications, and not bedridden.*

11.7.1.8 Assessment of causal relationship

The Investigator must assess the causal relationship between an AE and the IMP using the definitions below and record it in the AE Log of the eCRF:

- *Unlikely* – the AE has no temporal relationship to the IMP or is due to underlying/concurrent illness or effect of another drug (that is, there is no causal relationship between the IMP and the AE).
- *Possible* – the AE has a suggestive temporal relationship to the IMP, and an alternative etiology is equally or less likely
- *Probable* – the AE has a strong temporal relationship to the IMP or recurs on re-challenge, and another etiology is unlikely or significantly less likely

An AE is considered causally related to the use of the IMP when the causality assessment is *probable* or *possible*.

11.7.1.9 Assessment of outcome

The Investigator must assess the outcome of an AE using the definitions below and record it on the AE Log of the eCRF:

- *Recovered/Resolved* – the subject has recovered completely, and no symptoms remain.
- *Recovering/resolving* – the subject's condition is improving, but symptoms still remain.
- *Recovered/resolved with sequelae* – the subject has recovered, but some symptoms remain (for example, the subject had a stroke and is functioning normally, but has some motor impairment).
- *Not recovered/not resolved* – the subject's condition has not improved, and the symptoms are unchanged (for example, an atrial fibrillation has become chronic).
- *Fatal*
- *Unknown*

11.7.1.10 Collecting adverse events

AEs identified using any of the following methods will be recorded:

- AEs spontaneously reported by the subject
- AEs observed by the Investigator or medical personnel
- AEs elicited based on non-leading questions from the Investigator or medical personnel

11.7.1.11 Recording adverse events

AEs must be recorded in the AE Log of the eCRF. The investigator must provide information on the AE, preferably with a diagnosis or at least with signs and symptoms; start and stop dates, start and stop time; intensity; causal relationship to IMP; action taken, and outcome.

If the AE is serious, this must be indicated in the eCRF (see Section 11.7.1.12).

AEs, including out-of-range clinically significant clinical safety laboratory values, must be recorded individually, except when considered manifestations of the same medical condition or disease state; in such cases, they must be recorded under a single diagnosis.

If the severity/intensity of an AE increases, the maximum intensity is recorded in the eCRF.

11.7.1.12 Reporting of serious adverse events

SAE reporting should be performed by the investigator within 24 hours of awareness via the eCRF. All available information regarding the SAE should be entered in the AE Log for the specific subject. By saving the event as "serious" in the eCRF, an e-mail alert is sent to predefined recipients to highlight that an SAE has been registered. The same information is automatically sent to [redacted]

The SAE report is reviewed by a designated person at [redacted] PV department to ensure that the report is valid and correct. For fatal or life-threatening SAEs where important or relevant information is missing, immediate follow-up is undertaken and queries to the site are raised. Investigators or other site personnel should inform [redacted] PV of any follow-up information on a previously reported SAE immediately but no later than the end of the next business day of when he or she becomes aware of it.

If the SAE report in the eCRF is updated, a new e-mail alert is sent to the predefined recipients.

The appointed Medical Monitor will provide his/her causality assessment and make an expectedness assessment once the report is judged to be complete. The reference document for definition of expectedness is the Reference Safety Information (RSI) in the current version of IB.

If any additional documentation is required (e.g. autopsy report), [redacted] PV will request this information from the study site.

In case the eCRF cannot be accessed, the SAE should be reported by manually completing the paper SAE Form, provided in the Investigator Site File (ISF). The completed, signed and dated paper SAE Form should, within 24 hours, be scanned or e-mailed to:

[redacted]

A copy of the paper SAE form must also be e-mailed to [redacted]

The study site should notify the site Monitor via phone or e-mail about the submission of the SAE report. As soon as the site personnel have access to [redacted] the SAE should be reported electronically as well.

The Sponsor has delegated to [redacted] the reporting of SAEs to competent authority (CA) and the independent ethics committee (IEC) in accordance with local regulations.

11.7.1.13 Reporting of SUSARs to EudraVigilance, local CA and IEC

The term SADR is used whenever either the investigator or medical monitor deems a blinded SAE as possibly or probably related to IMP. If an SADR is assessed as unexpected by the medical monitor, it is a potential SUSAR and under such circumstances an EudraVigilance reporter will be unblinded. In case the subject had received active treatment, the event is regarded as a SUSAR and the certified EudraVigilance reporter will report the SUSAR to the CA, via the EudraVigilance database, and to the IEC in accordance with local regulations and [redacted] SOPs within the following timelines:

- 7 calendar days if fatal or life-threatening (follow-up information within an additional 8 days)
- 15 calendar days if non-fatal and non-life-threatening (follow-up information as soon as possible)

The clock for expedited initial reporting (Day 0) starts as soon as the Sponsor has received the information containing the minimum reporting criteria. The date should be documented on an acknowledgement of receipt.

The medical monitor is responsible for medical review of the SAE narrative in the Council for International Organizations of Medical Sciences (CIOMS) form (or equivalent) prior to expedited reporting.

The Sponsor or delegate is responsible for informing the Investigators concerned of relevant information about potential SUSARs (blinded data) that could adversely affect the safety of subjects.

The Sponsor or delegate is responsible for once a year throughout the clinical study (or on request), submit a safety report to the CA and the IEC taking into account all new available safety information received during the reporting period.

11.7.1.14 Treatment and follow-up of adverse events

Subjects with AEs that occur during the study must be treated according to daily clinical practice at the discretion of the Investigator.

AEs must be followed up until resolution or to the end-of-study visit, whichever comes first. At the end-of-study visit, information on new AEs, if any, and stop dates for previously reported AEs must be recorded (if known). AEs assessed as stable by the Investigator at the end-of-study visit will not have to be followed up until resolution.

It is the responsibility of the Investigator to follow up on all SAEs until the subject has recovered, stabilized, or recovered with sequelae, and to report to the Sponsor all relevant new information using the same procedures and timelines as those for the initial report. Relevant information includes discharge summaries, autopsy reports, and medical consultation.

11.7.1.15 Procedures in case of pregnancy

In case of pregnancy or suspicion of possible pregnancy of any female partners of male subjects, the study treatment must be stopped immediately, and the subject discontinued from participation in the study. Pregnancy itself is not regarded as an AE unless there is a suspicion that the IMP may have interfered with the effectiveness of the contraceptive medication. However, the outcome of all pregnancies (spontaneous miscarriage, elective termination, normal birth or congenital abnormality) must be followed up and documented even after the subject was discontinued from the study.

All events of congenital abnormalities/birth defects are SAEs. Spontaneous miscarriages should also be reported and handled as AEs. All outcomes of pregnancy must be reported to the Sponsor and the Principal Investigator on the pregnancy outcomes report form.

11.7.1.16 Treatment of overdose

An overdose is a dose in excess of the dose specified for each cohort in this CSP.

Overdosing is not likely to occur since all IMP will be administered by site personnel under medical surveillance. In cases of accidental overdose, standard supportive measures should be adopted as required.

An overdose should be documented as follows:

- An overdose with associated AE is recorded as the AE diagnosis/symptoms in the AE Log of the eCRF.
- An overdose without associated symptoms is only reported in the subject´s medical records.

No known antidote is available.

11.7.2 *Physical examination*

The physical examination will include assessments of skin (arms), lungs, cardiac, abdomen (liver and spleen).

11.7.3 *Vital signs*

Systolic and diastolic blood pressure and pulse will be measured in supine position after 10 minutes of rest. Body temperature will be measured orally using a digital thermometer.

11.7.4 *Resting 12-lead ECG*

Single 12-lead ECG will be recorded in supine position after 10 minutes of rest using an ECG machine. Heart rate and PQ/PR, QRS, QT and QTcF intervals will be recorded.

Safety ECGs will be reviewed and interpreted on-site by the Investigator.

11.7.5 *Laboratory safety assessments*

Blood samples for analysis of clinical chemistry, hematology and coagulation parameters will be collected through venipuncture or an indwelling venous catheter and sent to the certified clinical chemistry laboratory at Uppsala University Hospital and analyzed by routine analytical methods.

Urine analysis will be performed at the research clinic using dip sticks.

The following safety laboratory parameters will be assessed:

Clinical Chemistry

Alanine aminotransferase (ALT)

Alkaline phosphatase (ALP)

Albumin

Aspartate aminotransferase (AST)

Bilirubin (total)

Calcium

Chloride

Creatinine

Gamma-glutamyl transferase (GGT)

Glucose

Magnesium

Phosphate

Potassium

Sodium

Urea nitrogen

Coagulation

Activated Partial Thromboplastin Time (APTT)

Prothrombin Complex International Normalized Ratio (PK[INR])

Hematology

Hematocrit

Hemoglobin (Hb)

Platelet count

Red blood cell (RBC) count

White blood cell (WBC) count w differential count

Urinalysis (dip stick)

Specific gravity

pH

Leucocytes

Nitrite

Protein

Glucose

Ketones

Urobilinogen

Bilirubin

Erythrocytes

11.7.6 *Evaluation of local tolerability*

11.7.6.1 Evaluation of local tolerability by the Investigator and by independent evaluators

The investigator and 3 to 5 independent evaluators will assess local tolerability reactions of each wound by direct inspection or by evaluation of photographs, respectively, at visits specified in Table 8.1-1 and Table 8.1-2. The following parameters will be evaluated per wound:

- Appearance of wound and wound edge (inflammation)
- Condition of skin surrounding the wound (inflammation)
- Hemorrhage
- Amount of exudate present
- Presence of slough/necrotic tissue
- Presence of granulation tissue
- Hypergranulation

Each parameter will be scored using a 4-graded scale (0-3). Details are provided in a separate document.

Each wound will be separately evaluated and the outcome will be documented in the eCRF.

Hemorrhage, but not coagulated blood in wounds, evident after IMP administration will be reported as an AE. Hemorrhage/bleeding apparent at several visits will be reported as intermittent bleeding.

Exceptional and persistent inflammation associated with the wounds will be reported as an AE as will any wound infection.

Other local tolerability parameters assessed by the Investigator and by independent evaluators will not be reported as AE.

11.7.6.2 Evaluation of local tolerability by the subjects

The subjects will be asked to assess pruritus and pain on a 4-graded scale (0-3) of each wound.

Each wound will be separately evaluated and the outcome will be documented in the eCRF.

Persistent pain and pruritus reported by a subject may be reported as an AE, as judged by the Investigator.

11.7.7 *Wound infections*

The Investigator will assess the wounds for any signs of infection such as smell or pus formation. Infections will be registered per wound in the local tolerability module of the eCRF as well as in the AE Log of the eCRF.

11.7.8 *Assessment of CXCL12 anti-drug antibody formation*

Venous blood samples for the assessment of potential ADAs developed against CXCL12, will be collected through venipuncture or an indwelling venous catheter before the first administration and at visits outlined in Table 8.1-1 and Table 8.1-2.

The date and time of collection of each sample will be recorded in the eCRF.

The blood samples will be collected in pre-labelled K2-EDTA tubes and will be centrifuged at +4°C at 3000g for 15 minutes. The separated plasma from each sample will be divided into 2 aliquots in pre-labelled polypropylene cryotubes and frozen immediately at -70°C.

The samples will be analyzed by [redacted], by means of a validated ELISA or AlphaLisa method.

11.8 Assessments related to secondary endpoints

11.8.1 *Blood sampling for analysis of systemic exposure of CXCL12 in plasma and L. reuteri in blood*

Venous blood samples (approximately 5 mL) for the determination of plasma concentrations of CXCL12 (Section 11.8.1.1), for blood culturing (Section 11.8.1.2) for analysis of *L. reuteri* in blood, for future exploratory analyses (11.8.1.1) and for future PBMC analysis (Section 11.8.1.3), will be collected through venipuncture or an indwelling venous catheter at pre-specified visits (see Table 8.1-1 and Table 8.1-2). The blood samples will be collected in pre-labelled EDTA-K2 tubes.

The date and time of collection of each sample will be recorded in the eCRF.

On Day 3 and 14 of the SAD part (Visit 4 and Visit 5), and on Day 3, 5, 8, 15 and 21 of the MAD part (Visits 4, 5, 6, 9 and 12), 200 µL of the venous blood sample should be plated for blood culturing prior to centrifugation for plasma collection as described in Section 11.8.1.2.

11.8.1.1 Plasma collection for CXCL12 analysis

The collected samples should be placed on ice until centrifuged. Centrifugation will be performed at 4°C at 3000g for 15 minutes. The separated plasma from each blood sample will be kept on dry ice, divided into 3 aliquots in pre-labelled polypropylene Nunc cryotubes (polypropylene tubes with internal thread, Thermo Scientific, Denmark; A, B and C samples, 500 µL in each tube) and frozen at -70°C within 1 hour after collection. The A sample will be sent for analysis, the B sample will be kept as back-up and the C sample will be saved for potential future analyses of e.g. IgGs or levels of proteins associated with systemic inflammation or immune response.

The analysis will measure endogenous and bacterially-derived CXCL12 in the plasma samples using a validated method based on ELISA technology. The analysis will be performed by [redacted].

11.8.1.2 Blood culturing for analysis of L. reuteri in blood

A total of 200 µL of the venous blood sample (Section 11.8.1) will be transferred to 2 MRS +erm/vanco plates (100 µL to each plate) prior to centrifugation. Sterile glass balls (4 to 8) will be added and the blood will spread on the plates by gently shaking the plates back and

forth for 1-2 minutes. The plating must be done **before** centrifugation of the blood samples for collection of plasma.

The plates will be allowed to rest in room temperature with closed lids until all blood samples for the day has been taken. All plates will be placed in a jar, which in turn will be placed in an activated anaerobic bag that should be immediately closed. The jar will be placed in a 37 ºC (±2 ºC) incubator for 48 hours. After 48 hours the plates will be taken out of the jars and inspected for colonies.

Presence of absence of any colonies must be documented in the eCRF. In case of any colonies, the number of colonies per plate will be documented in the eCRF and the plate will be sent to [redacted] for subsequent evaluation. The colonies will be analyzed by [redacted] for viable *L. reuteri* R2LC containing the pSIP411 plasmid.

The data will be delivered as an external file.

11.8.1.3 PBMC analysis

Following centrifugation of the venous blood samples collected pre-dose on Day 1 and pre-dose on Day 19, and after removal of the plasma, the remaining fraction should be mixed with 2 mL 30% glycerol and frozen at -70°C.

11.8.2 ***Biopsy for analysis of local exposure of CXCL12 in the wound, immunohistochemistry, and histopathology (SAD part only)***

On Day 1, one biopsy per subject generated during the wound incision process (Section 11.4) will be saved for baseline histology analysis. In addition, on Day 3, approximately 48 hours after dose, punch biopsies (8 mm) from one wound per arm will be sampled under local anesthesia (injected and/or topical). Following biopsies, compression for hemostasis will be applied if necessary. The residual 2 biopsy wounds will be closed using 1-2 surgical sutures (Prolene®, polypropylene) and dressed as appropriate. The other 2 wounds will be left untouched for continued analysis. For removal of the sutures, subjects may be referred to a local district health care center.

Half the biopsy sample will be snap frozen on dry ice, half will be fixed in 4% formalin.

Details on the procedure will be specified in a separate manual.

The data will be delivered in external files.

11.8.3 ***Assessment of wound healing and wound rupture***

The Investigator and the independent evaluators will assess the wound healing status of each wound by direct inspection or by evaluation of photographs, respectively, at visits specified in Table 8.1-1 and Table 8.1-2.

Wound healing, i.e. complete wound closure, is defined as skin re-epithelialization without dressing requirements.

The results of the assessment will be reported in the eCRF as “Yes” or “No” per wound. At each post-wound heling visit, it should be documented if the wound is still healed or if any rupture(s) has occurred.

11.8.4 *Wound area measurements*

The independent evaluators will perform wound area measurements of each wound based on photographs using the Image J software (National Institutes of Health [NIH]) or equivalent at visits specified in (Table 8.1-1 and Table 8.1-2).

11.8.5 *Evaluation of scar tissue formation*

An overall assessment of scar formation (normal/abnormal per wound) will be performed by the Investigator and by independent evaluators at visits specified in Table 8.1-1 and Table 8.1-2. The outcome will be documented per wound/scar in the eCRF.

11.8.5.1 Vancouver scar scale

The Burn Scar Index, or Vancouver Scar Scale, is widely used in clinical practice and research to document change in scar appearance over time. The scale scores the scar on four parameters: pigmentation, vascularity, pliability and height. Each scar will be evaluated by the Investigator and by independent evaluators as detailed in Table 11.8-1

Table 11.8-1 Vancouver Scar Scale

Pliability (P)	Height (H)	Vascularity (V)	Pigmentation (M)
0: normal	0: normal	0: normal	0: normal
1: supple	1: 1~2 mm	1: pink	1: hypopigmented
2: yielding	2: 3~4 mm	2: red	2: mixed
3: firm	3: 5~6 mm	3: purple	3: hyperpigmented
4: adherent	4: > 6 mm		

11.8.6 Assessment of *L. reuteri* colonies on the skin surrounding the wounds

At visits specified in Table 8.1-1 and Table 8.1-2, swab samples will be taken from the skin surrounding each wound using sterile swabs (MW176PF Transwab, 40 Compliant). The swab will be taken approximately 1 cm from the wound edge, **before** wound examination and cleaning of the skin.

The swab will be placed back into the sterile container, covered with 30 % sterile glycerol, 2 mL, and stored at -70°C until shipped to [redacted] on dry ice.

The samples will be analyzed by [redacted] for viable *L. reuteri* R2LC containing the pSIP411 plasmid using a method of culturing conditions specific to detect *L. reuteri* R2LC; PCR and sequencing.

The data will be delivered in an external file.

11.8.7 Assessment of presence of *L. reuteri* in feces

Feces samples will be collected using a feces kit before the first treatment and at visits specified in Table 8.1-1 and Table 8.1-2. A separate fecal sampling instruction manual will be provided to the subjects.

The samples will be shipped to [redacted] and analyzed for viable *L. reuteri* R2LC containing the pSIP411 plasmid using a method of culturing conditions specific to detect *L. reuteri* R2LC, PCR and sequencing.

The data will be delivered in an external file.

11.9 Assessments related to exploratory endpoints

11.9.1 *3D Imaging*

Each wound will be analyzed using a 3D Camera (Cherry Imaging). The wound/scar margin will be outlined using the Image J software (NIH) or equivalent and subsequently analyzed using the Trace software (Cherry Imaging).

Prior to scanning, an assessment of skin type will be performed, which will be documented in the eCRF.

During the scanning process, many small images are acquired. Together the images are merged by the algorithm into a high-resolution stitched 3D model. The models generated by the software are aligned, which enables measurements. The following parameters will subsequently be measured: wound volume, wound area, wound skin pigmentation, scar volume, scar area, scar skin pigmentation and evenness/roughness of the scar.

The data will be delivered in an external file.

11.9.2 *Microcirculation*

To understand more subtle tolerability affects as well as the impact of the activated ILP100-DP on the immediate skin, microcirculation in the wounds and adjacent skin will be recorded using Laser Speckle Contrast Analysis (LASCA) where blood flow is recorded in an area of 5x10 cm around 2 wound at a time during at least 2 minutes. During this time the subject have to hold the arm still. Data will be recorded at visits specified in Table 8.1-2.

The data will be delivered as an external file. The outcome of the analysis may not be reported in the CSR.

11.9.3 *Dressing collection for microbiome analysis*

The adhesive, transparent film will be removed from the wound and transferred to and Eppendorf tube or similar. The tube will be placed on dry ice until transferred to a -70°C freezer. Samples will be analyzed for microflora using Illumina or equivalent by an independent contractor.

Dressings will be collected at visits specified in Table 8.1-2 and will be snap frozen and saved at -70°C for future, exploratory microbiome analyses. The analyses are not included in this study.

The outcome of the analysis may not be reported in the CSR.

11.10 Appropriateness of measurements

The stopping rules for dose escalation used (see Section 8.4.1) follows the recommendations and grading system of CTCAE v5.0 but also take into account the recommendations

published by Sibille *et al.*, 2010 which is an adaptation to FIH studies of the grading systems previously proposed by NCI, WHO, NIH and FDA.

All other methods used for safety assessments are commonly used in standard medical care and in Phase I clinical studies.

12 PROCEDURES FOR BIOLOGICAL SAMPLES

12.1 Sample collection

The sample collection procedures for CXCL12 analysis in blood and biopsies are described in Section 11.8.1 and Section 11.8.2.

The sample collection procedure for ADA analysis is described in Section 11.7.8.

Collection of skins swabs, feces samples and dressings for microbiome analysis are described in Section 11.8.6, Section 11.8.7 and Section 11.9.3.

Safety laboratory samples are collected according to standard procedures.

12.2 Volume of blood

The maximum volume of blood collected from each subject will not exceed 150 mL in the SAD part or 200 mL in the MAD part, which is less than half of the 450 mL blood drawn during a regular blood donation.

12.3 Handling, storage and destruction of laboratory samples

All biological samples will be registered in biobank [redacted]

Any remains from the safety laboratory samples will be disposed of after analyses.

The plasma samples for analyses of CXCL12 will be stored at -70°C until analyzed. The samples will be disposed once the final CSR has been finalized.

Samples for potential PBMC analysis, biopsies, left-over back-up plasma samples and plasma samples saved for potential future analyses of e.g. IgGs or levels of proteins associated with systemic inflammation or immune response will be transferred to the Sponsor's biobank, or another biobank, following Visit 6 (SAD) and 13 (MAD) and may be retained for up to 10 years after study completion for future analysis.

12.4 Chain of custody of biological samples

A full chain of custody is maintained for all samples throughout their lifecycle.

[redacted] keeps full traceability of collected biological samples from the subjects while in storage at the research clinic until shipment and keeps documentation of receipt of arrival.

The sample receiver (the analytical laboratory) keeps full traceability of the samples while in their storage and during use until used or disposed of.

The Sponsor keeps oversight of the entire life cycle through internal procedures, monitoring of study sites and auditing of external laboratory providers.

12.5 Withdrawal of informed consent for donated biological samples

If a subject withdraws consent to the use of biological samples donated, the samples will be disposed of /destroyed, if not already analyzed and documented.

The Principal Investigator will ensure that:

- Subject withdrawal of informed consent is notified immediately to Sponsor.
- Biological samples from the subject, if stored at the research clinic, are immediately identified, disposed of/destroyed and the action is documented.

The Sponsor has to ensure that the laboratory(ies) holding the samples is/are informed about the withdrawn consent immediately and that samples are disposed of/destroyed or returned to the research clinic and the action is documented.

13 QUALITY MANAGEMENT, QUALITY ASSURANCE AND QUALITY CONTROL

13.1 Critical process, system and data identification

During protocol development, the sponsor will identify those processes, systems (facilities, computerized systems) and data that are critical to ensure human subject protection and the reliability of trial results according to applicable SOPs and International Conference on Harmonization (ICH) E6 R2.

Identified risks will be categorized separately from the CSP.

13.2 Quality assurance and quality control

The sponsor is responsible for implementing and maintaining quality assurance and quality control (QC) systems with written SOPs with regards to management of identified risks, CSP compliance, good clinical practice (GCP) compliance and applicable regulatory requirements.

The sponsor is responsible for securing agreements with involved subcontractors and to perform regular subcontractor oversight to ensure CSP compliance, GCP compliance and compliance with applicable regulatory requirements.

The Sponsor is responsible for implementing a risk-based validated EDC system and maintain SOPs for the whole life- cycle of the system.

QC should be applied to each stage of data handling to ensure that all data are reliable and have been processed correctly.

14 ETHICAL AND REGULATORY REQUIREMENTS

14.1 Ethical conduct of the study

The study will be performed in accordance with ethical principles that have their origin in the Declaration of Helsinki and are consistent with ICH/GCP E6 (R2), EU Clinical Trials Directive, and applicable local regulatory requirements.

A link to the Declaration of Helsinki is included in Appendix 19.2.

14.2 Ethics and regulatory review

The Principal Investigator is responsible for submission of the CSP, the subject information and ICF, any other written information to be provided to the subjects and any advertisements used for recruitment of subjects to applicable IEC for approval.

The Sponsor has delegated to [redacted] the responsibility for submission of study documents to the applicable CA according to local regulatory requirements.

Approval must be obtained in writing from both IEC and CA before the first subject can be recruited.

The Sponsor will provide the CA, IEC and Principal Investigators with safety updates/reports according to local requirements. Progress reports and notifications of SUSARs will be provided to the IEC according to local regulations and guidelines.

14.3 Subject information and consent

It is the responsibility of the Investigator or an authorized associate to give each potential study subject adequate verbal and written information before any study specific assessments are performed.

The information will include the objectives and the procedures of the study as well as any risks or inconvenience involved. It will be emphasized that participation in the study is voluntary and that the subject may withdraw from participation at any time and for any reason, without any prejudice. All subjects will be given the opportunity to ask questions about the study and will be given sufficient time to consider participation before signing the ICF.

Before performing any study-related procedures the ICF must be signed and personally dated by the subject and by the Investigator. A copy of the subject information including the signed ICF will be provided to the subject.

Documentation of the discussion and the date of informed consent must be recorded in the source documentation and in the eCRF. The subject information sheet and the signed ICF should be filed by the Investigator for possible future audits and/or inspections.

The final approved version of the subject information and ICF must not be changed without approval from the Sponsor and the applicable IEC.

14.4 Subject information card

The subject will be provided with a Subject information card including the following information:

- That he/she is participating in a clinical study
- Subject study ID
- That he/she is treated with the IMP
- That he/she is treated with a GMO
- The name and phone number of the Investigator
- Name and address of the Sponsor

14.5 Subject data protection

The ICF includes information that data will be recorded, collected and processed and may be transferred to European Economic Area (EEA) or non-EEA countries. In accordance with the European Union Data Protection Directive (95/46/EC) and General Data Protection Regulation (GDPR), the data will not identify any persons taking part in the study.

The potential study subject should be informed that by signing the ICF he/she approves that authorized representatives from Sponsor and [redacted], the concerned IEC and CA have direct access to his/her medical records for verification of clinical study procedures. This agreement is to be substantiated in a separate document, according to local requirements.

The subject has the right to request access to his/her personal data and the right to request rectification of any data that is not correct and/or complete in accordance with the European Union Data Protection Directive (95/46/EC) and the request will be raised to the Principal Investigator.

The Investigator must file a Subject Identification List, which includes sufficient information to link records, i.e. the eCRF and clinical records. This list should be preserved for possible future inspections/audits but must not be made available to the Sponsor except for monitoring or auditing purposes.

Personal data that are collected in the study such as health information and ethnicity are considered as sensitive personal data. This data will be pseudoanonymized, i.e. personally identifiable information (PII) will be removed and replaced by a unique subject ID and will be processed by the Sponsor and other involved parties during the study. After the study end, only anonymized data, i.e. aggregated data sets, can be used.

For this study, the Sponsor Ilya Pharma AB is the data controller of all data processed during the study (e.g. trial master file [TMF], study reports) and [redacted] is the data processor. Any subcontractors used in the study (see Section 5), are also data processors.

For data that are processed at the research clinic (e.g. medical records and ISF), [redacted] is the data controller.

14.6 Changes to the approved clinical study protocol

Any proposed change to the approved Final CSP (including appendices) will be documented in a written and numbered clinical protocol amendment. All substantial amendments to the

protocol must be approved by the appropriate IEC and/or CA before implementation according to applicable regulations.

14.7 Audits and inspections

Authorized representatives of Sponsor, a CA, or an IEC may perform audits or inspections at the research clinic, including source data verification (SDV). The purpose of an audit or inspection is to systematically and independently examine all study-related activities and documents, to determine whether these activities were conducted, and data were recorded, analyzed, and accurately reported according to the protocol, ICH-GCP guidelines and any applicable regulatory requirements. The Investigator will contact the Sponsor immediately if contacted by a CA about an inspection at the center.

14.8 Insurance

Subjects will be covered under Ilya Pharma AB:s liability insurance policy through the Swedish Pharmaceutical Insurance (Läkemedelsförsäkringen). The certificate of insurance and an information leaflet containing essential information about the insurance coverage can be provided upon request. The participating subjects are also protected in accordance with national regulations, as applicable. [redacted] has a company insurance covering services performed by [redacted].

15 STUDY MANAGEMENT

15.1 Training of study site personnel

Before enrolment of the first study subject a Sponsor representative or delegate will perform a study initiation visit at the research clinic. The requirements of the CSP and related documents will be reviewed and discussed, and the investigational staff will be trained in any study specific procedures and system(s) utilized.

It is the responsibility of the Investigator to ensure that all personnel involved in the study are fully informed of all relevant aspects of the study and have a detailed knowledge of and training in the procedures that are to be executed by them. Any new information of relevance to the performance of this study must be forwarded to the staff involved in a timely manner.

The Investigator will keep a list of all personnel involved in the study together with their function and study related duties delegated. A Curriculum Vitae will be available for all staff delegated study-specific duties.

15.2 Clinical monitoring

The Sponsor is responsible for securing agreement from all involved parties to ensure direct access to all study related sites, source data/documents, and reports for the purpose of monitoring and auditing by the sponsor, and inspection by domestic and foreign regulatory authorities.

As defined in the risk-based monitoring (RBM) plan, approved by the sponsor and provided separately, the responsible Monitor will periodically visit the study site at times agreed upon

by the Investigator and the Monitor. At the time of each monitoring visit, the role of the Monitor is (but not limited to) to:

- Provide information and support to the investigational team.
- Confirm that facilities and resources remain acceptable.
- Confirm that the investigational team is adhering to the CSP, applicable SOPs, guidelines, manuals and regulatory requirements.
- Verify that data are being accurately and timely recorded in the eCRF and that IMP accountability checks are being performed.
- Verify that data in the eCRF are consistent with the clinical records (SDV) in accordance with the Monitoring Plan.
- Verify that the correct informed consent procedure has been adhered to for participating subjects.
- Ensure that withdrawal of informed consent to the use of the subject's biological samples will be reported and biological samples are identified and disposed of/destructed accordingly, and that this action is documented and reported to the subject.
- Verify that AEs are recorded and reported in a timely manner and according to the CSP.
- Raise and escalate any serious quality issues, serious GCP breach and any data privacy breach to the Sponsor.

Centralized monitoring will also be performed continuously by study team members by [redacted] in accordance with the RBM plan.

When the study has been completed and all queries have been resolved and the database has been locked, the Monitor will perform a close-out visit.

15.3 Medical Monitoring

The Medical Monitor is a physician who acts as the Sponsor's safety representative and is qualified for medical monitoring according to regulatory requirements. Qualification should be evident by relevant pharmacovigilance training including safety assessments for safety reporting.

The responsibilities of the Medical Monitor will be specified in the Safety Management Plan.

15.4 Source data documents

A separate Origin of Source Data List will be generated for each site before start of enrolment, specifying the location of the source of derived information appearing in the eCRF. This document must be signed by the Principal Investigator and the Monitor to confirm agreement before start of recruitment.

Source documents are all documents used by the Investigator or hospital that relate to the subject's medical history, that verifies the existence of the subject, the inclusion and exclusion criteria, and all records covering the subject's participation in the trial. They include laboratory notes, memoranda, material dispensing records, subject files, etc. The eCRF may constitute source data if clearly defined in the Origin of Source Data List.

The Investigator should guarantee access to source documents to the Monitor, CAs and the IECs, if required.

15.5 Study agreements

The Principal Investigator must comply with all the terms, conditions, and obligations of the Clinical Study Agreement for this study.

Agreements between Sponsor and [redacted] must be in place before any study-related procedures can take place, or subjects be enrolled.

15.6 Study time table and end of study

The study is expected to start in Q3, 2019 and the treatment phase is expected to be completed by Q3, 2020 followed by a 5-year follow-up period.

A subject is considered to have completed the treatment phase of the study if he/she has completed all visits up until and including Visit 6 of the SAD part and Visit 13 of the MAD part. A subject is considered to have completed the long-term follow up if he/she has completed all visits up until and including the end-of-study visit.

The end of the treatment phase is defined as the date of Visit 6 or 13 of the last subject in the SAD and MAD part, respectively. The end of the study is defined as the date of the last visit of the last subject in the study.

15.7 Discontinuation of the study

The Sponsor reserves the right to discontinue the study at any time but intends only to exercise this right for valid scientific or administrative reasons.

After such a decision, the Investigator must inform all participating subjects and perform relevant assessments, preferably according to the scheme for the final assessments. All delivered and unused study products and other study materials must be returned and all eCRFs completed as far as possible.

15.8 Reporting and publication

15.8.1 *Clinical study report*

A summarizing report must be submitted to the applicable CA and IEC within 12 months after completion of the study (in accordance with LVFS 2011:19, Chapter 9). The study results will be reported in the EudraCT database per applicable regulations within 12 months after completion of the study.

Interim reports based on unblinded data will be prepared after completion of the 6 weeks visit (Visit 7 [SAD part] and Visit 14 [MAD part] and after completion of the 12 months visits (Visit 10 [SAD part] and Visit 17 [MAD part]; one report with combined 12 months SAD and MAD data). The data for the reports will be produced from exports of the cleaned database

but no formal DBL will be performed until after the last subject has performed the last 5-year follow-up assessment. A complete CSR including will be written following the final DBL.

Data obtained from any exploratory analyses may be reported separately.

15.8.2 *Annual safety report*

The Sponsor will submit development safety update report (DSUR) to the CA and to the IEC. The report shall summarize all pertinent safety information collected during the reporting period and contain an update of the risk-benefit evaluation if there has been any change since the approval of the clinical study.

15.8.3 *Confidentiality and ownership of study data*

Any confidential information relating to the IMP or the study, including any data and results from the study, will be the exclusive property of the Sponsor. The Investigator and any other persons involved in the study are responsible for protecting the confidentiality of this proprietary information belonging to the Sponsor.

15.8.4 *Publication*

The results from this study may be submitted for publication at the discretion of the Sponsor.

15.9 Archiving

The Principal Investigator is responsible for maintaining essential documents, (as defined in ICH E6 GCP, Section 8) for at least 15 years after finalization of the CSR. This includes any original source documents related to the study, the Subject Identification List (providing the sole link between named subject source records and anonymous eCRF data), the original signed ICFs and detailed records of disposition of IMP.

It is the responsibility of the Sponsor to inform the Investigator/institution as to when these documents no longer need to be retained.

The Sponsor will archive the TMF in accordance with ICH E6 GCP, Section 8 and applicable regulatory requirements.

The data from the eCRFs will be sent to the Sponsor and a copy will be sent to the research clinic and filed in the Investigator Site File for archiving for 15 years after finalization of the CSR.

The completed original eCRFs are the sole property of the Sponsor and should not be made available in any form to third parties, except for authorized representatives of appropriate Health/Regulatory Authorities, without written permission from the Sponsor.

16 DATA MANAGEMENT

The data management routines include procedures for handling of the eCRF, database set-up and management, data entry and verification, data validation, QC of the database, and documentation of the performed activities including information of discrepancies in the

process. The database, data entry screens, and program will be designed in accordance with the CSP.

Data validation/data cleaning procedures are designed to assure validity and accuracy of clinical data. These procedures consist of computerized online edit checks identifying e.g. data values that are outside the allowed range and SAS-programmed offline checks on data exports. All study-specific and standard data validation programming will be tested in a separate testing environment prior to use on production data.

Detailed information on data management will be described in a study-specific Data Management Plan (DMP).

16.1 The web based eCRF

All clinical data will be entered into a 21 CFR Part 11-compliant eCRF (Viedoc™) provided by PCG Solutions AB. The eCRF includes password protection and internal quality checks, such as automatic range checks, to identify data that appear inconsistent, incomplete, or inaccurate. Clinical data will be entered directly from the source documents or at bedside (if the eCRF data constitutes source data). Source data are to be defined at the site before inclusion of the first subject (Section 15.4).

Authorized site personnel designated by the Investigator will complete data collection. Appropriate training and security measures will be completed with the Investigator and all authorized trial site personnel prior to the trial being initiated and any data being entered into the system for any trial subject.

16.2 The entering of data into the eCRF

All data should be entered in English. The eCRFs should be completed as soon as possible during or after the subject's visit. The Investigator must verify that all data entries in the eCRFs are accurate and correct. If some assessments are not done, or if certain information is not available, not applicable or unknown, the Investigator or assigned clinical staff should record such information in the eCRF. The Investigator will be required to electronically sign off the clinical data. This will be performed by means of the Investigator's unique UserID and password; date and time stamps will be added automatically at time of electronic signature.

16.3 The query process

The Monitor will review the eCRFs and evaluate them for completeness and consistency. Data in the eCRF will be compared with the respective source documents to ensure that there are no discrepancies for critical data as described in the RBM plan. All entries, corrections, and alterations are to be made by the Investigator or designee. Neither the Monitor nor any other study team member besides site staff can enter data in the eCRF.

If corrections are needed, queries will be raised within the eCRF. An appropriate member of the site staff will answer the queries in the eCRF either by correcting the data or by entering a response to the query.

16.4 Audit trail

All entries in the eCRF will be fully recorded in a protected audit trail. Once clinical data have been saved, corrections to the data fields will be audit trailed, meaning that the reason for change, the name of the person who made the change, together with time and date will be logged.

16.5 External data

External data consists of data that are not recorded in the eCRF. Data may be received in electronic format or as a paper printout. Key variables are defined in order to uniquely identify each sample record. File and data formats are agreed with the external data provider.

External data in the present study comprise safety laboratory data (Section 11.7.5), data from the ADA analysis (Section 11.7.8), CXCL12 exposure data (plasma and blood culturing; Section 11.8.1), immunohistochemistry/histopathological data (Section 11.8.2), skin swab data (Section 11.8.6) and feces data (Section 11.8.7), 3D imaging data (Section 11.9.1), microcirculation data (Section 11.9.2) and microbiome analysis data (Section 11.9.3).

16.6 Medical coding

Medical coding will be performed by trained personnel at [redacted]. AEs and medical/surgical history verbatim terms are coded using the Medical Dictionary of Regulatory Activities (MedDRA; latest version available at the start of the study). Prior and concomitant medications will be coded according to the WHO Anatomic Therapeutic Chemical (ATC) classification system. All coding will be approved by Sponsor prior to DBL.

16.7 Database lock

When all data have been entered and discrepancies solved, clean file will be declared, the database will be locked, the code will be broken and the data will be analyzed.

Interim reports based on unblinded data will be prepared after completion of the 6 weeks visit (Visit 7 [SAD part] and Visit 14 [MAD part] and after completion of the 12 months visits (Visit 10 [SAD part] and Visit 17 [MAD part]; one report with combined 12 months SAD and MAD data). The data for the reports will be produced from exports of the cleaned database but no formal DBL will be performed until after the last subject has performed the last 5-year follow-up assessment. A complete CSR including will be written following the final DBL.

All clinical staff involved in data evaluation and all independent evaluators will be kept blinded to treatment.

17 STATISTICAL METHODS AND DETERMINATION OF SAMPLE SIZE

The principal features of the statistical analysis to be performed are described in this section. A more technical and detailed elaboration of the principal features will be presented in a separate Statistical Analysis Plan (SAP), which will be signed and approved prior to each planned interim report.

Analyses of the primary and secondary endpoints will be performed by [redacted]

17.1 General

Data will be presented using summary statistics in terms of number (N), arithmetic mean, standard deviation (SD), median, minimum and maximum value as appropriate.

Categorical data will be presented as counts and percentages. When applicable, summary data will be presented by treatment, and by assessment time. Individual subject data will be listed by subject number, treatment, and, where applicable, by assessment time.

All descriptive summaries and statistical analyses will be performed using SAS Version 9.4 or later (SAS Institute, Inc., Cary, NC).

Baseline will be defined as the visit with last data collection point prior to the first administration of IMP.

To minimize inter-rater reliability between independent experts, pre-study training and rating sessions will be conducted. The experts will be supplied with photos of wounds to rate. The correlation between the ratings will be analyzed using Kappa statistics. If the correlation between the different experts is below 0.8, new rating sessions will be performed until the correlation is above 0.8.

17.2 Determination of sample size

No formal sample size calculation has been performed. The proposed sample size is considered sufficient to provide adequate information for the study objectives.

17.3 Analysis data sets

17.3.1 *Full analysis set*

The Full Analysis Set (FAS) will consist of all subjects who have been randomized and received at least one dose of IMP. This population will be used as the Safety analysis set.

There will be one FAS for each part.

17.3.2 *Per protocol set*

The Per Protocol Set (PPS) will consist of all subjects who have been randomized and completed the study without any major protocol deviations that are judged to compromise the analysis of the data. All protocol violations will be judged as major or minor at the clean file meeting.

17.4 Description of study population

17.4.1 *Demographics and baseline characteristics*

Descriptive statistics for demographics, weight and height will be presented by treatment and cohort using descriptive statistics and listings.

All data will be listed by cohort and subject.

17.4.2 *Medical/surgical history and prior/concomitant medication*

Medical/surgical history and prior/concomitant medications will be presented by cohort and overall using descriptive statistics and listings.

All data will be listed by cohort and subject.

17.4.3 *Treatment compliance*

The number of subjects treated in each cohort, and their treatments will be tabulated.

All data will be listed by cohort and subject.

17.5 Analysis of safety endpoints (primary endpoints)

17.5.1 *Adverse events*

An overview of all AEs, including SAEs, intensity, relationship to IMP, and deaths will be presented by SOC and preferred term (PT).

Incidence of AEs and SAEs will be summarized by SOC and PT by cohort and overall.

All AE data will be listed by cohort and subject and include the verbatim term entered by the Investigator.

17.5.2 *Physical examination*

Clinically significant and non-clinically significant abnormal findings will be specified and presented by subject and summarized by cohort and visit.

Changes over time will be presented using shift tables.

All data will be listed by cohort and subject.

17.5.3 *Vital signs*

Vital signs (systolic/diastolic blood pressure, pulse and temperature) will be summarized by cohort and visit. Data will be presented with absolute and percent change from baseline.

All data will be listed by cohort and subject.

17.5.4 *12-lead ECG*

All ECGs will be categorized as ”normal”, ”abnormal, not clinically significant”, or ”abnormal, clinically significant” (as judged by the Investigator) and summarized by cohort and visit using frequency tables.

Changes over time will be presented using shift tables.

All data will be listed by cohort subject.

17.5.5 *Safety laboratory analyses*

Safety laboratory data will be summarized by cohort and visit with absolute and percent change from baseline.

Abnormal, clinically significant values will be summarized separately if considered appropriate.

All data will be listed by cohort and subject.

17.5.6 *Local tolerability*

The following parameters are will be measured for local tolerability:

- Appearance of wound and wound edge (inflammation)
- Condition of skin surrounding the wound (inflammation)
- Hemorrhage
- Amount of exudate present
- Presence of slough/necrotic tissue
- Presence of granulation tissue
- Hypergranulation

Each variable will be presented using frequency tables by cohort, treatment and visit.

Shift tables will also be presented by cohort, treatment and visits if considered appropriate.

Subject assessment of pruritus and pain will be analyzed as above.

All data will be listed by cohort, treatment and subject.

17.5.7 *Anti-drug-antibody analysis*

The incidence, observed proportion of subjects testing positive for ADA, and their titer will be presented by cohort and visit.

All data will be listed by cohort and subject.

17.6 Analysis of secondary endpoints

17.6.1 *CXCL12 levels in blood and in the wound*

Relationship between dose of activated ILP100-DP and levels of CXCL12 in wound biopsies and blood (SAD part only) will be presented by correlation analysis.

Relationship between dose of activated ILP100-DP and the time course of complete wound healing/closure (SAD and MAD parts) will be presented by correlation analysis.

Actual CXCL12 levels in the wound (SAD only) and in the blood (SAD and MAD) together with absolute and percent change will be presented using summary statistics.

L. reuteri R2LC colonies following blood culture will be presented using summary statistics by treatment, visit and cohort.

The colonies will be presented both as actual values and by logarithm scale (10 log).

All data will be listed by cohort, treatment (biopsy data) and subject.

17.6.2 *Histology analysis*

Histological parameters will be assessed by quantitative or semi-quantitative methods frequently applied in the field. Detailed description of the analysis will be provided in the SAP.

All data will be listed by cohort, treatment and subject.

17.6.3 *Wound healing*

Wound healing will be assessed as yes or no at each visit and presented using frequency tables by treatment, visit and cohort. Dose response relationship will be presented by correlation analysis. Comparison between active and placebo treatment will be performed by non-parametric statistics.

All data will be listed by cohort, treatment and subject.

17.6.4 *Wound area*

The reduction in wound area will be presented using summary statistics by treatment, visit and cohort. Dose response relationship will be presented by correlation analysis. Comparison between active and placebo treatment will be performed by ANOVA.

All data will be listed by cohort, treatment and subject.

17.6.5 *Scar formation*

Overall scar tissue formation (normal/abnormal) will be presented using summary statistics by treatment, visit and cohort.

Each individual item will be presented using frequency tables by cohort, treatment and visit. The total score will be presented using summary statistics by cohort, treatment and visit.

All data will be listed by cohort, treatment and subject.

17.6.6 *Detection of L. reuteri colonies on skin*

L. reuteri R2LC colonies on skin will be presented using summary statistics by treatment, visit and cohort.

The colonies will be presented both as actual values and by logarithm scale (10 log).

All data will be listed by cohort, treatment and subject.

17.6.7 *Detection of L. reuteri colonies in feces*

L. reuteri R2LC colonies in feces be presented using summary statistics by treatment, visit and cohort.

The colonies will be presented both as actual values and by logarithm scale (10 log).

All data will be listed by cohort and subject.

17.7 Analysis of exploratory endpoints

The exploratory endpoints may not be reported in the CSR.

17.7.1 *Changes in wound and scar appearance by 3D imaging*

3D imaging data will be presented using summary statistics by treatment, visit and cohort.

All data will be listed by cohort, treatment and subject.

17.7.2 *Microcirculation analysis*

Data will be presented using summary statistics by treatment, visit and cohort.

All data will be listed by cohort, treatment and subject.

17.7.3 *Wound microbiome alterations*

Data will be presented using summary statistics by treatment, visit and cohort.

All data will be listed by cohort, treatment and subject.

18 REFERENCES

Armstrong, D.G., Wrobel, J., and Robbins, J.M. (2007). Guest Editorial: are diabetes-related wounds and amputations worse than cancer? Int. Wound J. 4, 286–287.

Badillo, A.T., Chung, S., Zhang, L., Zoltick, P., and Liechty, K.W. (2007). Lentiviral Gene Transfer of SDF-1α to Wounds Improves Diabetic Wound Healing. J. Surg. Res. 143, 35–42.

Beider, K., Bitner, H., Leiba, M., Gutwein, O., Koren-Michowitz, M., Ostrovsky, O., Abraham, M., Wald, H., Galun, E., Peled, A., et al. (2014). Multiple myeloma cells recruit tumor-supportive macrophages through the CXCR4/CXCL12 axis and promote their polarization toward the M2 phenotype. Oncotarget 5, 11283–11296.

Bianchi, M.E. (2007). DAMPs, PAMPs and alarmins: all we need to know about danger. J. Leukoc. Biol. 81, 1–5.

Bollag, W.B., and Hill, W.D. (2013). CXCR4 in Epidermal Keratinocytes: Crosstalk within the Skin. J. Invest. Dermatol. 133, 2505–2508.

Boniakowski, A.E., Kimball, A.S., Jacobs, B.N., Kunkel, S.L., and Gallagher, K.A. (2017). Macrophage-Mediated Inflammation in Normal and Diabetic Wound Healing. J. Immunol. 199, 17–24.

Bootun, R. (2013). Effects of immunosuppressive therapy on wound healing. Int. Wound J. 10, 98–104.

EudraLex VOLUME 4, Good manufacturing practices, ANNEX 13, Manufacture of investigational medicinal, products, February 2010, section 26.

FDA. Guidance for industry: Toxicity grading scale for healthy adult and adolescent volunteers enrolled in preventive vaccine clinical trials (2007).

Feng, G., Hao, D., and Chai, J. (2014). Processing of CXCL12 impedes the recruitment of endothelial progenitor cells in diabetic wound healing. FEBS J. 281, 5054–5062.

Goren, I., Allmann, N., Yogev, N., Schürmann, C., Linke, A., Holdener, M., Waisman, A., Pfeilschifter, J., and Frank, S. (2009). A Transgenic Mouse Model of Inducible Macrophage Depletion. Am. J. Pathol. 175, 132–147.

Gottrup, F. (2004). A specialized wound-healing center concept: importance of a multidisciplinary department structure and surgical treatment facilities in the treatment of chronic wounds. Am. J. Surg. 187, S38–S43.

Guest, J.F., Ayoub, N., McIlwraith, T., Uchegbu, I., Gerrish, A., Weidlich, D., Vowden, K., and Vowden, P. (2015). Health economic burden that wounds impose on the National Health Service in the UK. BMJ Open 5, e009283.

Gurtner, G.C., Werner, S., Barrandon, Y., and Longaker, M.T. (2008). Wound repair and regeneration. Nature 453, 314–321.

Heyer, K., Herberger, K., Protz, K., Glaeske, G., and Augustin, M. (2016). Epidemiology of chronic wounds in Germany: Analysis of statutory health insurance data: Epidemiology of chronic wounds in Germany. Wound Repair Regen. 24, 434–442.

Holzapfel, W.H., Haberer, P., Geisen, R., Björkroth, J., and Schillinger, U. (2001). Taxonomy and important features of probiotic microorganisms in food and nutrition. Am. J. Clin. Nutr. 73, 365s–373s.

van der Laan, J.W., Brightwell, J., McAnulty, P., Ratky, J., and Stark, C. (2010). Regulatory acceptability of the minipig in the development of pharmaceuticals, chemicals and other products. J. Pharmacol. Toxicol. Methods 62, 184–195.

Laguri, C., Sadir, R., Rueda, P., Baleux, F., Gans, P., Arenzana-Seisdedos, F., and Lortat-Jacob, H. (2007). The Novel CXCL12γ Isoform Encodes an Unstructured Cationic Domain Which Regulates Bioactivity and Interaction with Both Glycosaminoglycans and CXCR4. PLoS ONE 2, e1110.

Lambeir, A.-M., Proost, P., Durinx, C., Bal, G., Senten, K., Augustyns, K., Scharpé, S., Van Damme, J., and De Meester, I. (2001). Kinetic Investigation of Chemokine Truncation by CD26/Dipeptidyl Peptidase IV Reveals a Striking Selectivity within the Chemokine Family. J. Biol. Chem. 276, 29839–29845.

Lipsky, B.A., Berendt, A.R., Cornia, P.B., Pile, J.C., Peters, E.J.G., Armstrong, D.G., Deery, H.G., Embil, J.M., Joseph, W.S., Karchmer, A.W., et al. (2013). 2012 infectious diseases society of america clinical practice guideline for the diagnosis and treatment of diabetic foot infections. J. Am. Podiatr. Med. Assoc. 103, 2–7.

Lucas, T., Waisman, A., Ranjan, R., Roes, J., Krieg, T., Muller, W., Roers, A., and Eming, S.A. (2010). Differential Roles of Macrophages in Diverse Phases of Skin Repair. J. Immunol. 184, 3964–3977.

Mercenier, A., Pavan, S., and Pot, B. (2003). Probiotics as biotherapeutic agents: present knowledge and future prospects. Curr. Pharm. Des. 9, 175–191.

National Cancer Institute Cancer Therapy Evaluation Program. Common terminology criteria for adverse events, CTCAE v5.0 (2017).

NIH. Division of AIDS table for grading the severity of adult and pediatric adverse events (2004).

Norlund, A., Apelqvist, J., Bitzén, P.O., Nyberg, P., and Scherstén, B. (2001). Cost of illness of adult diabetes mellitus underestimated if comorbidity is not considered. J. Intern. Med. 250, 57–65.

OECD Diabetes prevalence and incidence , in Health at a Glance: Europe 2012. OECD Publ. 2012, 42–43.

Orlova, V.V., Choi, E.Y., Xie, C., Chavakis, E., Bierhaus, A., Ihanus, E., Ballantyne, C.M., Gahmberg, C.G., Bianchi, M.E., Nawroth, P.P., et al. (2007). A novel pathway of HMGB1-mediated inflammatory cell recruitment that requires Mac-1-integrin. EMBO J. 26, 1129–1139.

Phillipson, M., and Kubes, P. (2011). The neutrophil in vascular inflammation. Nat. Med. 17, 1381–1390.

Proost, P., Struyf, S., Schols, D., Durinx, C., Wuyts, A., Lenaerts, J.P., De Clercq, E., De Meester, I., and Van Damme, J. (1998). Processing by CD26/dipeptidyl-peptidase IV reduces the chemotactic and anti-HIV-1 activity of stromal-cell-derived factor-1alpha. FEBS Lett. 432, 73–76.

Rathmann, W., and Giani, G. (2004). Global prevalence of diabetes: estimates for the year 2000 and projections for 2030. Diabetes Care 27, 2568–2569; author reply 2569.

Sanchez-Martin, L., Estecha, A., Samaniego, R., Sanchez-Ramon, S., Vega, M.A., and Sanchez-Mateos, P. (2011). The chemokine CXCL12 regulates monocyte-macrophage differentiation and RUNX3 expression. Blood 117, 88–97.

Sen, C.K., Gordillo, G.M., Roy, S., Kirsner, R., Lambert, L., Hunt, T.K., Gottrup, F., Gurtner, G.C., and Longaker, M.T. (2009). Human skin wounds: A major and snowballing threat to public health and the economy. Wound Repair Regen. 17, 763–771.

Sibille, M., Patat, A., Caplain, H., and Donazzolo, Y. (2010). A safety grading scale to support dose escalation and define stopping rules for healthy subject first-entry-into-man studies: Some points to consider from the French Club Phase I working group. Br. J. Clin. Pharmacol. 70, 736–748.

Sozzani, S., Sallusto, F., Luini, W., Zhou, D., Piemonti, L., Allavena, P., Van Damme, J., Valitutti, S., Lanzavecchia, A., and Mantovani, A. (1995). Migration of dendritic cells in response to formyl peptides, C5a, and a distinct set of chemokines. J. Immunol. Baltim. Md 1950 155, 3292–3295.

Stricker-Krongrad, A., Shoemake, C.R., Liu, J., Brocksmith, D., and Bouchard, G. (2017). The importance of minipigs in dermal safety assessment: an overview. Cutan. Ocul. Toxicol. 36, 105–113.

Swedish Goverment Department of Medical Evaluation (2014). SBU Svårläkta sår hos äldre - prevention och behandling. En systematisk litteraturöversikt.

Wise, R., Hart, T., Cars, O., Streulens, M., Helmuth, R., Huovinen, P., and Sprenger, M. (1998). Antimicrobial resistance. Is a major threat to public health. BMJ 317, 609–610.

Vågesjö, E., Öhnstedt, E., Mortier, A., Lofton, H., Huss, F., Proost, P., Phillipson, M. (2018). Accelerated wound healing in mice by on-site production and delivery of CXCL12 by transformed lactic acid bacteria. Proceedings of the National Academy of Sciences, 115(8), 1895–1900.

WHO (2004). Laboratory biosafety manual (Geneva: World Health Organization).

WHO (1981) Recommendations for grading of acute and subacute toxicity: reporting the results of cancer treatment. Cancer, 47:207-214.

19 APPENDICES

19.1 Signatures

19.1.1 *Principal investigator statement*

I have read and understood this CSP and agree to conduct the study accordingly and to comply with the investigator obligations stated in this CSP, GCP and applicable regulatory requirements.

Principal investigator

[redacted]

Name	*Signature*	*Date*

[redacted]

Site

19.1.2 *Signature page (approval of the clinical study protocol)*

Sponsor signatory

Evelina Vågesjö, CEO Ilya Pharma AB		
Name	*Signature*	*Date*

19.2 Declaration of Helsinki

http://www.up.ac.za/media/shared/Legacy/sitefiles/file/45/2875/declarationofhelsinki_fortaleza_brazil2013.pdf
