# Supplementary material for: Engineered bacteria to accelerate wound healing: an adaptive, randomised, double-blind, placebo-controlled, first-in-human phase 1 trial
Source: eClinicalMedicine. 2023 May 25;60:102014. doi: 10.1016/j.eclinm.2023.102014 (PMC10220316; doi:10.1016/j.eclinm.2023.102014)
Supplement: situ-safe-ip-ct-001-sap-summary-of-changes [file mmc6.pdf]

Summary of protocol changes in Ilya Pharma IP-CT-001 study

During the conduct of the 12 months of the SAD part of the IP-CT-001 trial, there were no substantial amendment, but 3 non-substantial amendments to the CSP version 2.0 dated 14JUN2019. No CSP updates was performed as a consequence of the amendments. A summary of non-substantial amendments is provided in **Table 1.**

During the conduct of the 12 months of the MAD part of the IP-CT-001 trial, there were one substantial amendment to the CSP version 2.0 dated 14JUN2019, one substantial amendment (change in Principal Investigator; no change in CSP) to the CSP version 3.0 dated 24FEB2020 and 3 non-substantial amendments as outlined in **Table 1.**

The CSP versions 2.0 and 3.0 is provided. Version 1.0 of the CSP was never used in the study. Version 1.0 was updated to version 2.0 as a result of questions raised by the MPA and the EC prior to start of the SAD part.

Table 1 Summary of protocol changes in the conduct of the SAD and MAD part of the IP-CT-001 study

Change	Key details of change	Rationale
Amendment No. 1 Non-substantial 10OCT2019	Revision of blood sampling time window for CXCL12 analysis.<br><br>Administrative changes.	Logistics.
Amendment No. 2 18DEC2019 (typo corrected 12FEB2020) Non-substantial	No change to the protocol. Non-substantial amendment to document decision of starting dose in MAD: $5x10^5$ CFU/cm^2 wound area (same dose as tentatively specified in the CSP).<br><br>The planned (and actual) doses to be administered in cohorts 2 and 3 of the MAD were $5x10^7$ and $1x10^9$ CFU/cm2 wound area, which are also in line with the CSP.	Documentation purposes.
Amendment No. 3 Non-substantial 16DEC2019	Randomization change. Instead of randomizing by IMP in the MAD part, i.e., randomization of active treatment, placebo and saline to 8 wounds (4 on each arm), it was decided that active treatment would be randomized to 4 wounds on one arm and placebo and saline to 4 wounds on the other arm.	To avoid interference from local tolerability reactions from one or several wounds possibly being related to active treatment on placebo or saline treated wounds.

Change	Key details of change	Rationale
Amendment No. 4 Substantial 24FEB2020<br><br>New protocol version: version 3.0 24FEB2020	Change of dressing procedures:<br>In cohort 1 the wounds were covered with adhesive, transparent film throughout the treatment period (Day 1 to Day 19). In cohorts 2 and 3, the wounds were covered with adhesive, transparent film during 48 hours after the first and second IMP application. From Day 3 and onwards, the wounds were treated with IMP and then covered with adhesive, transparent film for 1 hour only. Thereafter the film was removed, the wounds were allowed to air dry and were then be covered with non-occlusive dressing in accordance with standard wound care procedures.	In MAD cohort 1, all wounds were covered with an adhesive, transparent film throughout the treatment period. The purpose of the covering was to protect the wounds and isolate them from each other, minimizing the risk for leakage of IMP between wounds. The moist environment created below each film is, however, sub-optimal for wound healing and may contribute to the appearance of eczema and/or inflammatory reactions and to the growth of skin bacteria other than L. reuteri R2LC, which may cause infections.<br><br>During MAD cohort 1, the 8 subjects reported 58 wound-related AEs up until and including Day 21 (2 days after the last IMP administration). The most common AEs were administration site eczema (16 events reported by 7 subjects), wound infection (10 events reported by 3 subjects) and intermittent wound hemorrhage (9 events reported by 3 subjects).<br><br>Most events were late onset events and started approximately 9 to 14 days after the first IMP application. Four of the eight subjects were affected on both arms, i.e., reactions were present both in wounds

Change	Key details of change	Rationale
	Non-substantial changes<br>Definition of AEs:<br>Bleeding and/or mild, transient inflammation observed during the early local tolerability evaluations, and when judged by the Investigator to be caused by a study procedure performed pre-treatment (i.e., due to biopsy) were to be reported as procedure-related in the eCRF. Bleeding, inflammation and infection assessed as non-procedure related were to be reported AEs.<br><br>AE-reporting:<br>Clarification of wound evaluation criteria regarding hemorrhage was made at the end of the SAD part. It was decided that, for the MAD part, coagulated blood were not be reported as hemorrhage, bleeding assessed at several visits were to be reported as intermittent bleeding, exceptional persistent inflammation were to be reported as AE.<br>Clarification that persistent pain or pruritus reported by subject were to be reported as AEs.<br><br>Microcirculation measurements:<br>During the training of the equipment the Sponsor decided that 2 wounds rather than 1 could be assessed simultaneously over an area of approximately 5x10 cm.	treated with active drug and wounds treated with saline/placebo. Therefore, it was concluded that some, or most, wound-related AEs could be related to the wound treatment procedure rather than related to the IMP.<br><br>Change in AE definition and AE-reporting:<br>To more accurately capture the occurrence of wound hemorrhage and other treatment-related AEs.

Change	Key details of change	Rationale
	Timing of CXCL12 sampling:<br>Previous version of the CSP: CXCL12 blood sampling prior to wound punching and pre-treatment between 8 and 10 AM.<br>New version of the CSP: CXCL12 blood sampling prior to wound punching and pre-treatment at 9:00±2 hours.<br>In addition, the timing of sampling on Days 3, 5, 8, 15 and 19 was changed from 8 to10 AM to 9:00±2 hours but no later than 11:00 AM.	
Amendment No. 5 Substantial 11MAR2020	Change of the Principal Investigator conducting cohort 1 to a new Principal Investigator for cohorts 2 and 3.	Principal Investigator changed employment.
File Note 20JAN2020	The wound healing assessments Day 17 by the Independent Evaluators were moved to Day 32, i.e., local tolerability, wound healing, wound area and scar tissue formation were assessed at Day 32 rather than Day 17.	Extension of the wound evaluation period for Independent Evaluators to ensure capturing of the complete wound healing process.
File Note 18MAY2020	From cohort 2, dressing was only collected for microbiome analysis on Day 2 (applied Day 1) and Day 3 (applied Day 2), i.e., dressings were NOT collected Day 8 (applied Day 5), Day 15 (applied Day 12) and Day 21 (applied Day 19).	Wound treatment procedure was revised starting from MAD cohort 2.
File Note 09DEC2020	The consensus decisions were documented in the eCRF as a separate visit for Wound Evaluation Committee (WEC) for each affected subject. Individual results from all evaluators and WEC assessment results are used in the summary statistics for wound area.	Due to discrepancies in the assessments of wound area measurements between the Independent Evaluators, Sponsor established a WEC that performed an adjudicated evaluation of wound area measurement of the photos where the individual measurements of the wound areas by the 3 Independent Evaluators were too diverse, (>2,5 standard deviation (SD) from the mean areas of

Change	Key details of change	Rationale
		all wounds at the same anatomical location at the same visit).
